# MtDNA analysis reveals enriched pathogenic mutations in Tibetan highlanders

**DOI:** 10.1038/srep31083

**Published:** 2016-08-08

**Authors:** Longli Kang, Hong-Xiang Zheng, Menghan Zhang, Shi Yan, Lei Li, Lijun Liu, Kai Liu, Kang Hu, Feng Chen, Lifeng Ma, Zhendong Qin, Yi Wang, Xiaofeng Wang, Li Jin

**Affiliations:** 1 Key Laboratory for Molecular Genetic Mechanisms and Intervention Research on High Altitude Disease of Tibet Autonomous Region; Key Laboratory of High Altitude Environment and Gene Related to Disease of Tibet Ministry of Education, School of Medicine, Xizang University for Nationalities, Xianyang, China; 2 Ministry of Education Key Laboratory of Contemporary Anthropology and Center for Evolutionary Biology, School of Life Sciences and Institutes of Biomedical Sciences, Fudan University, Shanghai, China; 3 Tibet Occupational College of Technology, Lhasa, Tibet, China

## Abstract

Tibetan highlanders, including Tibetans, Monpas, Lhobas, Dengs and Sherpas, are considered highly adaptive to severe hypoxic environments. Mitochondrial DNA (mtDNA) might be important in hypoxia adaptation given its role in coding core subunits of oxidative phosphorylation. In this study, we employed 549 complete highlander mtDNA sequences (including 432 random samples) to obtain a comprehensive view of highlander mtDNA profile. In the phylogeny of a total of 36,914 sequences, we identified 21 major haplogroups representing founding events of highlanders, most of which were coalesced in 10 kya. Through founder analysis, we proposed a three-phase model of colonizing the plateau, i.e., pre-LGM Time (30 kya, 4.68%), post-LGM Paleolithic Time (16.8 kya, 29.31%) and Neolithic Time (after 8 kya, 66.01% in total). We observed that pathogenic mutations occurred far more frequently in 22 highlander-specific lineages (five lineages carrying two pathogenic mutations and six carrying one) than in the 6,857 haplogroups of all the 36,914 sequences (P = 4.87 × 10^−8^). Furthermore, the number of possible pathogenic mutations carried by highlanders (in average 3.18 ± 1.27) were significantly higher than that in controls (2.82 ± 1.40) (P = 1.89 × 10^−4^). Considering that function-altering and pathogenic mutations are enriched in highlanders, we therefore hypothesize that they may have played a role in hypoxia adaptation.

The Tibetan Plateau, with an average altitude of above 4,000 meters, is the highest area in the world. It poses one of the extreme environments with hypobaric hypoxia for human beings, given the fact that there is ~40% less oxygen in the air than at sea level^1^. Tibetan highlanders, including Tibetans, Monpas, Lhobas, Dengs and Sherpas, whose ancestors began to colonize the Tibetan Plateau before the Last Glacial Maximum (LGM) even ~30 thousand years ago (kya)^2^, are considered to have been well adapted to the challenge of severe hypoxia in the Tibetan Plateau. How Tibetan highlanders colonized the plateau and adapted to the extreme environments has appealed growing academic interests in recent years and becomes the major question for genetic study on the highlanders.

Genome-wide studies have shown probable candidates of hypoxia adaptation for Tibetan populations^3^,^4^,^5^,^6^,^7^,^8^,^9^. The *EPAS1* (also known as *HIF-2α*, encoding hypoxia-inducible factor 2α subunit) and *EGLN1* (also known as *PHD2*, encoding prolyl hydroxylase domain protein 2) loci, both associated with hypoxia-inducible factor (HIF) pathway, have been identified to be under positive natural selection and important for hypoxia adaptation. Mitochondria, the center of metabolism regulation, consume oxygen and generate reactive oxygen species (ROS) which have further effect on HIF signaling^10^,^11^. Thus, mitochondria and mitochondrial DNA (mtDNA), the genetic materials of mitochondria, are regarded as sensible targets for studying hypoxia adaptation in Tibetan highlanders.

MtDNA is a well-known genetic marker for its high mutation rate, maternal inheritance, and lack of recombination^12^. Mutations in mtDNA are accumulated in series and mtDNA phylogeny of Tibetan highlanders could be properly reconstructed. MtDNA encodes 13 core subunits of oxidative phosphorylation (OXPHOS), which helps mitochondria to supply ~90% of the energy of human body demand. Radical mtDNA variations would have considerable effect on OXPHOS and even cause maternal genetic diseases like Leber’s hereditary optic neuropathy (LHON), deafness, mitochondrial encephalomyopathy with lactic acidosis and stroke-like episodes (MELAS), and etc.^13^,^14^. Several studies have reported that the pathogenic mutations in lowland populations showed signals of being under positive selection in Tibetans and Sherpas, indicating that these radical and function-altering variants might have effect on OXPHOS and further contribute to hypoxia adaptation for Tibetan highlanders^11^,^15^. However, there is still no systematic evaluation on the function-altering and pathogenic mutations in mtDNA genomes involving different ethnic groups of the Tibetan Plateau.

In this study, we analyzed 549 highlander sequences (i.e. Tibetans, Lhobas, Dengs, Monpas and Sherpas) including 293 newly sequenced ones, to obtain a comprehensive view of mtDNA profile of Tibetan highlanders. An mtDNA phylogeny was constructed using a total of 36,914 sequences of world populations including highlander sequences. Through the founder analysis based on 432 random highlander samples, we proposed a three-phase model of colonizing the Tibetan Plateau, i.e., pre-LGM Time (30 kya, 4.68%), post-LGM Paleolithic Time (16.8 kya, 29.31%) and Neolithic Time (after 8 kya, 66.01% in total). Next, we observed that pathogenic mutations occurred far more frequently in 22 highlander-specific lineages (five lineages carrying two pathogenic mutations and six carrying one) than in the 6,857 haplogroups of all the 36,914 sequences (P = 4.87 × 10^−8^). In addition, possible pathogenic mutations in Tibetan highlanders were significantly higher than those in controls from East Asians of 1000 Genomes Projects using different strategies. Thus, considering that function-altering and pathogenic mutations are enriched in highlanders, we hypothesize that they may have played a role in adaptation of hypoxic environments for highlanders.

## Results

### Overview of mtDNA in Tibetan highlanders

We collected and sequenced 285 Tibetan highlanders (i.e., Tingri Tibetans, Lhobas, Dengs and Monpas) and generated high quality data of the complete mtDNA sequences (average coverage being 3,402× and minimum coverage 186×). In addition, we obtained 33 complete mitochondrial sequences of Tibetans from fastq files of Yi *et al.* (average coverage being 15× and minimum coverage 6×)^9^, and collected all 223 Tibetan highlander sequences (including Sherpas, Monpas and Tibetans) available in the literatures (detail information of these samples was tabulated in Table S1). Eight additional Tibetan sequences generated in our lab were also included for phylogenetic analysis as Tibetan highlanders. In total, 549 mtDNA sequences of Tibetan highlanders were analyzed in this study, of which 432 individuals were randomly sampled from 7 highland populations, including 86 Tingri Tibetans, 91 Lhobas, 91 Dengs, 17 Monpas, 76 Sherpas, 38 Tibetans from Ji *et al.*^11^ (referred as Tibetan1) and 33 Tibetans from Yi *et al.*^9^ (referred as Tibetan2). Finally, 293 mitochondrial genomes were deposited in Genbank (KT725860-KT726152). Variations to rSRS of 33 Tibetan mtDNA from fastq files of Yi *et al.* were attached as supplemental data (Table S2). Haplogroup assignments were consulted to Phylotree Build 16^16^.

Overall 432 random samples from 7 highlander populations consisted of major East Asian haplogroups, e.g., A (13.65%), R9 (10.18%), M9 (27.08%), D (18.28%), M8 (7.86%), M12’G (6.71%) (Table S3). It was noteworthy that Haplogroup M9 was the most frequent haplogroup in the highlanders collectively, as well as in Dengs, Monpas, Tingri Tibetans, Tibetan1 and Tibetan2, respectively. In Lhobas, Haplogroup F achieved the highest frequency while Haplogroup M9 was the second common. MDS analysis showed that 7 highland populations were clustered together, and close to other East Asian populations (Figure S1). Tajima’s D as well as Fu & Li’s D^*^ and F* tests showed significant negative results in 6 highland populations except for Monpas, suggesting possible population expansions or positive selection (Table S4), while Bayesian skyline plots (BSP) indicated recent population bottlenecks in most highland populations (Figure S2).

### MtDNA phylogeny of Tibetan highlanders

To identify autochthonous lineages and variants in Tibetan highlanders, a maximum parsimony phylogeny of 36,914 worldwide complete mtDNA sequences including 549 highlander sequences were reconstructed, which was further confirmed by median-joining network. In the reconstructed phylogeny, 21 haplogroups were considered as major haplogroups of Tibetan highlanders since these haplogroups accounted for considerably high frequencies in 432 random highlanders or appeared in at least three ethnic groups of highlanders (in total 63.19% in 432 random samples, Figures S3 and S4 and Table 1). For these major haplogroups are derived from the most recent common ancestor shared with non-highlander populations in the phylogeny respectively, they represent possible distinct founding events by ancestors migrating to the Tibetan Plateau.

Coalescence time of the 21 major haplogroups of Tibetan highlanders was estimated employing ρ-statistic based method and maximum likelihood method (See details in Methods). Haplogroup M62 was the oldest lineage (~24–26 kya ago) (Table 1). Three haplogroups (Haplogroups M13a2, D4j1b and A6) showed coalescence time between 18~10 kya, while the remaining 17 lineages (Haplogroups G3b1, M33b1a1, C4a3b, Z3b, D4h1c1a1, D4j1a1, D5a2c, M9a1a1c1b, M9a1a2, M9a1b1c, M9a1b1d, A11a, A15c1a, F1c1a2a1, F1d1a, F1d5 and F1g) coalesced less than 10 kya. Interestingly, Deng-specific lineages (Haplogroups M33b1a1 and D4h1c1a1), Sherpa-specific lineage (Haplogroup A15c1a) and Lhoba-specific lineage (Haplogroup F1c1a2a1) were derived very recently (after 7 kya). Of the 21 major haplogroups, those coalesced within 10 kya encompassing 57.41% of the highlanders collectively suggests that gene flows to the Tibetan Plateau may have occurred mainly after 10 kya.

**Figure 1:**
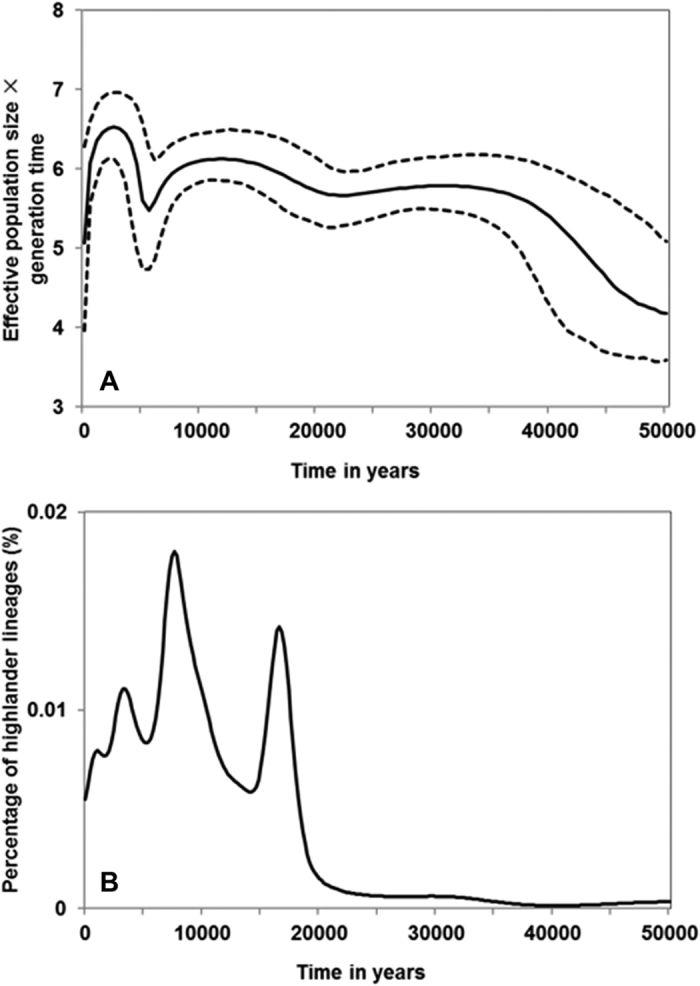
MtDNA Bayesian skyline plot of Tibetan highlanders and probabilistic distribution of the founding lineages. Note: (**A**) The y-axis is the product of maternal effective size and generation time with a base-10 log scale. The x-axis is the time from present in units of years. The solid line is the median estimate and the dashed lines show the 95% highest posterior density limits. (**B**) Probabilistic distribution of founder clusters in Tibetan highlanders across time scanned at 200-year intervals from 0 to 50 kya.

We further inferred the demographic history of 432 highlander sequences of coding regions using Bayesian skyline plot (BSP) (Fig. 1A). Three phases of population expansions emerged. First, the effective population size (*Ne*) of Tibetan highlanders expanded from less than 1,000 to ~20,000 before ~35 kya, assuming 25 years as generation time length. The *Ne* did not change drastically until 20 kya, probably corresponding to the end of LGM. The second expansion occurred between 20 to 10 kya with *Ne* expanding to ~50,000, which was followed by a severe population reduction. Finally, the greatest expansion of highlanders began at ~5 kya, with a ~10-fold *Ne* increase at ~2 kya, but then transit to a recent bottleneck, with *Ne* decreasing to ~4,600.

Further, a founder analysis was conducted by employing highlander sequences as ‘sink’ samples and other East Eurasians as ‘source’ samples (See details in Methods). As a result, the probabilistic distribution of founder clusters showed five peaks (i.e. 30.0, 16.8, 8.0, 3.6 and 1.2 kya), indicating five distinct founding events for colonizing the Tibetan Plateau (Fig. 1B). These founding events coincided well with the three phases of expansions observed in BSP.

In summary, we proposed a three-phase model of colonizing the Tibetan Plateau: pre-LGM phase (before 20 kya), post-LGM Paleolithic phase (20–10 kya) and Neolithic phase (after 10 kya). The partition analysis^17^ showed that the relative proportion of the three phases were 4.68%, 29.31% and 66.01% of founding lineages in Tibetan highlanders, respectively (Table 2).

### Pathogenic mutations in highlander-specific lineages

**Figure 2:**
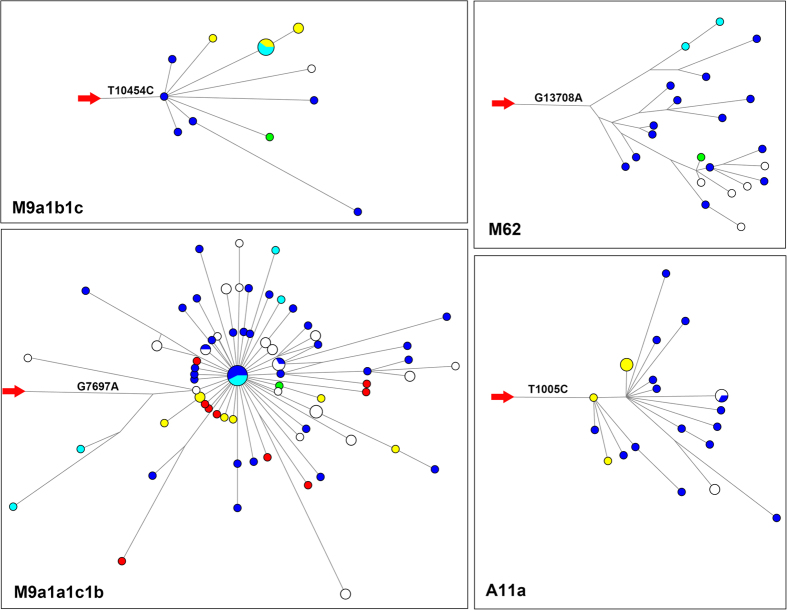
Median-joining networks of 4 highlander-specific lineages carrying pathogenic mutations. Median-joining networks were based on mtDNA coding region sequences corresponding to rCRS positions 577-16023. Tibetans, Lhobas, Dengs, Mongpas and Sherpas were denoted to blue, yellow, green, cyan, and red, respectively. Other East Eurasian sequences were left white. Red arrows indicated the outgroups and the branches where the pathogenic mutations occurred.

Of the 21 major haplogroups, we focused on the highlander-specific lineages, i.e. Haplogroups M62, M13a2, M33b1a1, M9a1a1c1b, M9a1a2, M9a1b1c, A11a, A15c1a and F1d5, of which Haplogroups M62, M9a1a1c1b, M9a1b1c and A11a showed star-like structures in the phylogeny, indicating lineage expansions and possible positive selection in these haplogroups (Figs 2 and S3).

**Figure 3:**
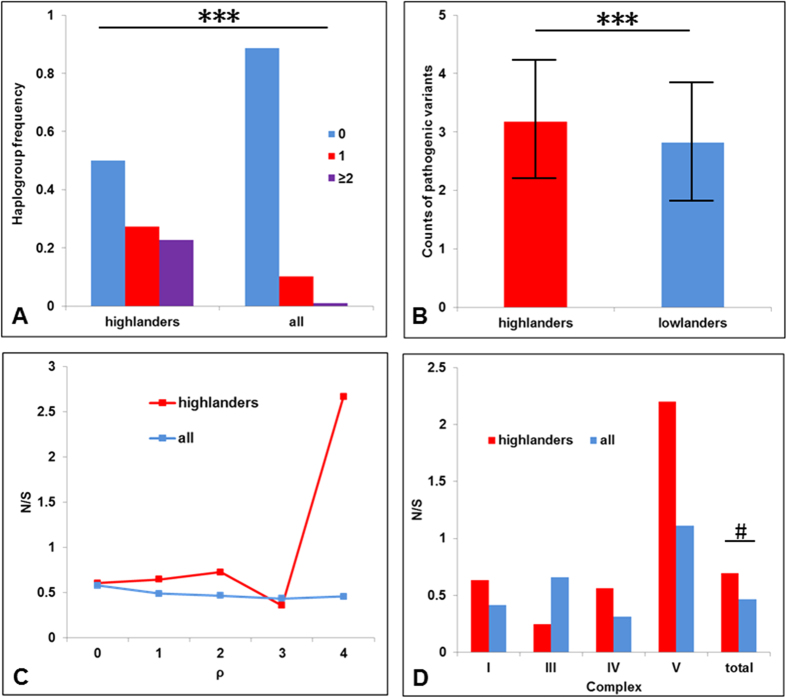
Pathogenic mutations were highly enriched in Tibetan highlanders. (**A**) Pathogenic mutations occurred far more frequently in 22 highlander-specific lineages (five lineages carrying two pathogenic variants and six lineages carrying one) (P = 4.87 × 10^−8^). (**B**) Possible pathogenic mutations in Tibetan highlanders were significantly higher than those in East Asians from 1000 Genomes Projects (P = 1.89 × 10^−4^). (**C**) Nonsynonymous/synonymous ratio (N/S) in haplogroups of highlanders and all the 36,914 sequences respectively. (**D**) N/S comparison between internal branches (1 ≤ ρ ≤ 4) of highlanders and all the 36,914 sequences.

In the phylogeny, we found that there were 22 highlander-specific lineages containing at least 4 haplotypes and 3 highlander samples, which might represent the autochthonous lineages those survived in the highlands (i.e. Haplogroups G3b3a, M13a2, M33b1a1, M62, M7b1a1j, C7a1a2, Z7, M9a1a1c1b, M9a1a2, M9a1b1c, M9a1b1d3, A7, A11a, A15c1a, N11a1, G3b1a, M49a1a1, C4a3b1, D4j1b3, D5a2c2, D5a3a2 and F1c1a1a, see Figure S4). These lineages encompassing 190 individuals accounted for considerable frequencies in 432 random highlanders (43.98%). The defining variants of these highlander-specific lineages may have played a role in the adaptation of all the individuals carrying them. Therefore, we searched the defining variants of the 22 highlander-specific lineages in the database of pathogenic mutations described in MITOMAP (updated on Jan, 8^th^, 2015, with synonymous and HVS mutations removed, 568 mutations in total) to identify mutations that may lead to radical changes in RNA or protein function. As a result, of the 22 haplogroups, 5 lineages harbored 2 pathogenic mutations while 6 lineages carried one potential pathogenic mutation (Table 3 and see networks of 4 major haplogroups in Fig. 2). Among all the 36,914 sequences analyzed, 6,857 haplogroups contained at least 4 haplotypes and 3 samples, of which 701 (10.22%) lineages had one pathogenic mutation while 68 (0.99%) had at least two pathogenic mutations. We found that the pathogenic mutations occurred far more frequently in the highlander-specific lineages (Fig. 3A, Fisher exact test, P = 4.87 × 10^−8^). In addition, even we compared the frequency of pathogenic mutations between highlander-specific lineages and 481 young haplogroups (ρ ≤ 1) with higher ratio of deleterious mutations of above 6,857 haplogroups, the pathogenic mutations still occurred far more frequently in the highlander-specific lineages (Fisher exact test, P = 1.24 × 10^−7^).

The 11 highlander-specific lineages carrying potential pathogenic mutations coalesced in all three phases of colonizing of Tibet, i.e. pre-LGM Time (Haplogroup M62), post-LGM Paleolithic Time (Haplogroup N11a1) and Neolithic Time (Haplogroups A11a, A15c1a, C7a1a2, M9a1a1c1b, M9a1b1c, M7b1a1j, M33b1a1, G3b3a and Z7) (Table S5), suggesting that these function-altering mutations may have played a role in adaptation of highlanders in all three phases of colonization.

Among the 16 pathogenic mutations harbored in the 11 lineages, 6 of them (T3394C, T4216C, T9101C, G7598A and G13708A in 2 lineages) were implicated in LHON, 5 of them (A636G, T1005C, C1192A, T4363C and T10454C) might cause deafness, and 4 of them (T2352C, A3397G, G7697A and A15924G) might lead to several kinds of mitochondrial myopathies (Table 3). These mutations were located almost in all regions of the mitochondrial genome, including the regions coding 3 complexes (Complex I, Complex IV and Complex V), 4 tRNAs and 2 rRNAs. T3394C, which changed the tyrosine (Y) at 30^th^ amino acid of ND1 subunit to a histidine (H), was not only located in highlander-specific lineage G3b3a, but also occurred additional 3 times independently in Deng sample DB033 (Haplogroup M33b1a1), Lhoba sample LB345 (Haplogroup R22), and Haplogroup M9, which was the most frequent haplogroup in Tibetan highlanders. Another mutation A15924G, located on the coding region of tRNA^Thr^, occurred 4 times in highlander-specific lineages (Sherpa sample XEB171 of Haplogroup M9a1a1c1b1a1, Tibetan sample GQ895147 of Haplogroup A11a, Haplogroup M9a1a2a and Sherpa-specific lineage A15c1a). Thus, all the above 16 pathogenic mutations might have certain effect on metabolism and are important candidates for further functional experiments.

### Pathogenic mutations in highlander individuals

The enrichment of pathogenic mutations in highlander individuals could also be examined by site-based association analysis. In the following association analysis, we used 367 East Asians (CHB, CHD, CHS and JPT from 1000 Genome Projects) as controls^18^. Among the 1,809 polymorphic sites in a total of 799 samples (432 highlanders and 367 controls), alleles at 127 sites were detected pathogenic in database from MITOMAP. Among the aforementioned 16 mutations harbored on 11 highlander-specific lineages, T3394C (P = 4.48 × 10^−29^), G7697A (P = 3.72 × 10^−15^), T10454C (P = 4.71 × 10^−6^) and A15924G (P = 4.98 × 10^−5^) reached significant enrichment in highlanders, given adjusted 0.05 level significance (0.05/127 = 3.93 × 10^−4^) using Bonferroni correction. It should be noted that the most significant allele among all 127 sites is T3394C (P = 4.48 × 10^−29^), given the fact that there are 28.24% of T3394C carriers in highlanders, ~17 folds to those in controls (1.63%).

Next, we compared the amount of accumulated pathogenic mutations in highlander sequences with those in East Asians from 1000 Genomes Projects. First, we detect possible deleterious mutations in an individual based on pathogenic mutation database from MITOMAP. Potential pathogenic mutations in highlanders (in average 3.18 ± 1.27) were found significantly higher than those in controls from 1000 Genomes Projects (2.82 ± 1.40) (Mann–Whitney U test, P = 1.89 × 10^−4^) (Fig. 3B). Second, we detected possible tRNA pathogenic candidates according to Kondrashov *et al.*^19^. We found that Tibetan highlanders carried 0.19 (±0.39) tRNA pathogenic mutations in average, significantly more than those in controls (0.13 ± 0.34, Mann–Whitney U test, P = 0.028). Finally, we evaluated the pathogenic effects of nonsynonymous variants using Mutpred scores (ranging from 0 to 1)^20^. Higher scores indicate a greater probability that the amino acid variation might be function-altering and pathogenic. To avoid arbitrary use of cutoffs, we applied Mutpred scores 0.5, 0.6 and 0.7 as pathogenic cutoffs, respectively. Since there are a limited number of mutations with Mutpred scores over 0.8 (only 23 variants in 799 samples), we did not apply cutoffs higher than 0.8. Using all the three cutoffs, highlanders contained significantly more pathogenic nonsynonymous mutations than controls, respectively (1.73 ± 1.23 vs. 1.20 ± 0.96 for 0.5 cutoff, P = 2.69 × 10^−10^; 1.00 ± 0.90 vs. 0.51 ± 0.72 for 0.6 cutoff, P = 1.80 × 10^−17^; 0.36 ± 0.52 vs. 0.15 ± 0.38 for 0.7 cutoff, P = 2.56 × 10^−11^).

In addition, we also employed two East Asian populations as lowland controls to conduct the same analyses in this section, one of which included 444 Han Chinese from Rugao, Jiangsu Province^21^, another included 550 Taiwan aborigines^22^. Generally, the results employing the two single populations were consistent to those analyses using 367 East Asians as controls (data not shown). In short, we inferred that pathogenic mutations were enriched in Tibetan highlanders.

### Ratio of nonsynonymous and synonymous mutations

We counted nonsynonymous (N) and synonymous mutations (S) in the highlander-specific lineages and the haplogroups of all the 36,914 sequences in this study. The coalescence time of each haplogroup was estimated based on ρ-statistic value for further grouping to calculate corresponding ratio of nonsynonymous and synonymous mutation (N/S). We showed earlier that the major expansion of Tibetan highlanders and the majority of immigrations to the Tibetan Plateau occurred in the past several thousand years. Thus, we focused on the highlander-specific lineages with recent coalescence (ρ ≤ 4).

In Fig. 3C, N/S of lineages of each ρ unit was calculated. For all the 36,914 sequences, the N/S declined with the increase of the age of lineages. In detail, the N/S of the external lineages (ρ = 0, N/S = 0.58) was significantly higher than that of internal lineages within 10 kya (1 ≤ ρ ≤ 4, N/S = 0.47, P = 1.46 × 10^−17^). This observation suggested that mtDNA was under purifying selection against deleterious nonsynonymous mutations, coinciding with previous study^23^,^24^,^25^. As contrast, the N/S of the highlander-specific haplogroups showed an opposite trend, i.e., increasing with the ages of lineages (Fig. 3C). We found that the N/S of the external lineages (ρ = 0, N/S = 0.61) was lower than that of the internal lineages (1 ≤ ρ ≤ 4, N/S = 0.69), although not statistically significant. This observation suggested that some of the nonsynonymous mutations might have been favorably preserved in the highlander-specific haplogroups, which was a signal of positive selection.

Interestingly, the N/S of the external branches of the highlander-specific lineages (ρ = 0, N/S = 0.61) was very close to that of all the 36,914 sequences (ρ = 0, N/S = 0.58) (Fig. 3C). However, we found that the N/S in the internal branches of the highlander-specific haplogroups (1 ≤ ρ ≤ 4, N/S = 0.69) was nearly significant higher than that of all the 36,914 sequences (1 ≤ ρ ≤ 4, N/S = 0.47, P = 0.058) (Fig. 3D, Table S6). In detail, we grouped these variants according to four complexes and found that the N/S of 3 complexes of the highlander-specific lineages was higher than that of all the 36,914 sequences respectively except for Complex III (Cytochrome bc1 complex), suggesting a mitochondrial genome-wide trend.

### Demographic simulations

Considering that N/S might be elevated by the recent bottleneck in Tibetan highlanders but not positive selection, we further conducted forward demographic simulations to rule out such possibility.

We selected 432 random Tibetan highlanders from the current study as a representative of highlanders and the 367 random East Asian samples from 1000 Genomes Projects as a representative of East Asian lowlanders. Time of the mutations was estimated based on the coalescence time of corresponding haplogroups in the mtDNA phylogeny of 36,914 sequences. As a result, the N/S of the external lineages of East Asian lowlanders (ρ = 0, N/S = 0.54) was significantly higher than that of the internal lineages within 10 kya (1 ≤ ρ ≤ 3, N/S = 0.28, P = 0.0005), while the N/S of the external lineages of Tibetan highlanders (ρ = 0, N/S = 0.57) was lower than that of internal lineages (1 ≤ ρ ≤ 3, N/S = 0.64), although not significantly. This result was consistent to the previous observation in the last section, indicating that the mtDNA of lowlanders was under purifying selection against deleterious nonsynonymous mutations, while nonsynonymous mutations were favorably preserved in highlander lineages.

Based on the BSP plot of 367 East Asians and 432 random Tibetan highlanders, we reconstructed the evolutionary history of lowlanders and highlanders (See Fig. 1 and Zheng *et al.*)^18^. According to the demographic models from BSPs (Check Figure S5 for details), we employed SFS_CODE to conduct forward simulations with the effect of purifying selection^26^. The detail settings of SFS_CODE were referred in Material and Methods. The results of simulations showed that N/S of the external lineages (ρ = 0) was significantly lower than that of the internal lineages within 10 kya (1 ≤ ρ ≤ 3) under purifying selection in both East Asian lowlanders and Tibetan highlanders (Table 4), coinciding with our observation on East Asians but different with highlanders. This result indicated that it still was of little chance to observe higher N/S in internal lineages for Tibetan highlanders when bottleneck effect was considered, inferring that the higher N/S in the internal lineages violated the simple models with purifying selection and thus positive selection might help to preserve the internal nonsynonymous mutations. In short, the highlander lineages carried more nonsynonymous and function-altering mutations in the internal branches, indicating a signal of positive selection.

Considering all above, we hypothesize that the enriched function-altering and pathogenic mutations in Tibetan highlanders may have played a role in adaptation of hypoxia environment. Nevertheless, there is no direct evidence relating the pathogenic mutations to hypoxia adaptation and other explanations to our observations are possible. Further studies, especially functional experiments are necessary to reveal the mechanism how pathogenic mutations contribute to hypoxia adaptation.

## Discussion

In the phylogeny, the 21 major haplogroups of Tibetan highlanders could generally be divided to two types. Some haplogroups are highlander-specific lineages, such as Haplogroups M62, M13a2, M9a1a1c1b, M9a1b1c, M9a1a2 and A11a, indicating that the ancestors of these lineages migrated to the highlands (Figure S4). Other haplogroups derive considerable immediate non-highlander lineages, such as Haplogroups F1g and D5a2c, implying that these lineages split to lowlands and highlands respectively. Both types of the haplogroups above indicated founding events for colonizing the Tibetan Plateau, which were used as criteria for identification of founder haplotypes assuming sufficient sampling in outgroups (See Methods, and^17^). Generally, the most recent common ancestor of highlanders and other East Eurasians would be considered as a founder. Founder analysis based on mtDNA hyper-variation regions (HVR) was employed in previous studies to reconstruct the migration history of modern humans^17^,^27^. With the aggregation and accumulation of mtDNA complete sequences, we tried to conduct founder analysis on whole mtDNA sequences for their high phylogenic resolution. As a result, we proposed a three-phase model of colonizing the Tibetan Plateau, i.e. pre-LGM Time (30 kya, 4.68%), post-LGM Paleolithic Time (16.8 kya, 29.31%) and Neolithic Time (after 8 kya, 66.01% in total). Lines of archeological evidence revealed that modern humans colonized the Tibetan Plateau before LGM^2^ and thrived with the advent of agriculture^28^. Previous genetic studies on mtDNA and Y chromosomes also showed that the course of colonization was throughout the Paleolithic and Neolithic Times^29^,^30^,^31^,^32^,^33^.

Among the major haplogroups of the highlands, 206 individuals (47.69%) from 13 haplogroups (Haplogroups G3b1, M13a2, C4a3b, D4j1a1, D4j1b, D5a2c, M9a1a1c1b, M9a1a2, M9a1b1c, M9a1b1d, A11a, A6 and F1d5) were present in at least 3 ethnic groups (i.e. Tibetans, Lhobas, Monpas, Dengs and Sherpas) (Table 1), indicating a common origin of ethnic groups of the Tibetan Plateau, which was consistent to the previous observation using MDS plots. Three of these haplogroups (Haplogroups M13a2, D4j1b and A6, 4.86% of the 432 highlanders) were coalesced at the post-LGM Paleolithic Time while the remaining 10 haplogroups (95.14%) coalesced in Neolithic Time. Thus, we hypothesized that the ethnic groups of Tibetan Plateau separated from the end of LGM, and mainly in Neolithic Time. Founder analysis of Sherpas, Dengs, Lhobas and Monpas also had reconciled results (Figure S6). In legends of the Tibetan Plateau, there were four great tribes, i.e. Sé (se), Mu (rmu), Dong (ldong), and Tong (stong) in Tibetans, which further divaricated to other ethnic groups such as Lhoba, Monpa and Sherpa, coinciding with our results^34^.

Radical mtDNA variations would have considerable effect on OXPHOS and cause mitochondrial diseases, especially in the tissues highly dependent on oxidative metabolism, like retinas, ears, skeleton muscles, hearts and etc.^13^,^14^. In this study, we found that 16 pathogenic mutations were harbored in 11 highlander-specific lineages, which might have certain effect on OXPHOS and further impact on metabolism. These 16 mutations might have pathogenic effect on lowland people for impairment on OXPHOS function^35^. However, some studies also showed that these mutations occurred in healthy subjects, indicating that these pathogenic mutations might not result in diseases under certain conditions (e.g. autosome backgrounds and environments)^36^,^37^. In recent years, genomic studies have revealed numerous genes contributing to hypoxia adaptation for highlanders, indicating the complex mechanism underlying the adaptation, including interaction of multiple genes^38^,^39^, environment effect^28^ and even introgression from archaic humans^40^.

A recent paper^41^ showed that hypoxia was protective against mitochondrial toxicity in cultured cells and zebrafish models with respiratory chain deficiency. In addition, chronic hypoxia leaded to a marked improvement in survival and disease biomarkers in a genetic mouse model of Leigh syndrome. The normal concentration of oxygen was toxic to patients with respiratory chain deficiency. However, hypoxia might improve the symptoms by triggering innate adaptive programs (such as HIF pathway) and by simultaneously limiting the substrate for oxygen toxicity. The results indicated a natural match for hypoxia environments in Tibetan highlands and mild mitochondrial dysfunction caused by mtDNA deleterious mutation, which provide functional evidence for our work.

HIF pathway, involving two genes (*EGLN1* and *EPAS1*) of significant signals of positive selection, was under extensive focus. Mitochondria could generate ROS and have further effect on HIF pathway. Thus, we hypothesize that function-altering and pathogenic mutations of mtDNA may affect OXPHOS function to generate and accumulate ROS in mitochondria, subsequently inhibit PHD2 or stabilize HIF, and result in contribution in hypoxia adaptation. Nevertheless, there is no direct evidence relating the pathogenic mutations to hypoxia adaptation. Further studies, especially functional experiments are necessary to reveal the detailed mechanism.

## Methods

### Population and samples

Four ethnic groups (in total 285 samples) residing in the Tibetan Plateau were collected in this study, including 86 Tibetans from Tingri County in Shigatse Prefecture, 91 Lhobas from Mainling and Gongbo’gyamda Counties in Nyingtri Prefecture, 91 Dengs from Zayü County in Nyingrtri Prefecture, and 17 Monpas from Mainling and Gongbo’gyamda Counties in Nyingtri Prefecture. All these samples were maternally unrelated. This research was approved by both the Human Ethics Committees of Tibet University for Nationalities and School of Life Sciences of Fudan University, and was carried out in accordance with the approved guidelines. Informed consent was obtained from all subjects collected in the current study. The sequencing and variant calling methods for these samples were according to those described previously^15^,^21^. Thirty-three Tibetan mtDNA sequences were obtained from fastq files of Yi *et al.*^9^, of which missing sites were further imputed based on the phylogeny. We also collected all 223 Tibetan highlander mtDNA sequences available in the literature^4^,^11^,^15^,^30^,^31^,^42^. Eight additional Tibetan sequences generated in our lab were also included for phylogenetic analysis as Tibetan highlanders. In total, 549 mitochondrial sequences of Tibetan highlanders were analyzed in this study, of which 432 sequences composed a random highlander dataset (See Table S1). Finally, 293 mitochondrial genomes were deposited in Genbank (KT725860-KT726152). Variations to rSRS of 33 Tibetan mtDNA from fastq files of Yi *et al.* were attached as supplemental data (Table S2). A total of 36,914 world-wide mtDNA sequences were analyzed in this study, which included all available sequences from Phylotree database^16^,^43^ and unpublished data in our lab.

### Data analysis

Complete mtDNA sequences were assigned to haplogroups generally according to PhyloTree Build 16^16^,^43^. The complete median-joining network of Tibetan highlanders (Figure S3) was reconstructed by Network v4.6^44^. Considering the network and PhyloTree Build 16, we generated the final phylogeny (Figure S4). Haplogroups Z3b, Z7, A7 and D5a2 were modified according to updated sequences compared to PhyloTree Build 16. Haplogroups A15c1a, G3b3, G3b1a, G2a1a, M33b1a, D4j1b3, D4h1c1a, D5a3a2, M49a1a, M7b1a1j, M9a1b1d, F1c1a2, F1d1a and F1d5 were newly named in the current study.

Then, the phylogeny of Tibetan highlanders was used to examine the assumption of a molecular clock under the HKY + G mutation model. The null hypothesis of a molecular clock cannot be rejected (P = 1.00) using PAML package v4.4^45^. According to the phylogeny, the coalescence time of lineages of interest was estimated using ρ statistic-based method and maximum likelihood method implemented with all available sequences in these lineages. For ρ statistic-based method, standard deviation was calculated following Saillard *et al.*^46^. Then the time to TMRCA of each lineage was estimated using Soares rate for complete mtDNA sequences^25^.

Bayesian skyline plots (BSP) for the 7 populations of the Tibetan Plateau and 432 random highlander sequences were generated by BEAST 1.8.1^47^ and Tracer 1.5.1 using mtDNA coding regions (576–16023). Each MCMC sample was based on a run of 100 million generations sampled every 10,000 steps with the first 10 million generations regarded as burn-in. We used the HKY+G model of nucleotide substitution without partitioning the coding region. A strict clock was used and prior substitution rate was set 1.691 × 10^−8^ subs/site/year^48^.

*Φ*_ST_ distances between populations were calculated in Arlequin 3.11^49^ using coding regions, and plotted in PAST 1.85^50^ with a non-metric multidimensional scaling method (MDS), showing that highlander populations were clustered together and close to other East Asian populations. Tajima’s D as well as Fu & Li’s D* and F* tests were performed using DnaSP v5.10^51^.

To clarify the colonizing history of the Tibetan Plateau, we conducted a founder analysis, using all 549 highlander sequences as ‘sink’ samples and other East Eurasians as ‘source’ samples in the phylogeny. We used an *f0* criterion to identify founders^17^, for the majority of highland founding events occurred within several thousand years. We identified candidate founders by searching for 1) identical haplotypes in Tibetan highlanders and other East Eurasians, and 2) reconstructed ancestor haplotype with both derivatives of highlanders and other East Eurasians, which are either a) unsampled haplotypes with both immediate highlander and non-highlander derivatives, or b) haplotypes sampled only in Tibetan highlanders and whose immediate derivatives include at least one other East Eurasian, or c) haplotypes sampled only in non-highlanders and whose immediate derivatives include at least one highlander. Generally, the most recent common ancestor of highlander and other East Eurasians would be considered as a founder.

In the phylogeny, 21 haplogroups were considered as major haplogroups of founding the Tibetan Plateau since these haplogroups accounted for considerably high frequencies in 432 random highlanders or appeared in at least three ethnic groups of Tibetan highlanders (Figures S3 and S4 and Table 1). Most of these major haplogroups are derived from a single founder respectively except that Haplogroup D4j1a1 might be derived from several but phylogenetically near founders.

We estimated the migration times of all candidate founders using the ρ statistic^52^ and a complete mutation rate^25^. Here, the ρ statistic represented the average number of mutations between the founder haplotype and every highlander sequence. Then, the sample size of each founder was adjusted according to 432 random Tibetan highlanders. We scanned the distribution of founder ages for Tibetan highlanders defining equally spaced 200-year intervals for each migration from 0 to 50 kya. A second partitioning analysis was conducted using defined migration time based on the previous scan. This second analysis allowed us to fractionate the extant highlander lineages into each migration and estimate the percentage of lineages in each migration event. Non-highlander samples in Haplogroups A11a, M13b2, M67, M62, N11a1, M9a1a1c1b, M9a1a2 and M9a1b1c were considered to occur due to back migrations. Ruling out the back migrations, these haplogroups were still analyzed as autochthonous in the Tibetan Plateau and highlander-specific.

In the phylogeny, we found that there were 22 highlander-specific lineages containing at least 4 haplotypes and 3 highlander samples, which might represent the lineages those survived in the highlands. We searched the defining variants of the 22 highlander-specific lineages in the database of pathogenic mutations described in MITOMAP (568 pathogenic mutations in total, updated on Jan, 8^th^, 2015, with synonymous and HVS mutations removed)^35^ to identify mutations that lead to radical changes in RNA or protein function. Among all the 36,914 sequences analyzed, 6,857 haplogroups contained at least 4 haplotypes and 3 samples, of which defining variants were also detected for pathogenic mutations. Next, we compared the frequencies of highlander-specific lineages carrying different numbers of pathogenic mutations to those of the haplogroups of all the 36,914 sequences.

In addition, site-based association analysis was conducted using 432 random Tibetan highlanders as cases and 367 East Asians from 1000 Genomes Projects as controls^18^. Accumulated possible pathogenic mutations in Tibetan highlanders were also compared to those in 367 East Asians from 1000 Genomes Projects based on database of pathogenic mutations described in MITOMAP^35^, tRNA pathogenicity^19^ and Mutpred scores^20^, respectively. Conservation index of mutations (CI) was consulted to MitoTool^53^. Nonsynonymous/synonymous mutation ratio in highlander-specific lineages was compared to that in the contemporary haplogroups of all the 36,914 sequences according to the coalescence time estimated by the ρ statistic-based method. The nonsynonymous and synonymous mutations were counted based on the branches of the phylogeny and the mutations on a branch were only counted once.

According to the demographic models from BSPs, we employed SFS_CODE^26^ to conduct forward simulations with the effect of purifying selection. Generally, we simulated the protein coding regions with length of 11367 base pairs and sampled 300 individuals for each run. Each model was simulated 100 times for East Asians and 1000 times for Tibetan highlanders, respectively. When evaluating models with different strengths of purifying selection, we assumed that selection coefficients for the nonsynonymous sites were drawn from a gamma distribution. To access the distribution of selection coefficients for mutations in 367 East Asians, we employed the strategy referred in Tamuri *et al.*^54^ and found that ~80% of the nonsynonymous mutations were strongly deleterious with population scaled coefficient less than −10 (data not shown). Thus, we set the selection coefficients in SFS_CODE as −10, −20 and −50 respectively for each demographic model and 80% of the nonsynonymous mutations under the purifying selection. In addition, considering the lethal effect for mitochondrial mutations, we set the non-lethality parameter in SFS_CODE as 0.9.

## Additional Information

**How to cite this article**: Kang, L. *et al.* MtDNA analysis reveals enriched pathogenic mutations in Tibetan highlanders. *Sci. Rep.* **6**, 31083; doi: 10.1038/srep31083 (2016).

## Supplementary Material

Supplementary Information

## Figures and Tables

**Table 1:** Coalescence time and frequencies of 21 major haplogroups of Tibetan highlanders.

	Tibetans (n = 157)	Dengs (n = 91)	Lhobas (n = 91)	Sherpas (n = 76)	Monpas (n = 17)	Highlanders (n = 432)	Soares Complete Rate T(kya) ± SD(kya)		
							n^a^	ρ-based Method	ML Method
G3b1	3	2	0	0	1	6	9	8.49 ± 1.79	10.46 ± 2.68
M13a2	4	0	1	2	0	7	13	14.66 ± 4.47	10.68 ± 3.10
M33b1a1	0	8	0	0	0	8	8	1.29 ± 0.64	1.54 ± 1.13
M62	3	1	0	0	0	4	22	26.41 ± 4.46	24.24 ± 5.09
C4a3b	1	1	2	14	0	18	21	9.31 ± 2.55	9.05 ± 2.37
Z3b	0	8	0	0	0	8	10	5.22 ± 2.37	7.54 ± 10.68
D4h1c1a1	0	8	0	0	0	8	8	7.22 ± 3.11	5.94 ± 6.68
D4j1a1	4	1	3	1	0	9	40	6.95 ± 1.50	5.94 ± 1.75
D4j1b	3	0	1	0	1	5	9	11.22 ± 2.57	11.33 ± 3.41
D5a2c	3	2	11	0	2	18	27	7.51 ± 0.98	10.46 ± 1.75
M9a1a1c1b	33	1	4	11	3	52	88	10.95 ± 3.60	9.48 ± 4.35
M9a1a2	4	3	5	3	0	15	23	7.89 ± 2.65	8.51 ± 9.70
M9a1b1c	3	1	5	0	3	12	16	7.39 ± 2.13	6.79 ± 1.85
M9a1b1d	3	31	0	0	1	35	44	7.40 ± 2.77	7.54 ± 1.85
A11a	9	0	4	2	0	15	24	8.45 ± 2.23	7.54 ± 2.78
A15c1a	0	0	0	14	0	14	14	1.66 ± 0.88	1.33 ± 1.13
A6	1	1	4	3	0	9	14	12.55 ± 2.20	14.54 ± 2.58
F1c1a2a1	0	0	7	0	0	7	7	5.22 ± 3.18	5.35 ± 4.00
F1d1a	1	0	7	0	0	8	11	4.51 ± 1.14	4.04 ± 1.44
F1d5	1	1	3	0	0	5	5	6.82 ± 3.15	8.50 ± 5.20
F1g	7	0	3	0	0	10	20	9.22 ± 1.12	9.37 ± 1.33

^a^Time estimates were based on all available sequences belonging to the haplogroup.

**Table 2:** Percentage of founding lineages of Tibetan highlanders colonizing the Tibetan Plateau assuming specific migration times.

Migration Time (kya)	Percentage of lineages of Tibetan highlanders	S.E.
1.2	9.90	1.28
3.6	17.05	1.90
8.0	39.06	2.00
16.8	29.31	1.43
30.0	4.68	0.63

Note: Migration times were obtained from probabilistic distribution of founder clusters (See Fig. 1B).

**Table 3:** Potential pathogenic mutations located in the highlander-specific lineages.

highlander-specific haplogroup	Potential pathogenic mutation^a^	Gene	CI	Mutpred Score	Potential disease^b^
G3b3a	T3394C*	ND1(Y30H)	0.942	0.783	LHON/NIDDM/CPT deficiency
M33b1a1	A636G	tRNA^Phe^	0.673	N.A.	DEAF
	T9101C	ATP6(I192T)	0.346	0.568	LHON
M62	G13708A	ND5(A458T)	0.365	0.409	LHON/Increased MS risk/higher frequency in PD-ADS
M7b1a1j	C1192A	12s rRNA	0.827	N.A.	DEAF-associated
	A3397G	ND1(M31V)	1.000	0.723	PD, AD/Possibly LVNC-cardiomyopathy associated
C7a1a2	G7598A	COX2(A5T)	0.865	0.342	Possible LHON helper
	G13708A	ND5(A458T)	0.365	0.409	LHON/Increased MS risk/higher frequency in PD-ADS
Z7	T2352C	16s rRNA	0.058	N.A.	Possibly LVNC-associated
	T4363C	tRNA^Gln^	0.750	N.A.	Possibly associated with DEAF + RP + developmental delay/hypertension
M9a1a1c1b	G7697A*	COX2(V38I)	0.981	0.646	Possible HCM susceptibility
M9a1b1c	T10454C*	tRNA^Arg^	0.692	N.A.	DEAF helper mutation
A11a	T1005C	12s rRNA	0.250	N.A.	DEAF
A15c1a	T4216C	ND1(Y304H)	0.712	0.611	LHON/Insulin Resistance
A15924G*	tRNA^Thr^	0.865	N.A.	LIMM	
N11a1	A12634G	ND5(I100V)	1.000	0.381	Thyroid Cancer Cell Line

^a*^Also significant in site-based association analysis.

^b^consulted to MITOMAP database. LHON: Leber’s hereditary optic neuropathy; NIDDM: non-insulin-dependent diabetes mellitus; CPT: carnitine palmitoyl transferase; DEAF: deafness; MS: multiple sclerosis; PD: Parkinson’s disease; AD: Alzheimer’s Dementia; LVNC: left ventricular non-compaction cardiomyopathy; RP: retinitis pigmentosa; HCM: hypertrophic cardiomyopathy; LIMM: lethal infantile mitochondrial myopathy.

**Table 4:** Probabilities of higher N/S of internal branches in simulated models.

selection coefficient^a^	P of East Asian Model^b^			P of Tibetan highlander Model^c^		
	1	2	3	1	2	3
−10	0.04	0.00	0.00	0.066	0.052	0.050
−20	0.00	0.00	0.00	0.016	0.017	0.006
−50	0.00	0.00	0.00	0.035	0.031	0.024

^a^Population scaled selection coefficient for SFS_CODE.

^b^Probabilities of observing higher N/S of internal branches (1 ≤ ρ ≤ 3) in East Asian models.

^c^Probabilities of observing higher ratio of N/S of internal branches (1 ≤ ρ ≤ 3) and external branches (ρ = 0) than the observing value 1.129 in Tibetan highlanders.
