# Supplementary material for: MtDNA analysis reveals enriched pathogenic mutations in Tibetan highlanders
Source: Sci Rep. 2016 Aug 8;6:31083. doi: 10.1038/srep31083 (PMC4976311; doi:10.1038/srep31083)
Supplement: Supplementary Information [file srep31083-s1.pdf]

# MtDNA analysis reveals enriched pathogenic mutations in Tibetan highlanders

Longli Kang[1]†*, Hong-Xiang Zheng[2]†, Menghan Zhang[2], Shi Yan[2], Lei Li[2], Lijun Liu[1], Kai Liu[3], Kang Hu[1], Feng Chen[1], Lifeng Ma[1], Zhendong Qin[2], Yi Wang[2], Xiaofeng Wang[2], Li Jin[2]*

[1]Key Laboratory for Molecular Genetic Mechanisms and Intervention Research on High Altitude Disease of Tibet Autonomous Region; Key Laboratory of High Altitude Environment and Gene Related to Disease of Tibet Ministry of Education, School of Medicine, Xizang University for Nationalities, Xianyang, China

[2]Ministry of Education Key Laboratory of Contemporary Anthropology and Center for Evolutionary Biology, School of Life Sciences and Institutes of Biomedical Sciences, Fudan University, Shanghai, China

[3]Tibet Occupational College of Technology, Lhasa, Tibet, China

†These authors contributed equally to this work

*Correspondence to:

Longli Kang (klonglister@gmail.com) and Li Jin (lijin@fudan.edu.cn)

## Supplementary information

Figure S1. Non-metric multidimensional scaling of mtDNA $\Phi$st distance

Figure S2. mtDNA Bayesian skyline plots of 7 populations from the Tibetan Plateau

Figure S3. Median-joining network of 432 mtDNA coding region sequences of Tibetan highlanders

Figure S4. Detailed mtDNA phylogeny of selected haplogroups of Tibetan highlanders

Figure S5. Models constructed for simulations

Figure S6. Probabilistic distribution of founder clusters in Sherpas, Dengs, Lhobas and Monpas

Table S1. Sample information of 549 Tibetan highlanders

Table S2. 33 Tibetan mtDNA haplotypes compared to rSRS from fastq files of Yi et al.

Table S3. Haplogroup frequencies in 7 populations from the Tibetan Plateau

Table S4. Genetic diversities of 7 populations of the Tibetan Plateau

Table S5. Coalescence time of 22 highlander-specific lineages

Table S6. Comparison on the NS/S between the internal lineages of Tibetan highlanders ($1 \leq \rho \leq 4$) and the contemporary haplogroups of all the 36,914 sequences in all protein-coding genes and four complexes respectively

**Figure S1. Non-metric multidimensional scaling of mtDNA *Φ*st distance**

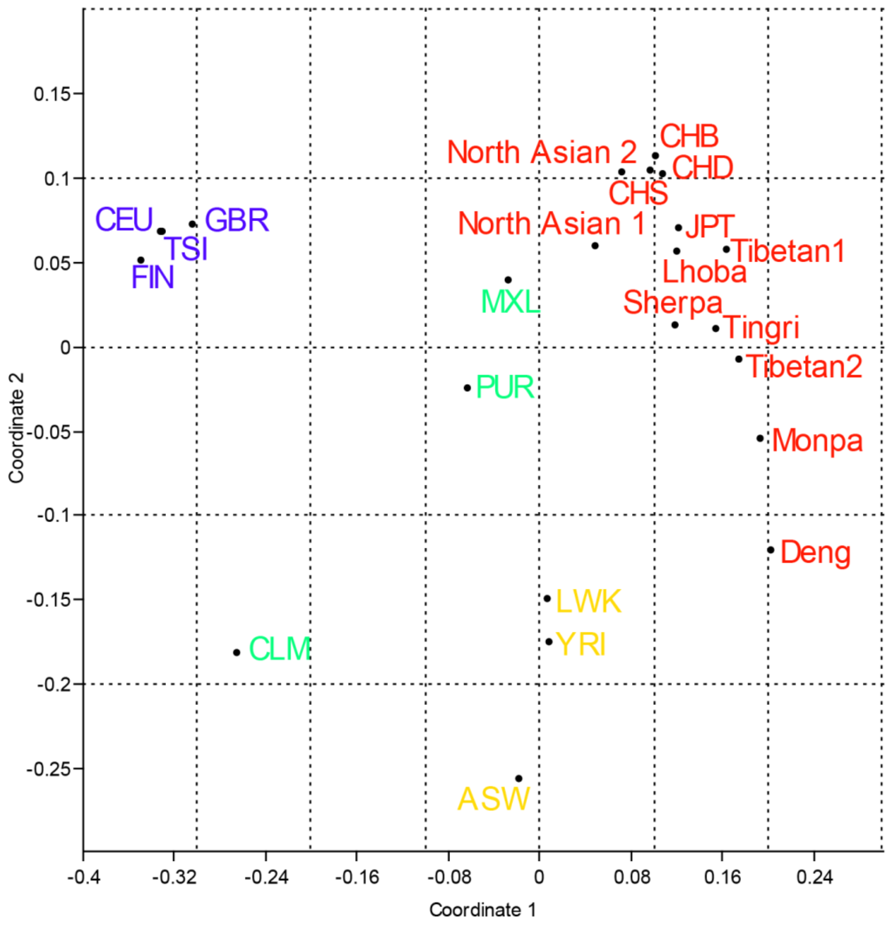


Populations including Tingri Tibetan, Lhoba, Monpa, Deng and Tibetan2 were from this study. Previous reported data were CHB, CHD, CHS, JPT [1], YRI, LWK, ASW, FIN, CEU, TSI, GBR, MXL, PUR, CLM [2], Tibetan1 [3], North Asian population 1 [4], North Asian population 2 [5] and Sherpa [6]. Orange, Africans; Red, East Asians; Green, Americas; Blue, Europeans.

## Figure S2. mtDNA Bayesian skyline plots of 7 populations from the Tibetan Plateau

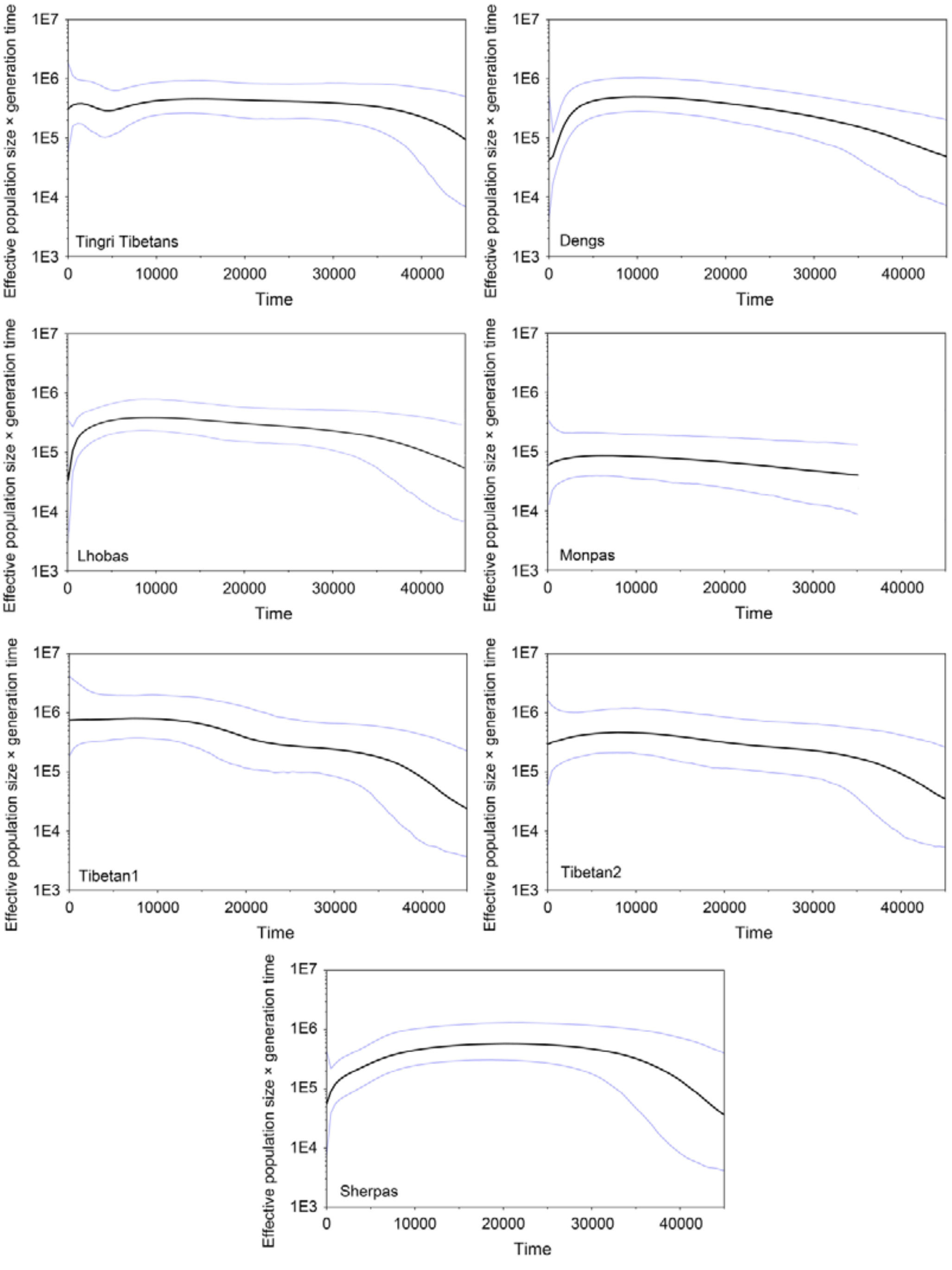


The y-axis is the product of maternal effective size and generation time. The x-axis is the time from present in units of years. The thick solid line is the median estimate and the thin lines (blue) show the 95% highest posterior density limits. Detailed settings refer to Material and Methods.

**Figure S3. Median-joining network of 432 mtDNA coding region sequences of Tibetan highlanders**

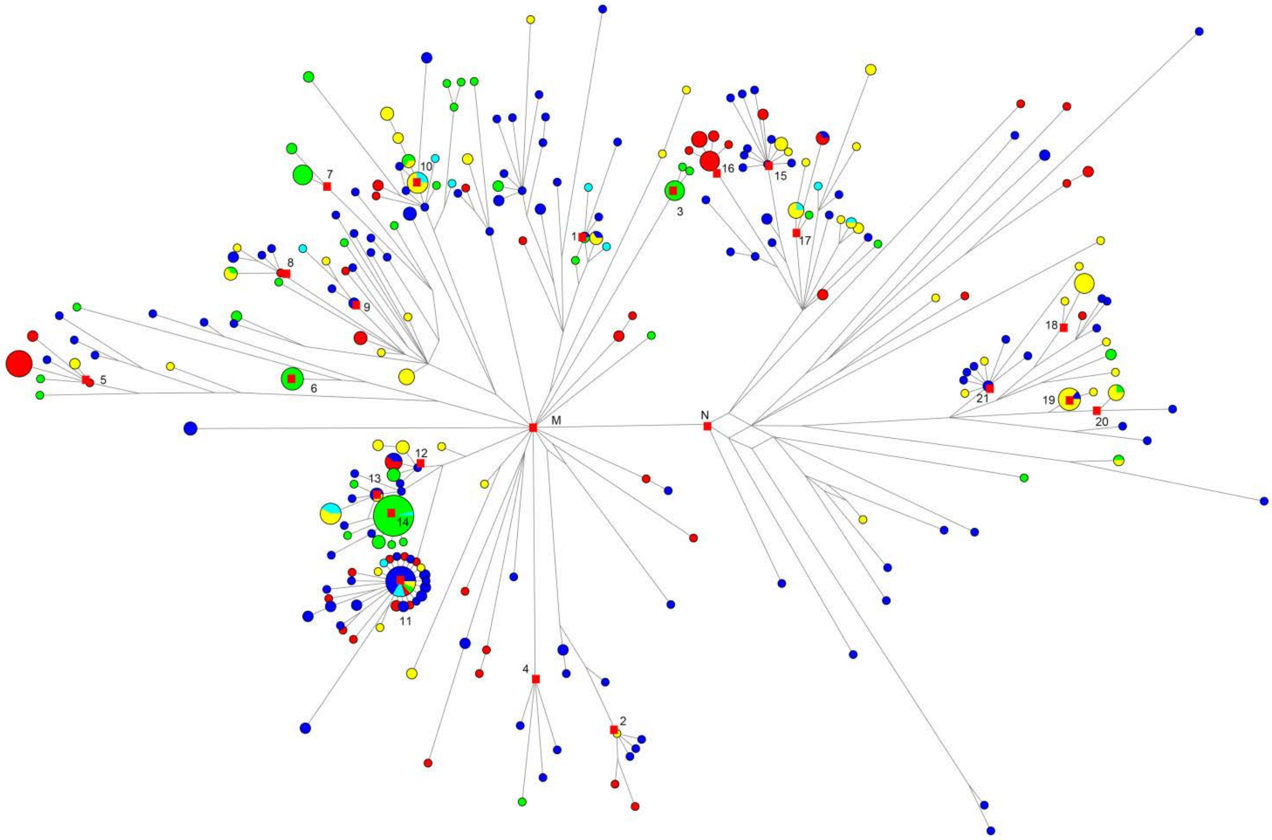


Median-joining network of 432 mtDNA coding region sequences corresponding to rCRS positions 577-16023. Branch length is proportional to steps of mutations. Red squares and numbers denote 21 major lineages of Tibetan highlanders. 1, G3b1; 2, M13a2; 3, M33b1a1; 4, M62; 5, C4a3b; 6, Z3b; 7, D4h1c1a1; 8, D4j1a1; 9, D4j1b; 10, D5a2c; 11, M9a1a1c1b; 12, M9a1a2; 13, M9a1b1c; 14, M9a1b1d; 15, A11a; 16, A15c1a; 17, A6; 18, F1c1a2a1; 19, F1d1a; 20, F1d5; 21, F1g. Tibetans, Lhobas, Dengs, Mongpas and Sherpas were denoted in blue, yellow, green, cyan, and red, respectively. Non-highlander sequences were left white.

## Figure S4. Detailed mtDNA phylogeny of selected haplogroups of Tibetan highlanders

Every mutation was denoted to each haplogroup. Insertions were represented as ‘.’. Deletions were added ‘d’ in the end. Blue denotes HVS variants and non-coding mutations in coding regions. Red means synonymous mutations while Black means nonsynonymous mutations. Green points out tRNA and rRNA variants. Tibetans, Lhobas, Dengs, Mongpas and Sherpas were denoted to blue, yellow, green, cyan, and red, respectively. Non-highlander sequences were left white.

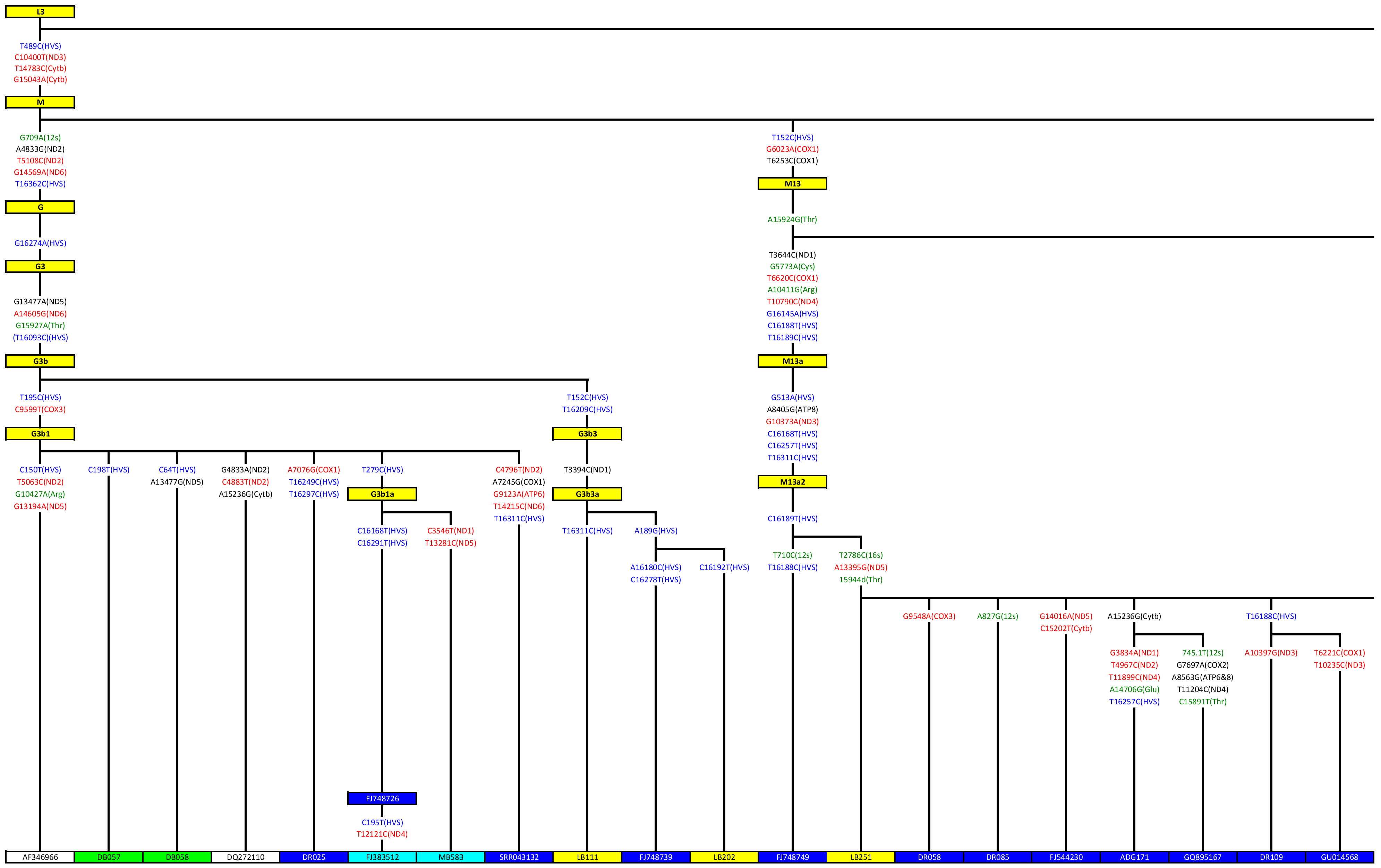
L3
T489C(HVS)
C10400T(ND3)
T14783C(Cytb)
G15043A(Cytb)
M
G709A(12s)
A4833G(ND2)
T5108C(ND2)
G14569A(ND6)
T16362C(HVS)
G
G16274A(HVS)
G3
G13477A(ND5)
A14605G(ND6)
G15927A(Thr)
(T16093C)(HVS)
G3b
T195C(HVS)
C9599T(COX3)
G3b1
C150T(HVS)
T5063C(ND2)
G10427A(Arg)
G13194A(ND5)
C198T(HVS)
C64T(HVS)
A13477G(ND5)
G4833A(ND2)
C4883T(ND2)
A15236G(Cytb)
A7076G(COX1)
T16249C(HVS)
T16297C(HVS)
T279C(HVS)
G3b1a
C16168T(HVS)
C16291T(HVS)
FJ748726
C195T(HVS)
T12121C(ND4)
C3546T(ND1)
T13281C(ND5)
C4796T(ND2)
A7245G(COX1)
G9123A(ATP6)
T14215C(ND6)
T16311C(HVS)
T152C(HVS)
T16209C(HVS)
G3b3
T3394C(ND1)
G3b3a
T16311C(HVS)
A189G(HVS)
A16180C(HVS)
C16278T(HVS)
C16192T(HVS)
T152C(HVS)
G6023A(COX1)
T6253C(COX1)
M13
A15924G(Thr)
T3644C(ND1)
G5773A(Cys)
T6620C(COX1)
A10411G(Arg)
T10790C(ND4)
G16145A(HVS)
C16188T(HVS)
T16189C(HVS)
M13a
G513A(HVS)
A8405G(ATP8)
G10373A(ND3)
C16168T(HVS)
C16257T(HVS)
T16311C(HVS)
M13a2
C16189T(HVS)
T710C(12s)
T16188C(HVS)
T2786C(16s)
A13395G(ND5)
15944d(Thr)
G9548A(COX3)
A827G(12s)
G14016A(ND5)
C15202T(Cytb)
A15236G(Cytb)
G3834A(ND1)
T4967C(ND2)
T11899C(ND4)
A14706G(Glu)
T16257C(HVS)
745.1T(12s)
G7697A(COX2)
A8563G(ATP6&8)
T11204C(ND4)
C15891T(Thr)
T16188C(HVS)
A10397G(ND3)
T6221C(COX1)
T10235C(ND3)
AF346966
DB057
DB058
DQ272110
DR025
FJ383512
MB583
SRR043132
LB111
FJ748739
LB202
FJ748749
LB251
DR058
DR085
FJ544230
ADG171
GQ895167
DR109
GU014568

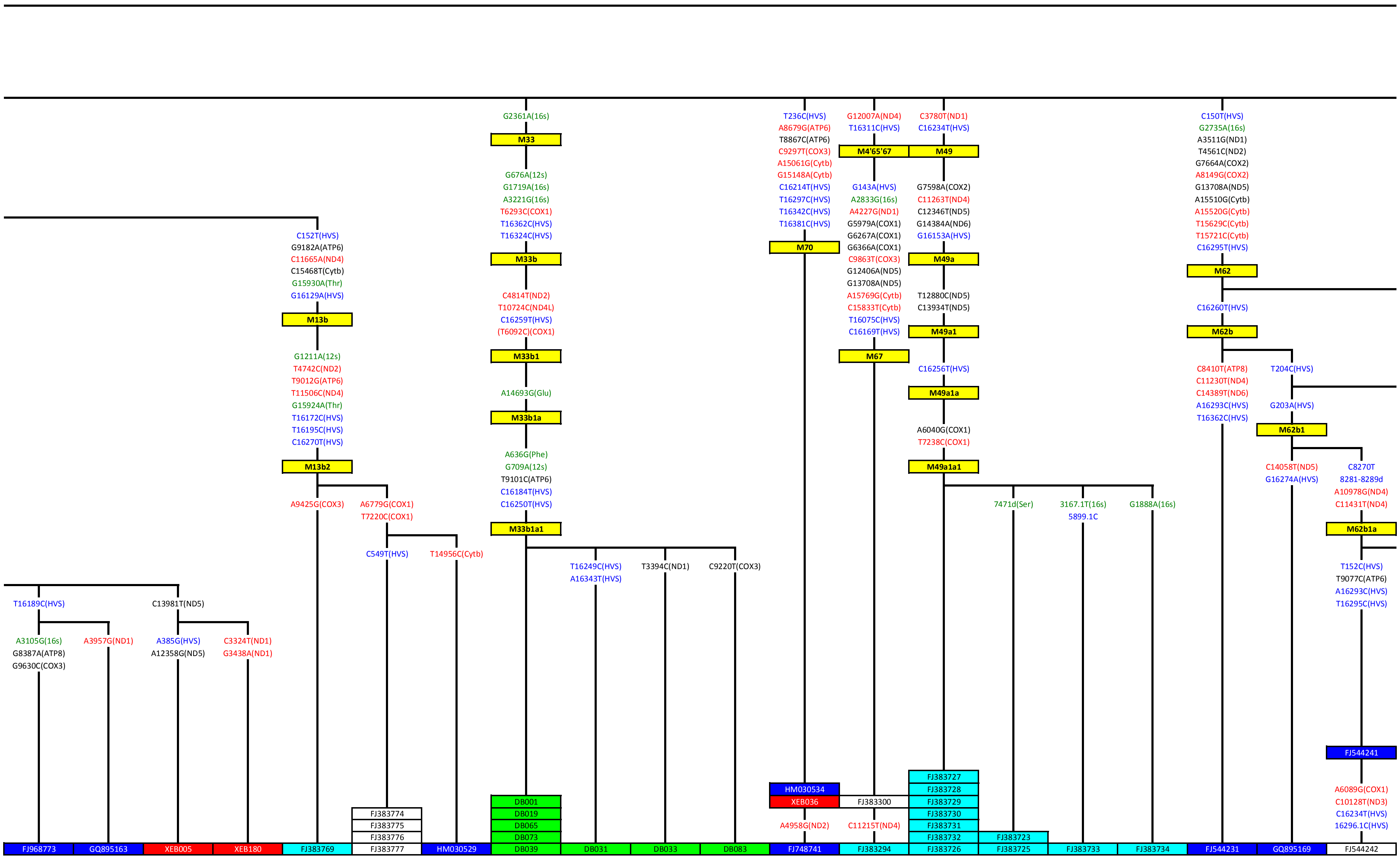
G2361A(16s)
M33
G676A(12s) G1719A(16s) A3221G(16s) T6293C(COX1) T16362C(HVS) T16324C(HVS)
M33b
C4814T(ND2) T10724C(ND4L) C16259T(HVS) (T6092C)(COX1)
M33b1
A14693G(Glu)
M33b1a
A636G(Phe) G709A(12s) T9101C(ATP6) C16184T(HVS) C16250T(HVS)
M33b1a1
T16249C(HVS) A16343T(HVS)
T3394C(ND1)
C9220T(COX3)
C152T(HVS) G9182A(ATP6) C11665A(ND4) C15468T(Cytb) G15930A(Thr) G16129A(HVS)
M13b
G1211A(12s) T4742C(ND2) T9012G(ATP6) T11506C(ND4) G15924A(Thr) T16172C(HVS) T16195C(HVS) C16270T(HVS)
M13b2
A9425G(COX3)
A6779G(COX1) T7220C(COX1)
C549T(HVS)
T14956C(Cytb)
T16189C(HVS)
C13981T(ND5)
A3105G(16s) G8387A(ATP8) G9630C(COX3)
A3957G(ND1)
A385G(HVS) A12358G(ND5)
C3324T(ND1) G3438A(ND1)
T236C(HVS) A8679G(ATP6) T8867C(ATP6) C9297T(COX3) A15061G(Cytb) G15148A(Cytb) C16214T(HVS) T16297C(HVS) T16342C(HVS) T16381C(HVS)
M70
A4958G(ND2)
G12007A(ND4) T16311C(HVS)
M4'65'67
G143A(HVS) A2833G(16s) A4227G(ND1) G5979A(COX1) G6267A(COX1) G6366A(COX1) C9863T(COX3) G12406A(ND5) G13708A(ND5) A15769G(Cytb) C15833T(Cytb) T16075C(HVS) C16169T(HVS)
M67
C11215T(ND4)
C3780T(ND1) C16234T(HVS)
M49
G7598A(COX2) C11263T(ND4) C12346T(ND5) G14384A(ND6) G16153A(HVS)
M49a
T12880C(ND5) C13934T(ND5)
M49a1
C16256T(HVS)
M49a1a
A6040G(COX1) T7238C(COX1)
M49a1a1
7471d(Ser)
3167.1T(16s) 5899.1C
G1888A(16s)
C150T(HVS) G2735A(16s) A3511G(ND1) T4561C(ND2) G7664A(COX2) A8149G(COX2) G13708A(ND5) A15510G(Cytb) A15520G(Cytb) T15629C(Cytb) T15721C(Cytb) C16295T(HVS)
M62
C16260T(HVS)
M62b
C8410T(ATP8) C11230T(ND4) C14389T(ND6) A16293C(HVS) T16362C(HVS)
T204C(HVS)
G203A(HVS)
M62b1
C14058T(ND5) G16274A(HVS)
C8270T 8281-8289d A10978G(ND4) C11431T(ND4)
M62b1a
T152C(HVS) T9077C(ATP6) A16293C(HVS) T16295C(HVS)
FJ544241
A6089G(COX1) C10128T(ND3) C16234T(HVS) 16296.1C(HVS)
FJ968773
GQ895163
XEB005
XEB180
FJ383769
FJ383774 FJ383775 FJ383776 FJ383777
HM030529
DB001 DB019 DB065 DB073 DB039
DB031
DB033
DB083
HM030534 XEB036 FJ748741
FJ383300 FJ383294
FJ383727 FJ383728 FJ383729 FJ383730 FJ383731 FJ383732 FJ383726
FJ383723 FJ383725
FJ383733
FJ383734
FJ544231
GQ895169
FJ544242

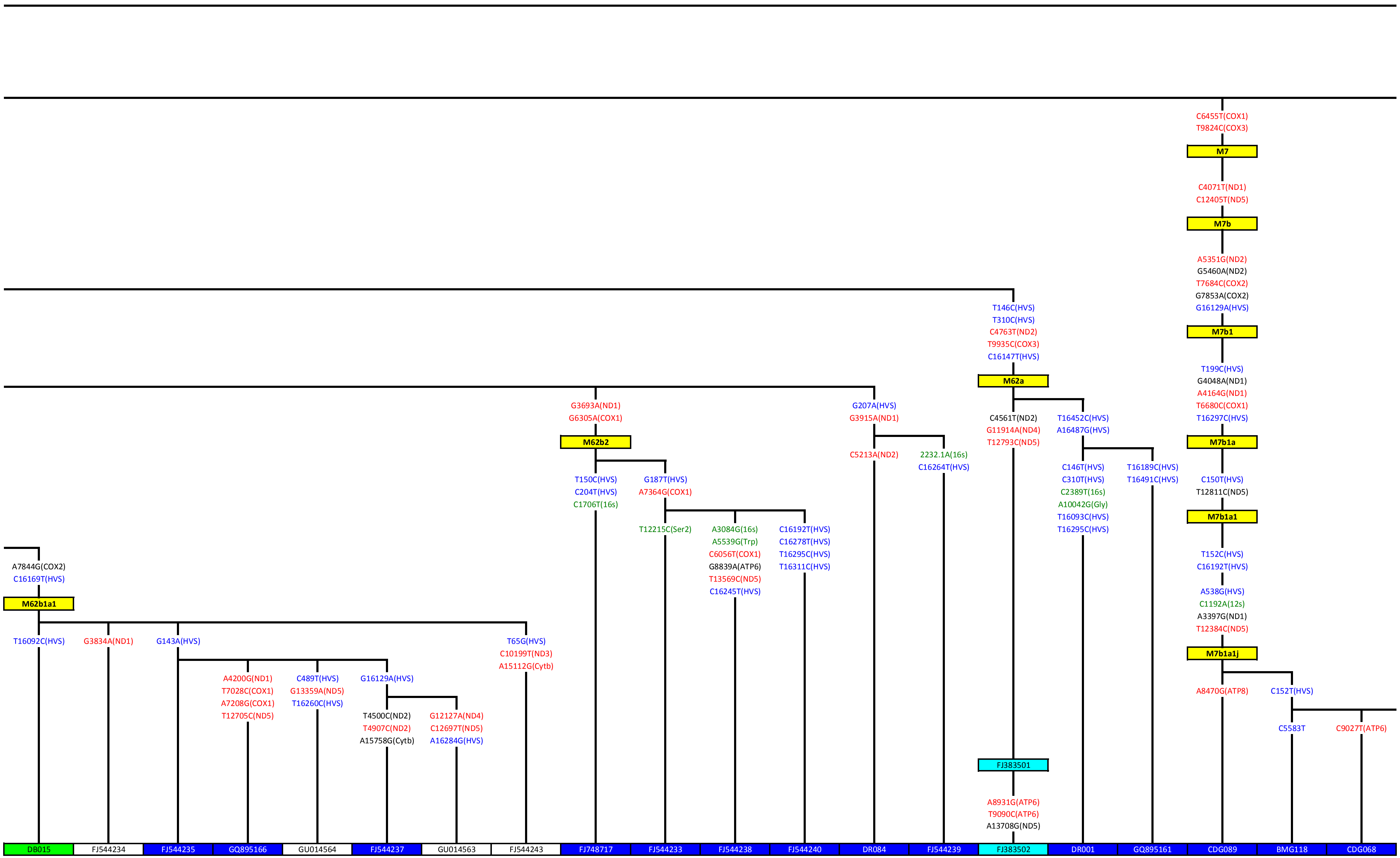

C6455T(COX1)
T9824C(COX3)
M7
C4071T(ND1)
C12405T(ND5)
M7b
A5351G(ND2)
G5460A(ND2)
T7684C(COX2)
G7853A(COX2)
G16129A(HVS)
M7b1
T199C(HVS)
G4048A(ND1)
A4164G(ND1)
T6680C(COX1)
T16297C(HVS)
M7b1a
C150T(HVS)
T12811C(ND5)
M7b1a1
T152C(HVS)
C16192T(HVS)
A538G(HVS)
C1192A(12s)
A3397G(ND1)
T12384C(ND5)
M7b1a1j
A8470G(ATP8)
C152T(HVS)
C5583T
C9027T(ATP6)
T146C(HVS)
T310C(HVS)
C4763T(ND2)
T9935C(COX3)
C16147T(HVS)
M62a
C4561T(ND2)
G11914A(ND4)
T12793C(ND5)
FJ383501
A8931G(ATP6)
T9090C(ATP6)
A13708G(ND5)
T16452C(HVS)
A16487G(HVS)
C146T(HVS)
C310T(HVS)
C2389T(16s)
A10042G(Gly)
T16093C(HVS)
T16295C(HVS)
T16189C(HVS)
T16491C(HVS)
G3693A(ND1)
G6305A(COX1)
M62b2
T150C(HVS)
C204T(HVS)
C1706T(16s)
G187T(HVS)
A7364G(COX1)
T12215C(Ser2)
A3084G(16s)
A5539G(Trp)
C6056T(COX1)
G8839A(ATP6)
T13569C(ND5)
C16245T(HVS)
C16192T(HVS)
C16278T(HVS)
T16295C(HVS)
T16311C(HVS)
G207A(HVS)
G3915A(ND1)
C5213A(ND2)
2232.1A(16s)
C16264T(HVS)
A7844G(COX2)
C16169T(HVS)
M62b1a1
T16092C(HVS)
G3834A(ND1)
G143A(HVS)
A4200G(ND1)
T7028C(COX1)
A7208G(COX1)
T12705C(ND5)
C489T(HVS)
G13359A(ND5)
T16260C(HVS)
G16129A(HVS)
T4500C(ND2)
T4907C(ND2)
A15758G(Cytb)
G12127A(ND4)
C12697T(ND5)
A16284G(HVS)
T65G(HVS)
C10199T(ND3)
A15112G(Cytb)
DB015
FJ544234
FJ544235
GQ895166
GU014564
FJ544237
GU014563
FJ544243
FJ748717
FJ544233
FJ544238
FJ544240
DR084
FJ544239
FJ383502
DR001
GQ895161
CDG089
BMG118
CDG068

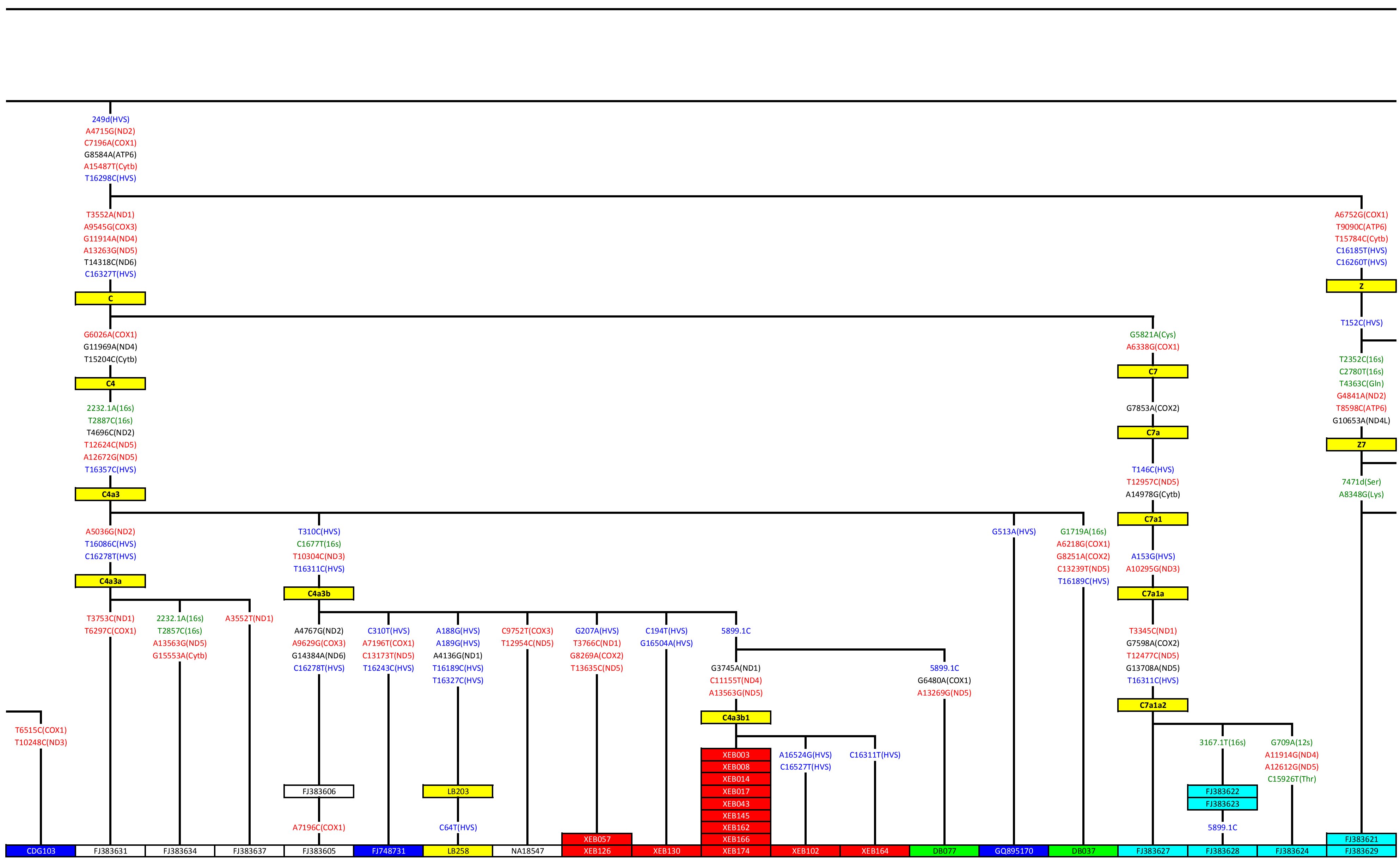

249d(HVS)
A4715G(ND2)
C7196A(COX1)
G8584A(ATP6)
A15487T(Cytb)
T16298C(HVS)
T3552A(ND1)
A9545G(COX3)
G11914A(ND4)
A13263G(ND5)
T14318C(ND6)
C16327T(HVS)
C
G6026A(COX1)
G11969A(ND4)
T15204C(Cytb)
C4
2232.1A(16s)
T2887C(16s)
T4696C(ND2)
T12624C(ND5)
A12672G(ND5)
T16357C(HVS)
C4a3
A5036G(ND2)
T16086C(HVS)
C16278T(HVS)
C4a3a
T3753C(ND1)
T6297C(COX1)
2232.1A(16s)
T2857C(16s)
A13563G(ND5)
G15553A(Cytb)
A3552T(ND1)
T310C(HVS)
C1677T(16s)
T10304C(ND3)
T16311C(HVS)
C4a3b
A4767G(ND2)
A9629G(COX3)
G14384A(ND6)
C16278T(HVS)
FJ383606
A7196C(COX1)
C310T(HVS)
A7196T(COX1)
C13173T(ND5)
T16243C(HVS)
A188G(HVS)
A189G(HVS)
A4136G(ND1)
T16189C(HVS)
T16327C(HVS)
LB203
C64T(HVS)
C9752T(COX3)
T12954C(ND5)
G207A(HVS)
T3766C(ND1)
G8269A(COX2)
T13635C(ND5)
C194T(HVS)
G16504A(HVS)
5899.1C
G3745A(ND1)
C11155T(ND4)
A13563G(ND5)
C4a3b1
XEB003
XEB008
XEB014
XEB017
XEB043
XEB145
XEB162
XEB166
A16524G(HVS)
C16527T(HVS)
C16311T(HVS)
5899.1C
G6480A(COX1)
A13269G(ND5)
G513A(HVS)
G1719A(16s)
A6218G(COX1)
G8251A(COX2)
C13239T(ND5)
T16189C(HVS)
G5821A(Cys)
A6338G(COX1)
C7
G7853A(COX2)
C7a
T146C(HVS)
T12957C(ND5)
A14978G(Cytb)
C7a1
A153G(HVS)
A10295G(ND3)
C7a1a
T3345C(ND1)
G7598A(COX2)
T12477C(ND5)
G13708A(ND5)
T16311C(HVS)
C7a1a2
3167.1T(16s)
FJ383622
FJ383623
5899.1C
G709A(12s)
A11914G(ND4)
A12612G(ND5)
C15926T(Thr)
A6752G(COX1)
T9090C(ATP6)
T15784C(Cytb)
C16185T(HVS)
C16260T(HVS)
Z
T152C(HVS)
T2352C(16s)
C2780T(16s)
T4363C(Gln)
G4841A(ND2)
T8598C(ATP6)
G10653A(ND4L)
Z7
7471d(Ser)
A8348G(Lys)
FJ383621
T6515C(COX1)
T10248C(ND3)
XEB057
CDG103
FJ383631
FJ383634
FJ383637
FJ383605
FJ748731
LB258
NA18547
XEB126
XEB130
XEB174
XEB102
XEB164
DB077
GQ895170
DB037
FJ383627
FJ383628
FJ383624
FJ383629

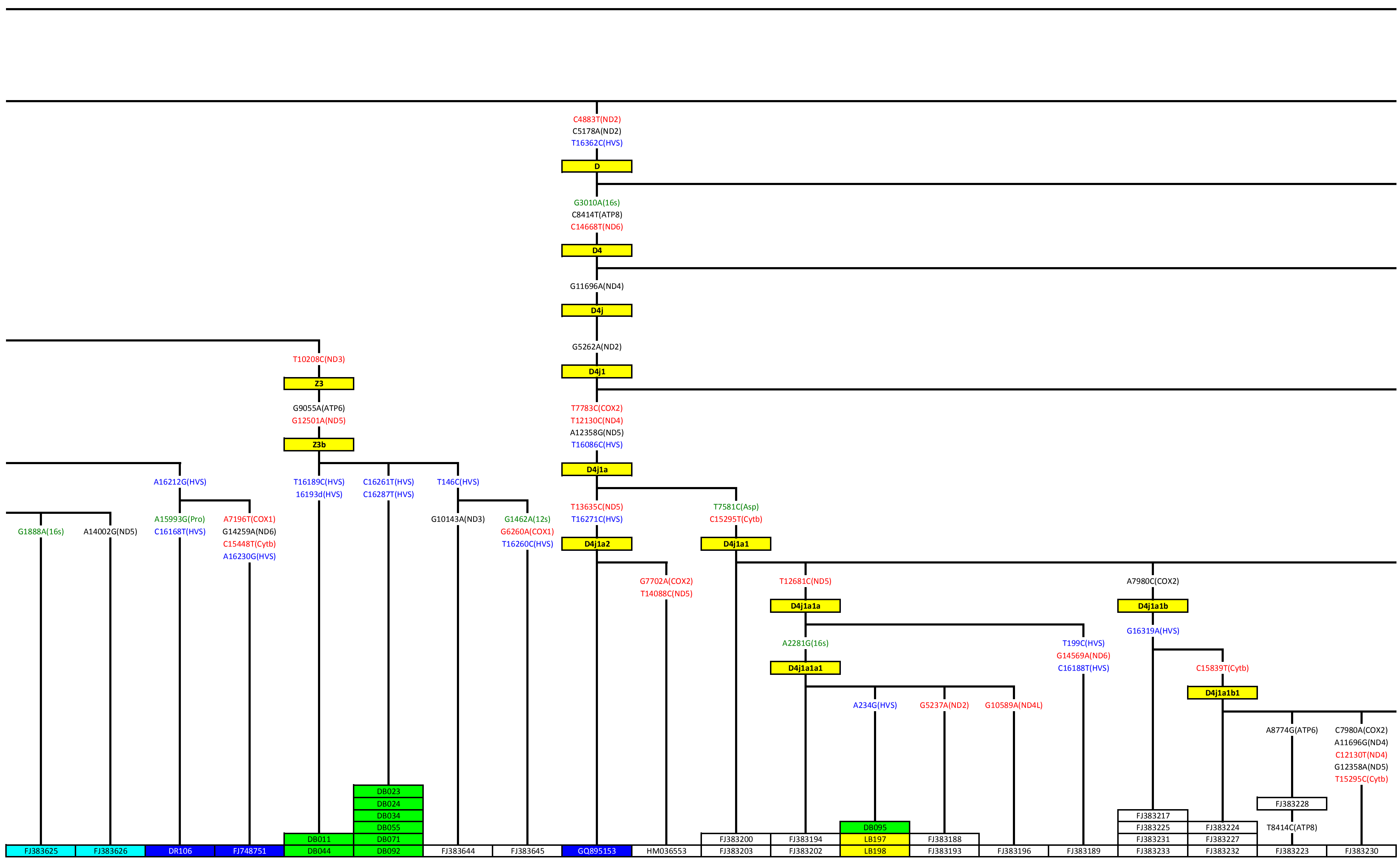

C4883T(ND2)
C5178A(ND2)
T16362C(HVS)
D
G3010A(16s)
C8414T(ATP8)
C14668T(ND6)
D4
G11696A(ND4)
D4j
G5262A(ND2)
D4j1
T7783C(COX2)
T12130C(ND4)
A12358G(ND5)
T16086C(HVS)
D4j1a
T10208C(ND3)
Z3
G9055A(ATP6)
G12501A(ND5)
Z3b
A16212G(HVS)
T16189C(HVS)
16193d(HVS)
C16261T(HVS)
C16287T(HVS)
T146C(HVS)
G1888A(16s)
A14002G(ND5)
A15993G(Pro)
C16168T(HVS)
A7196T(COX1)
G14259A(ND6)
C15448T(Cytb)
A16230G(HVS)
G10143A(ND3)
G1462A(12s)
G6260A(COX1)
T16260C(HVS)
T13635C(ND5)
T16271C(HVS)
D4j1a2
T7581C(Asp)
C15295T(Cytb)
D4j1a1
G7702A(COX2)
T14088C(ND5)
T12681C(ND5)
D4j1a1a
A2281G(16s)
D4j1a1a1
T199C(HVS)
G14569A(ND6)
C16188T(HVS)
A234G(HVS)
G5237A(ND2)
G10589A(ND4L)
A7980C(COX2)
D4j1a1b
G16319A(HVS)
C15839T(Cytb)
D4j1a1b1
A8774G(ATP6)
C7980A(COX2)
A11696G(ND4)
C12130T(ND4)
G12358A(ND5)
T15295C(Cytb)
T8414C(ATP8)
FJ383625
FJ383626
DR106
FJ748751
DB011
DB044
DB023
DB024
DB034
DB055
DB071
DB092
FJ383644
FJ383645
GQ895153
HM036553
FJ383200
FJ383203
FJ383194
FJ383202
DB095
LB197
LB198
FJ383188
FJ383193
FJ383196
FJ383189
FJ383217
FJ383225
FJ383231
FJ383233
FJ383224
FJ383227
FJ383232
FJ383228
FJ383223
FJ383230

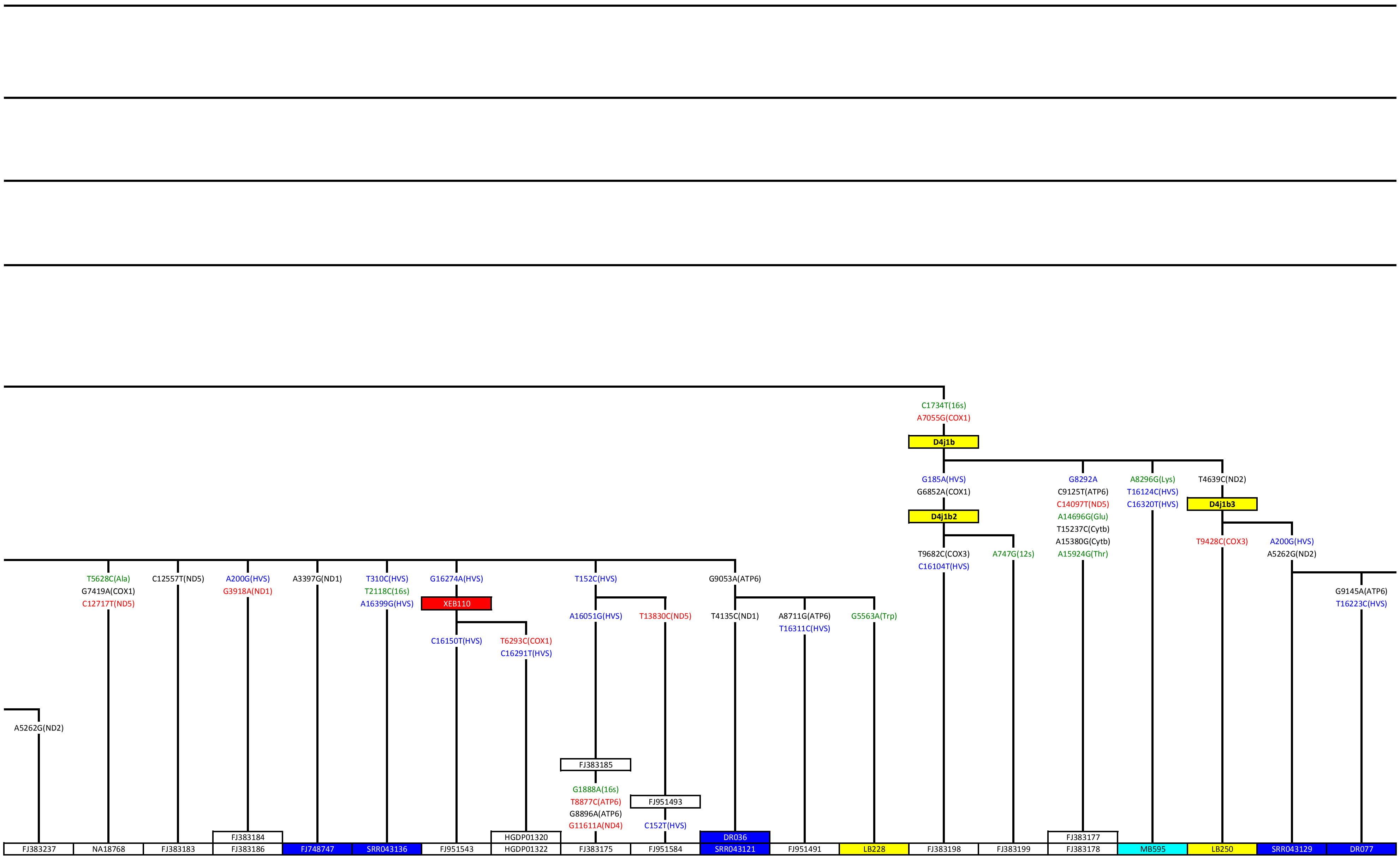

C1734T(16s)
A7055G(COX1)
D4j1b
G185A(HVS)
G6852A(COX1)
D4j1b2
G8292A
C9125T(ATP6)
C14097T(ND5)
A14696G(Glu)
T15237C(Cytb)
A15380G(Cytb)
A15924G(Thr)
A8296G(Lys)
T16124C(HVS)
C16320T(HVS)
T4639C(ND2)
D4j1b3
T9428C(COX3)
A200G(HVS)
A5262G(ND2)
T9682C(COX3)
C16104T(HVS)
A747G(12s)
T5628C(Ala)
G7419A(COX1)
C12717T(ND5)
C12557T(ND5)
A200G(HVS)
G3918A(ND1)
A3397G(ND1)
T310C(HVS)
T2118C(16s)
A16399G(HVS)
G16274A(HVS)
XEB110
C16150T(HVS)
T6293C(COX1)
C16291T(HVS)
T152C(HVS)
A16051G(HVS)
T13830C(ND5)
G9053A(ATP6)
T4135C(ND1)
A8711G(ATP6)
T16311C(HVS)
G5563A(Trp)
G9145A(ATP6)
T16223C(HVS)
A5262G(ND2)
FJ383185
G1888A(16s)
T8877C(ATP6)
G8896A(ATP6)
G11611A(ND4)
FJ951493
C152T(HVS)
FJ383184
HGDP01320
DR036
FJ383177
FJ383237
NA18768
FJ383183
FJ383186
FJ748747
SRR043136
FJ951543
HGDP01322
FJ383175
FJ951584
SRR043121
FJ951491
LB228
FJ383198
FJ383199
FJ383178
MB595
LB250
SRR043129
DR077

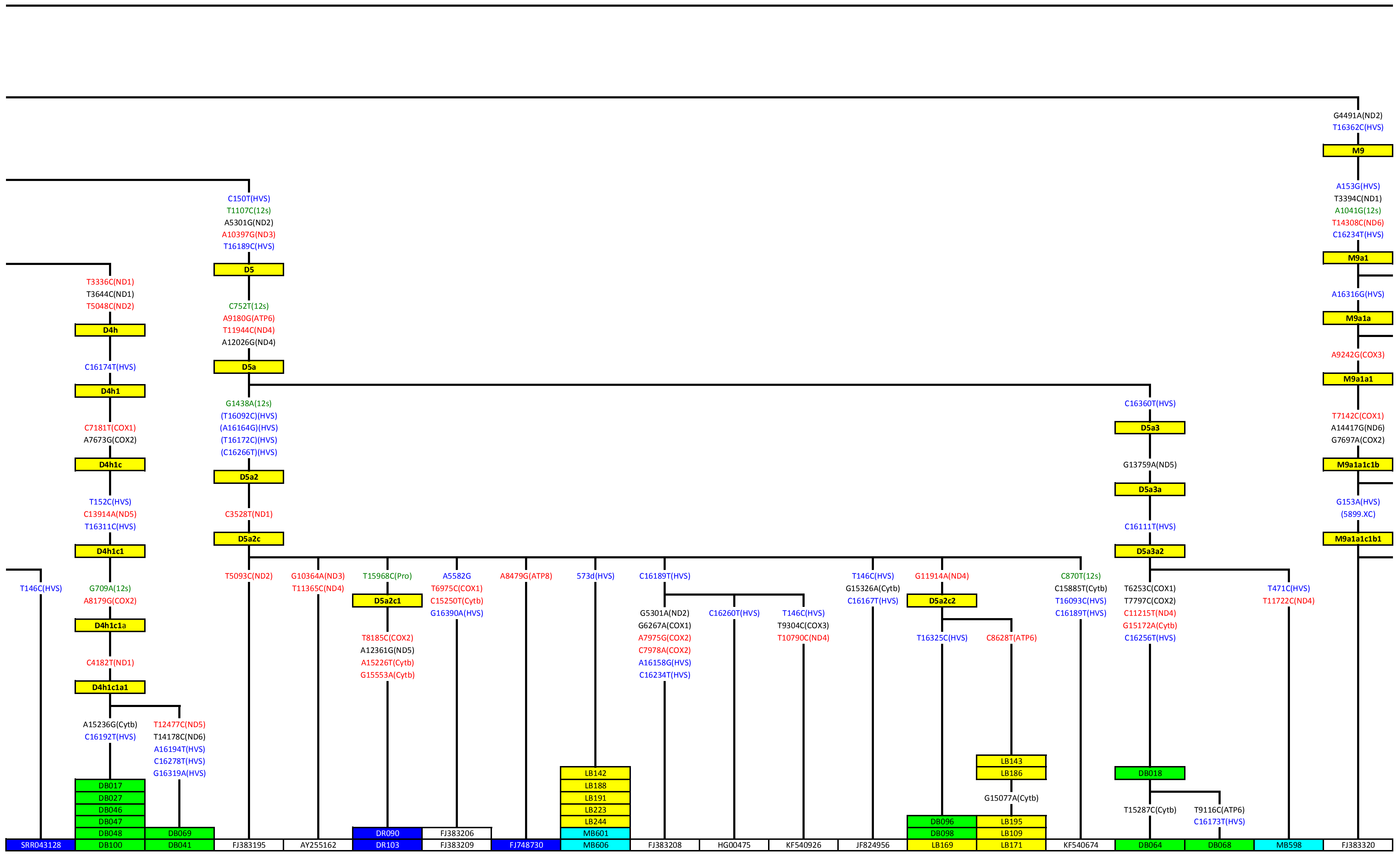
G4491A(ND2)
T16362C(HVS)
M9
A153G(HVS)
T3394C(ND1)
A1041G(12s)
T14308C(ND6)
C16234T(HVS)
M9a1
A16316G(HVS)
M9a1a
A9242G(COX3)
M9a1a1
T7142C(COX1)
A14417G(ND6)
G7697A(COX2)
M9a1a1c1b
G153A(HVS)
(5899.XC)
M9a1a1c1b1
C150T(HVS)
T1107C(12s)
A5301G(ND2)
A10397G(ND3)
T16189C(HVS)
D5
C752T(12s)
A9180G(ATP6)
T11944C(ND4)
A12026G(ND4)
D5a
G1438A(12s)
(T16092C)(HVS)
(A16164G)(HVS)
(T16172C)(HVS)
(C16266T)(HVS)
D5a2
C3528T(ND1)
D5a2c
T3336C(ND1)
T3644C(ND1)
T5048C(ND2)
D4h
C16174T(HVS)
D4h1
C7181T(COX1)
A7673G(COX2)
D4h1c
T152C(HVS)
C13914A(ND5)
T16311C(HVS)
D4h1c1
G709A(12s)
A8179G(COX2)
D4h1c1a
C4182T(ND1)
D4h1c1a1
A15236G(Cytb)
C16192T(HVS)
T12477C(ND5)
T14178C(ND6)
A16194T(HVS)
C16278T(HVS)
G16319A(HVS)
T146C(HVS)
T5093C(ND2)
G10364A(ND3)
T11365C(ND4)
T15968C(Pro)
D5a2c1
T8185C(COX2)
A12361G(ND5)
A15226T(Cytb)
G15553A(Cytb)
A5582G
T6975C(COX1)
C15250T(Cytb)
G16390A(HVS)
A8479G(ATP8)
573d(HVS)
C16189T(HVS)
G5301A(ND2)
G6267A(COX1)
A7975G(COX2)
C7978A(COX2)
A16158G(HVS)
C16234T(HVS)
C16260T(HVS)
T146C(HVS)
T9304C(COX3)
T10790C(ND4)
T146C(HVS)
G15326A(Cytb)
C16167T(HVS)
G11914A(ND4)
D5a2c2
T16325C(HVS)
C8628T(ATP6)
LB143
LB186
G15077A(Cytb)
C870T(12s)
C15885T(Cytb)
T16093C(HVS)
C16189T(HVS)
C16360T(HVS)
D5a3
G13759A(ND5)
D5a3a
C16111T(HVS)
D5a3a2
T6253C(COX1)
T7797C(COX2)
C11215T(ND4)
G15172A(Cytb)
C16256T(HVS)
DB018
T15287C(Cytb)
T9116C(ATP6)
C16173T(HVS)
T471C(HVS)
T11722C(ND4)
DB017
DB027
DB046
DB047
DB048
DB069
LB142
LB188
LB191
LB223
LB244
MB601
FJ383206
DB096
DB098
LB195
LB109
SRR043128
DB100
DB041
FJ383195
AY255162
DR090
DR103
FJ383209
FJ748730
MB606
FJ383208
HG00475
KF540926
JF824956
LB169
LB171
KF540674
DB064
DB068
MB598
FJ383320

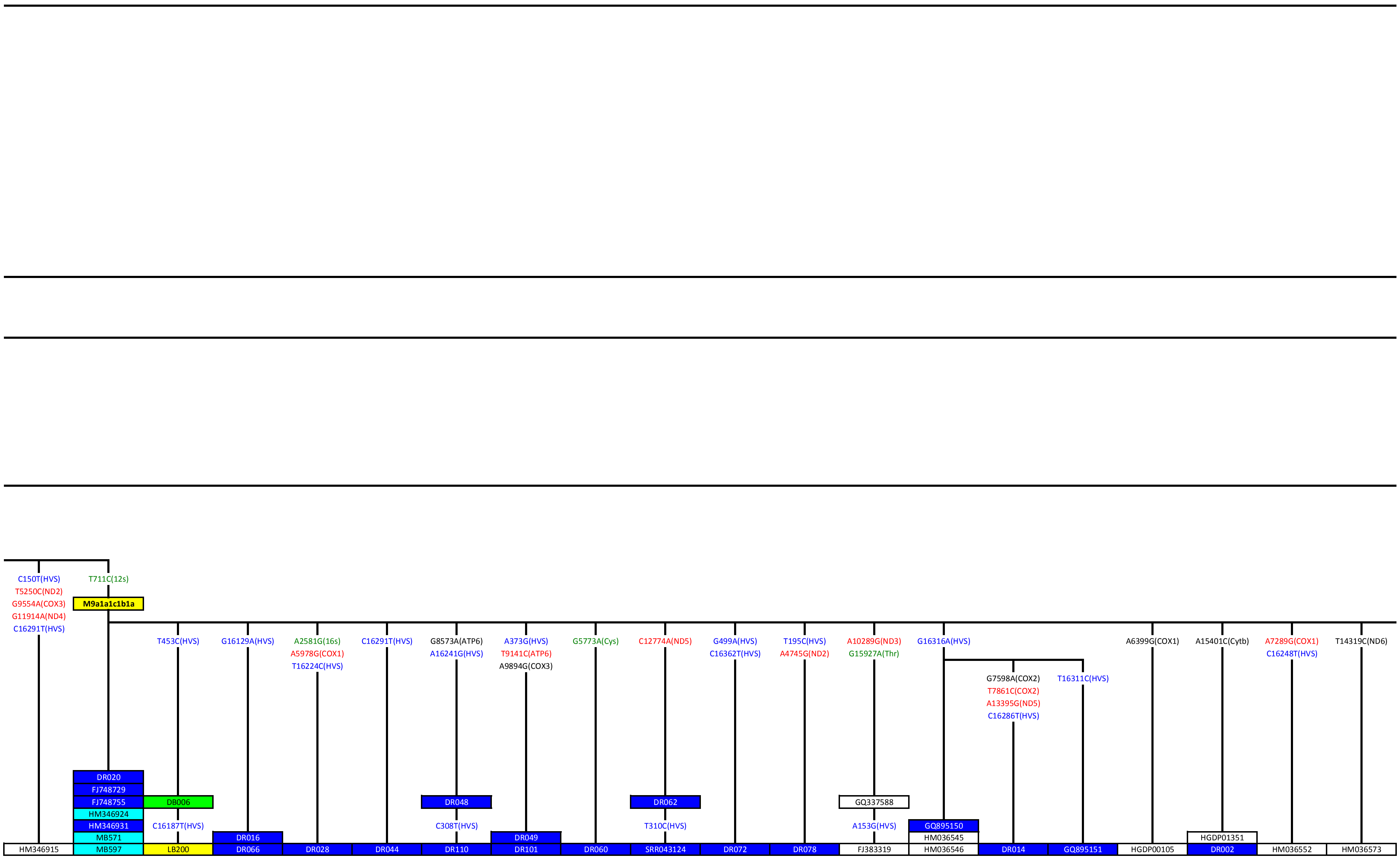

C150T(HVS)
T5250C(ND2)
G9554A(COX3)
G11914A(ND4)
C16291T(HVS)
T711C(12s)
M9a1a1c1b1a
T453C(HVS)
G16129A(HVS)
A2581G(16s)
A5978G(COX1)
T16224C(HVS)
C16291T(HVS)
G8573A(ATP6)
A16241G(HVS)
A373G(HVS)
T9141C(ATP6)
A9894G(COX3)
G5773A(Cys)
C12774A(ND5)
G499A(HVS)
C16362T(HVS)
T195C(HVS)
A4745G(ND2)
A10289G(ND3)
G15927A(Thr)
G16316A(HVS)
A6399G(COX1)
A15401C(Cytb)
A7289G(COX1)
C16248T(HVS)
T14319C(ND6)
G7598A(COX2)
T7861C(COX2)
A13395G(ND5)
C16286T(HVS)
T16311C(HVS)
DR020
FJ748729
FJ748755
HM346924
HM346931
MB571
DB006
C16187T(HVS)
DR048
C308T(HVS)
DR062
T310C(HVS)
GQ337588
A153G(HVS)
GQ895150
HM036545
DR016
DR049
HGDP01351
HM346915
MB597
LB200
DR066
DR028
DR044
DR110
DR101
DR060
SRR043124
DR072
DR078
FJ383319
HM036546
DR014
GQ895151
HGDP00105
DR002
HM036552
HM036573

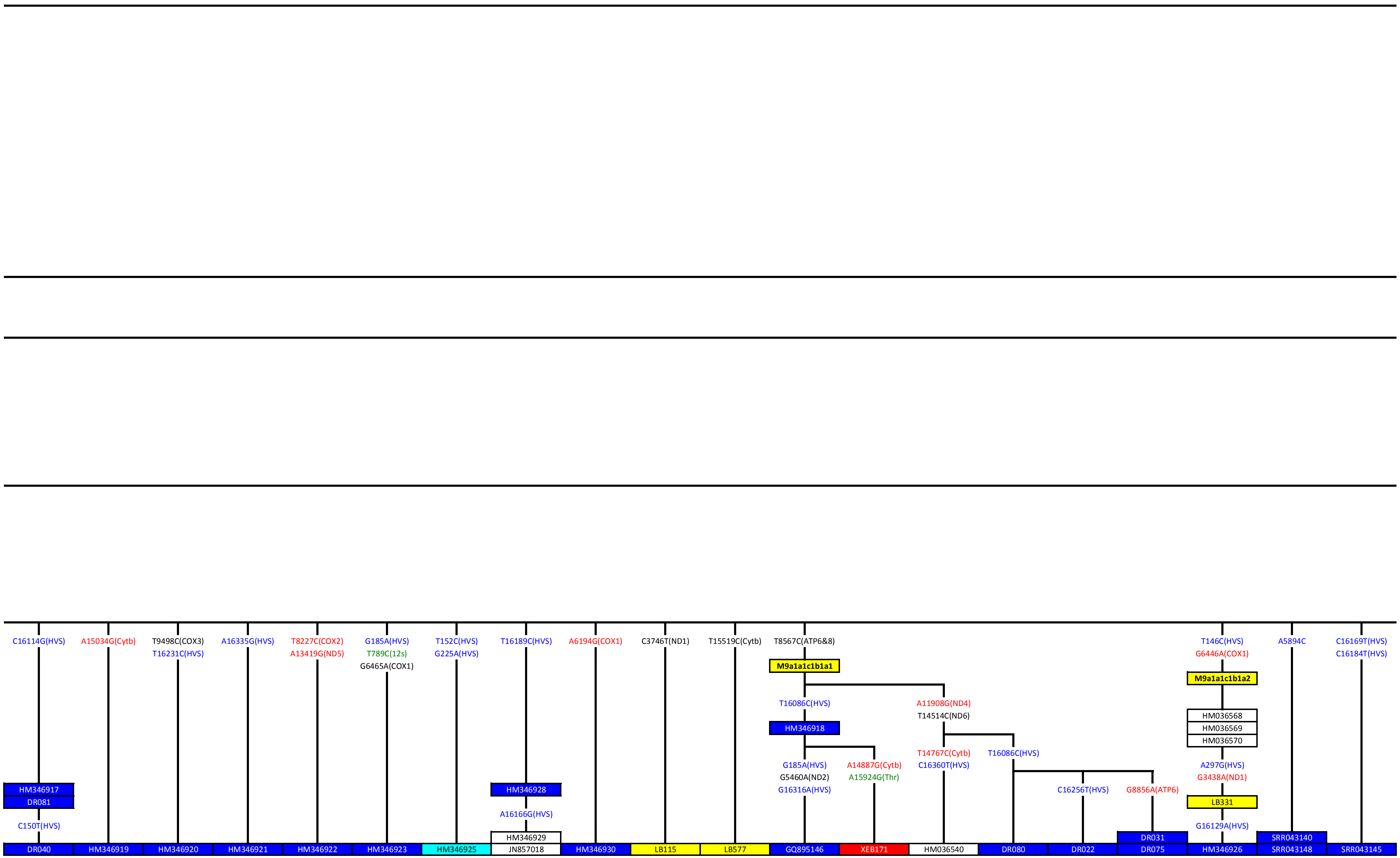

C16114G(HVS)
A15034G(Cytb)
T9498C(COX3)
T16231C(HVS)
A16335G(HVS)
T8227C(COX2)
A13419G(ND5)
G185A(HVS)
T789C(12s)
G6465A(COX1)
T152C(HVS)
G225A(HVS)
T16189C(HVS)
A6194G(COX1)
C3746T(ND1)
T15519C(Cytb)
T8567C(ATP6&8)
M9a1a1c1b1a1
T16086C(HVS)
HM346918
G185A(HVS)
G5460A(ND2)
G16316A(HVS)
A14887G(Cytb)
A15924G(Thr)
A11908G(ND4)
T14514C(ND6)
T14767C(Cytb)
C16360T(HVS)
T16086C(HVS)
C16256T(HVS)
G8856A(ATP6)
T146C(HVS)
G6446A(COX1)
M9a1a1c1b1a2
HM036568
HM036569
HM036570
A297G(HVS)
G3438A(ND1)
LB331
G16129A(HVS)
A5894C
C16169T(HVS)
C16184T(HVS)
HM346917
DR081
C150T(HVS)
HM346928
A16166G(HVS)
HM346929
DR031
SRR043140
DR040
HM346919
HM346920
HM346921
HM346922
HM346923
HM346925
JN857018
HM346930
LB115
LB577
GQ895146
XEB171
HM036540
DR080
DR022
DR075
HM346926
SRR043148
SRR043145

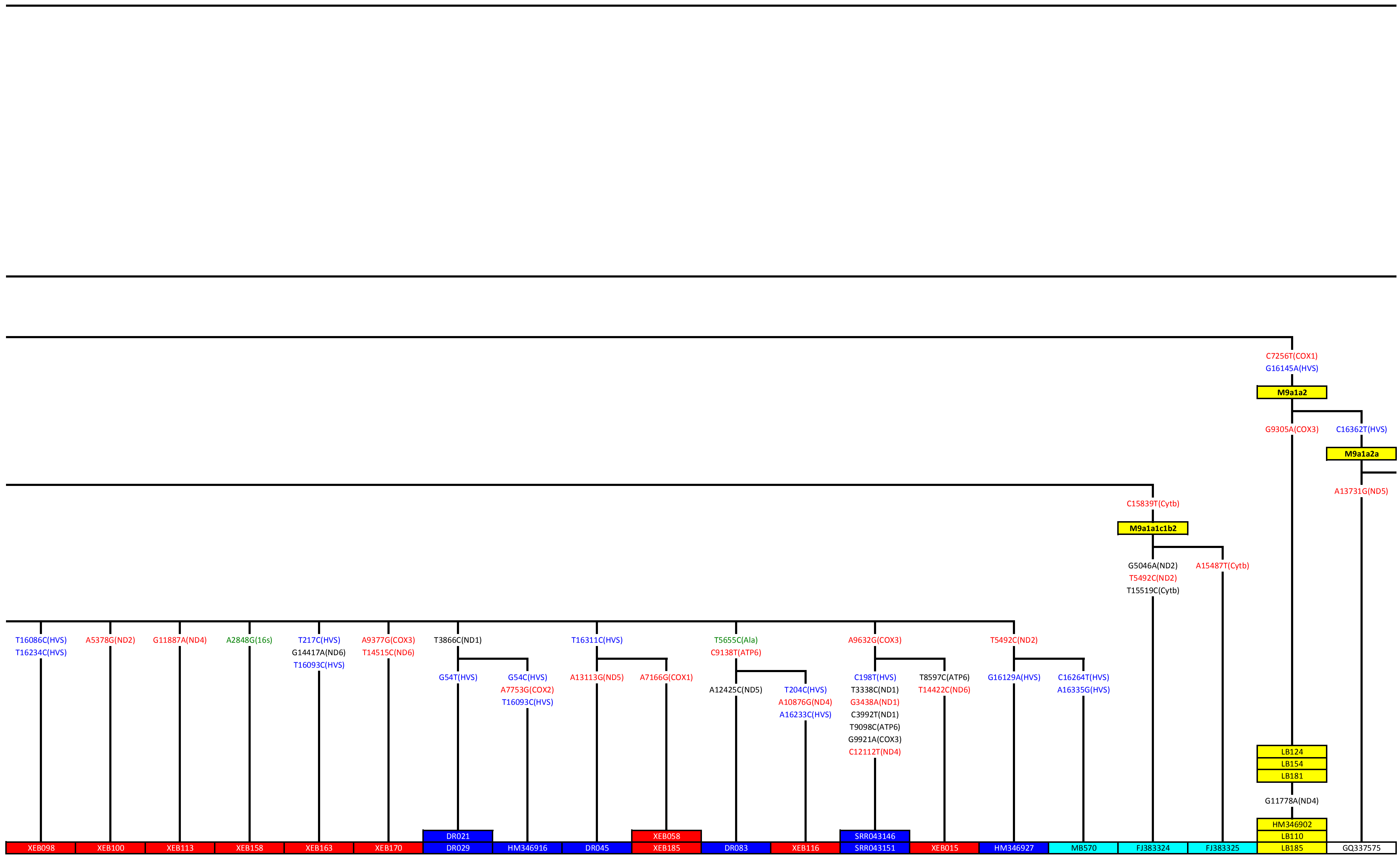
C7256T(COX1)
G16145A(HVS)
M9a1a2
G9305A(COX3)
C16362T(HVS)
M9a1a2a
A13731G(ND5)
C15839T(Cytb)
M9a1a1c1b2
G5046A(ND2)
T5492C(ND2)
T15519C(Cytb)
A15487T(Cytb)
T16086C(HVS)
T16234C(HVS)
A5378G(ND2)
G11887A(ND4)
A2848G(16s)
T217C(HVS)
G14417A(ND6)
T16093C(HVS)
A9377G(COX3)
T14515C(ND6)
T3866C(ND1)
G54T(HVS)
G54C(HVS)
A7753G(COX2)
T16093C(HVS)
T16311C(HVS)
A13113G(ND5)
A7166G(COX1)
T5655C(Ala)
C9138T(ATP6)
A12425C(ND5)
T204C(HVS)
A10876G(ND4)
A16233C(HVS)
A9632G(COX3)
C198T(HVS)
T3338C(ND1)
G3438A(ND1)
C3992T(ND1)
T9098C(ATP6)
G9921A(COX3)
C12112T(ND4)
T8597C(ATP6)
T14422C(ND6)
T5492C(ND2)
G16129A(HVS)
C16264T(HVS)
A16335G(HVS)
LB124
LB154
LB181
G11778A(ND4)
HM346902
LB110
DR021
XEB058
SRR043146
XEB098
XEB100
XEB113
XEB158
XEB163
XEB170
DR029
HM346916
DR045
XEB185
DR083
XEB116
SRR043151
XEB015
HM346927
MB570
FJ383324
FJ383325
LB185
GQ337575

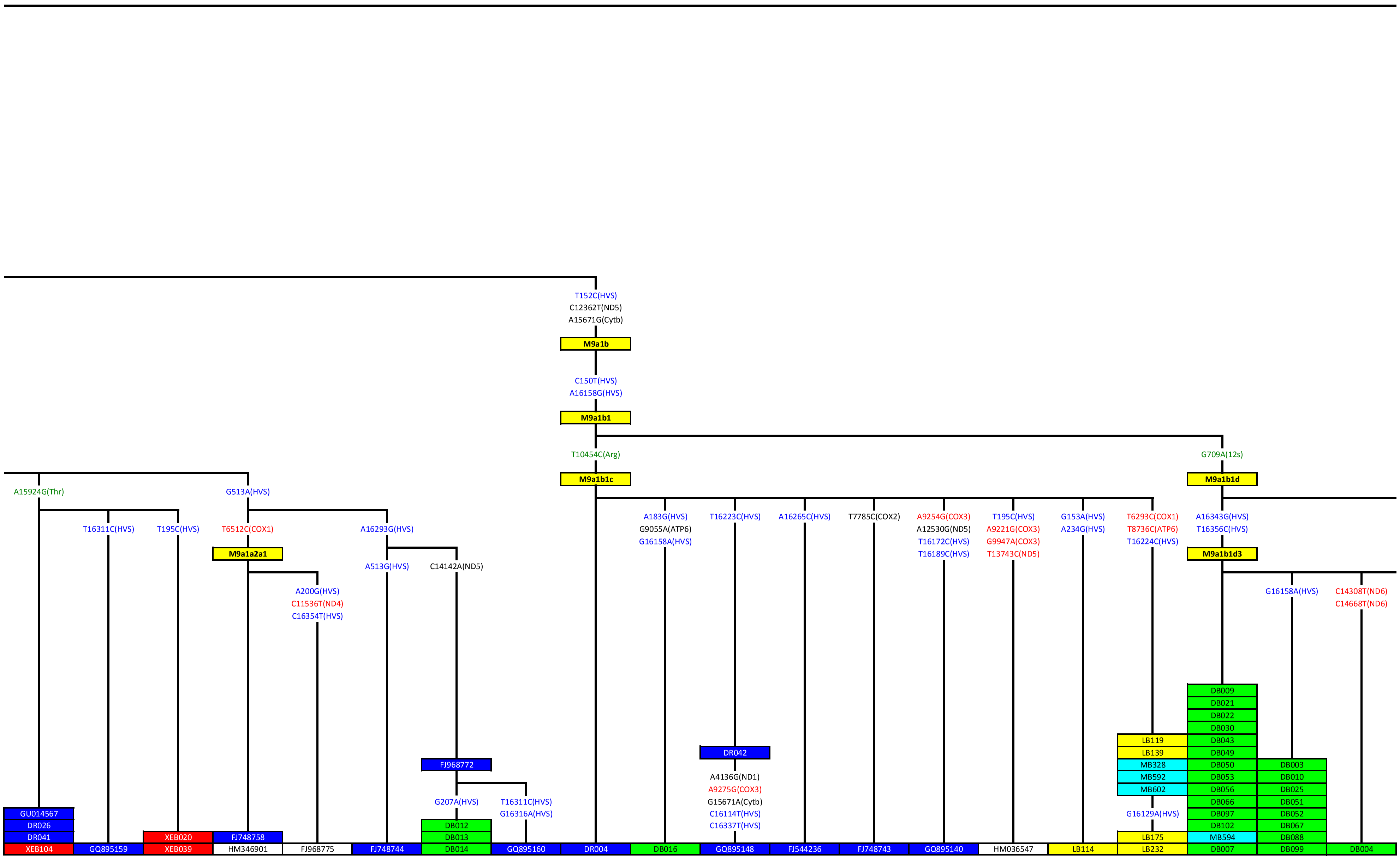

T152C(HVS)
C12362T(ND5)
A15671G(Cytb)
M9a1b
C150T(HVS)
A16158G(HVS)
M9a1b1
T10454C(Arg)
M9a1b1c
G709A(12s)
M9a1b1d
A15924G(Thr)
G513A(HVS)
T16311C(HVS)
T195C(HVS)
T6512C(COX1)
M9a1a2a1
A16293G(HVS)
A513G(HVS)
C14142A(ND5)
A200G(HVS)
C11536T(ND4)
C16354T(HVS)
A183G(HVS)
G9055A(ATP6)
G16158A(HVS)
T16223C(HVS)
A16265C(HVS)
T7785C(COX2)
A9254G(COX3)
A12530G(ND5)
T16172C(HVS)
T16189C(HVS)
T195C(HVS)
A9221G(COX3)
G9947A(COX3)
T13743C(ND5)
G153A(HVS)
A234G(HVS)
T6293C(COX1)
T8736C(ATP6)
T16224C(HVS)
A16343G(HVS)
T16356C(HVS)
M9a1b1d3
G16158A(HVS)
C14308T(ND6)
C14668T(ND6)
FJ968772
G207A(HVS)
T16311C(HVS)
G16316A(HVS)
DR042
A4136G(ND1)
A9275G(COX3)
G15671A(Cytb)
C16114T(HVS)
C16337T(HVS)
G16129A(HVS)
GU014567
DR026
DR041
XEB104
GQ895159
XEB020
XEB039
FJ748758
HM346901
FJ968775
FJ748744
DB012
DB013
DB014
GQ895160
DR004
DB016
GQ895148
FJ544236
FJ748743
GQ895140
HM036547
LB114
LB119
LB139
MB328
MB592
MB602
LB175
LB232
DB009
DB021
DB022
DB030
DB043
DB049
DB050
DB053
DB056
DB066
DB097
DB102
MB594
DB007
DB003
DB010
DB025
DB051
DB052
DB067
DB088
DB099
DB004

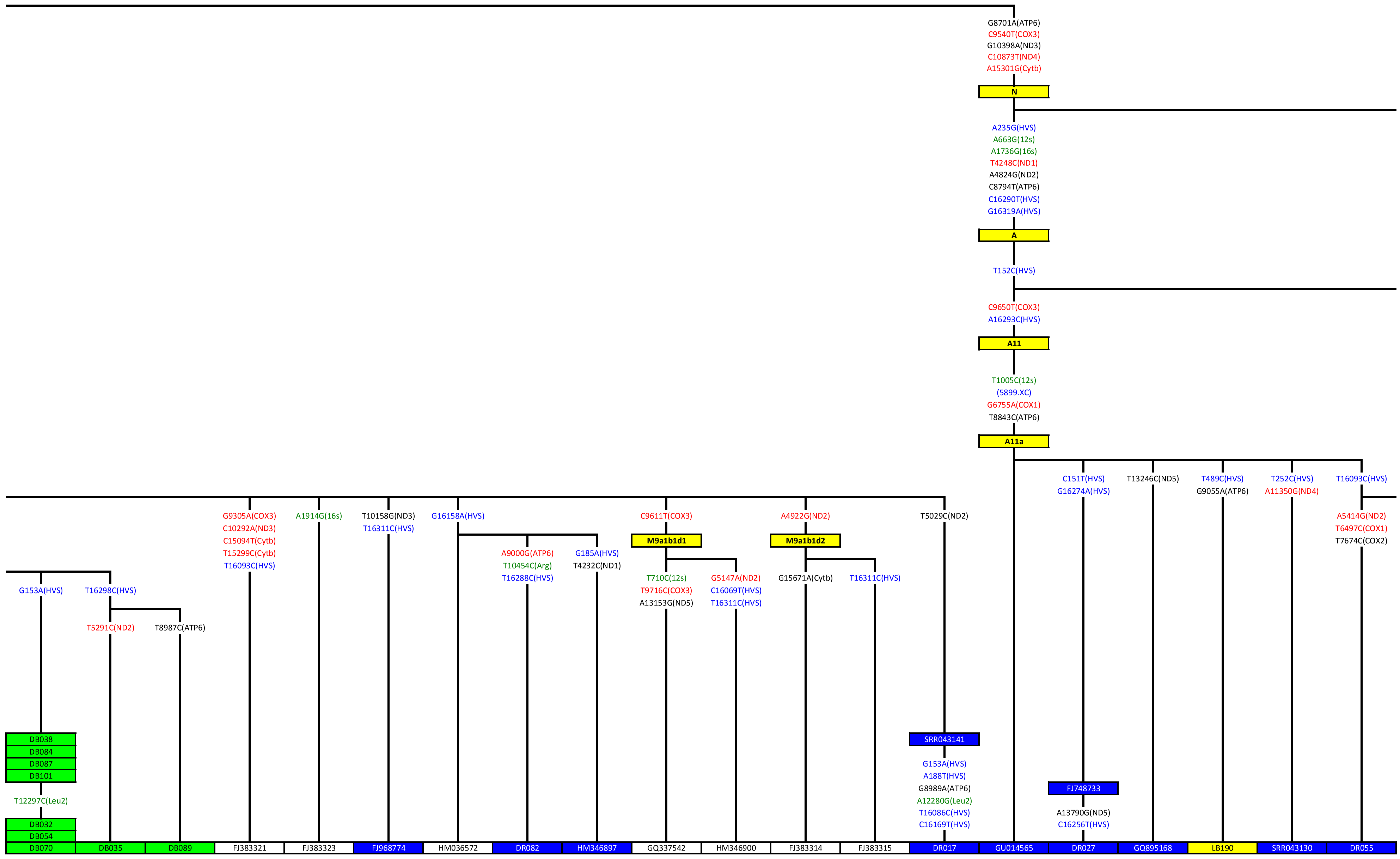
G8701A(ATP6)
C9540T(COX3)
G10398A(ND3)
C10873T(ND4)
A15301G(Cytb)
N
A235G(HVS)
A663G(12s)
A1736G(16s)
T4248C(ND1)
A4824G(ND2)
C8794T(ATP6)
C16290T(HVS)
G16319A(HVS)
A
T152C(HVS)
C9650T(COX3)
A16293C(HVS)
A11
T1005C(12s)
(5899.XC)
G6755A(COX1)
T8843C(ATP6)
A11a
C151T(HVS)
G16274A(HVS)
T13246C(ND5)
T489C(HVS)
G9055A(ATP6)
T252C(HVS)
A11350G(ND4)
T16093C(HVS)
A5414G(ND2)
T6497C(COX1)
T7674C(COX2)
G9305A(COX3)
C10292A(ND3)
C15094T(Cytb)
T15299C(Cytb)
T16093C(HVS)
A1914G(16s)
T10158G(ND3)
T16311C(HVS)
G16158A(HVS)
A9000G(ATP6)
T10454C(Arg)
T16288C(HVS)
G185A(HVS)
T4232C(ND1)
C9611T(COX3)
M9a1b1d1
T710C(12s)
T9716C(COX3)
A13153G(ND5)
G5147A(ND2)
C16069T(HVS)
T16311C(HVS)
A4922G(ND2)
M9a1b1d2
G15671A(Cytb)
T16311C(HVS)
T5029C(ND2)
SRR043141
G153A(HVS)
A188T(HVS)
G8989A(ATP6)
A12280G(Leu2)
T16086C(HVS)
C16169T(HVS)
FJ748733
A13790G(ND5)
C16256T(HVS)
G153A(HVS)
T16298C(HVS)
T5291C(ND2)
T8987C(ATP6)
DB038
DB084
DB087
DB101
T12297C(Leu2)
DB032
DB054
DB070
DB035
DB089
FJ383321
FJ383323
FJ968774
HM036572
DR082
HM346897
GQ337542
HM346900
FJ383314
FJ383315
DR017
GU014565
DR027
GQ895168
LB190
SRR043130
DR055

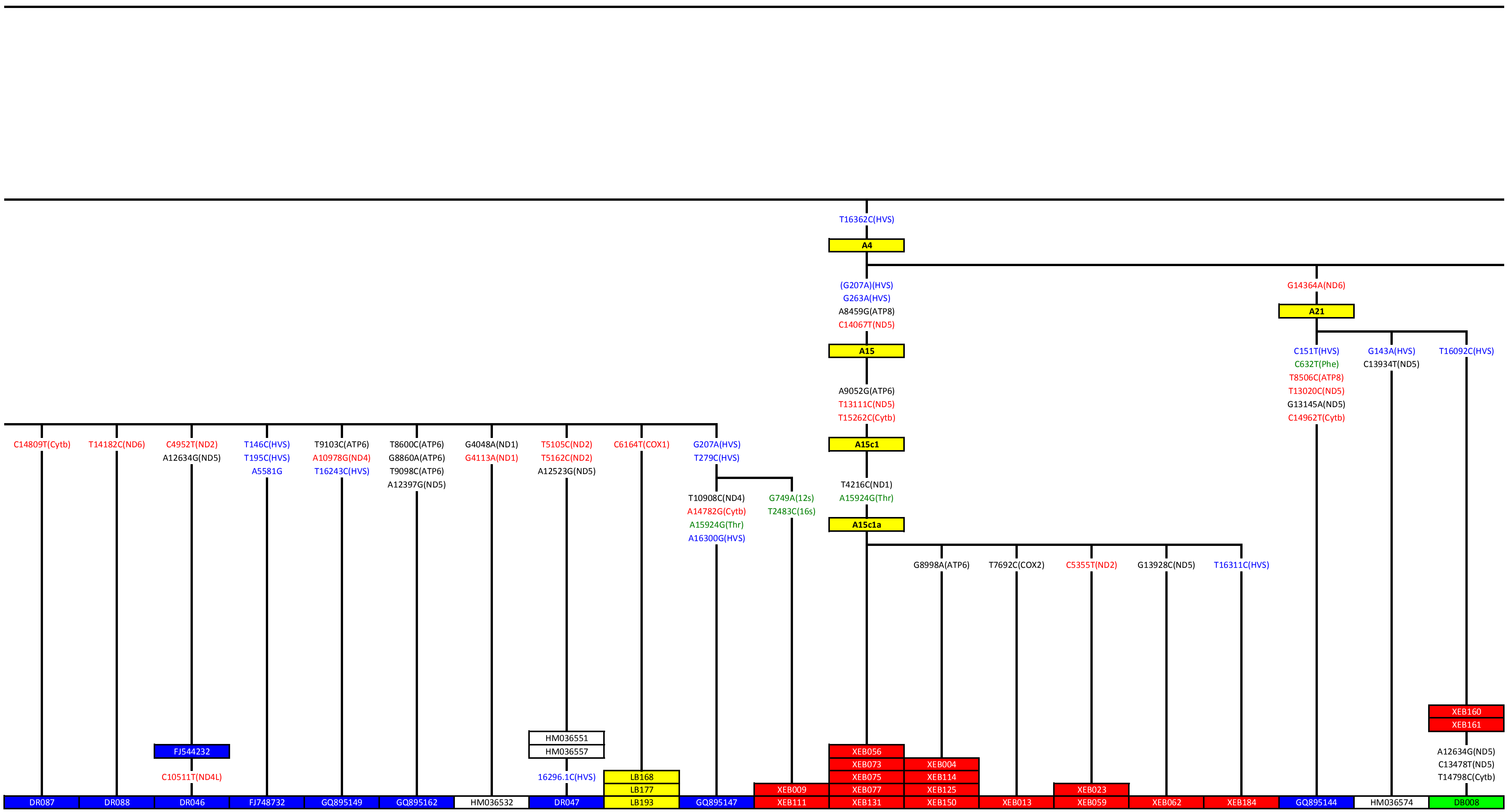

T16362C(HVS)
A4
(G207A)(HVS)
G263A(HVS)
A8459G(ATP8)
C14067T(ND5)
A15
A9052G(ATP6)
T13111C(ND5)
T15262C(Cytb)
A15c1
T4216C(ND1)
A15924G(Thr)
A15c1a
G14364A(ND6)
A21
C151T(HVS)
C632T(Phe)
T8506C(ATP8)
T13020C(ND5)
G13145A(ND5)
C14962T(Cytb)
G143A(HVS)
C13934T(ND5)
T16092C(HVS)
C14809T(Cytb)
T14182C(ND6)
C4952T(ND2)
A12634G(ND5)
T146C(HVS)
T195C(HVS)
A5581G
T9103C(ATP6)
A10978G(ND4)
T16243C(HVS)
T8600C(ATP6)
G8860A(ATP6)
T9098C(ATP6)
A12397G(ND5)
G4048A(ND1)
G4113A(ND1)
T5105C(ND2)
T5162C(ND2)
A12523G(ND5)
C6164T(COX1)
G207A(HVS)
T279C(HVS)
T10908C(ND4)
A14782G(Cytb)
A15924G(Thr)
A16300G(HVS)
G749A(12s)
T2483C(16s)
G8998A(ATP6)
T7692C(COX2)
C5355T(ND2)
G13928C(ND5)
T16311C(HVS)
FJ544232
C10511T(ND4L)
HM036551
HM036557
16296.1C(HVS)
LB168
LB177
XEB056
XEB073
XEB075
XEB077
XEB004
XEB114
XEB125
XEB009
XEB023
XEB160
XEB161
A12634G(ND5)
C13478T(ND5)
T14798C(Cytb)
DR087
DR088
DR046
FJ748732
GQ895149
GQ895162
HM036532
DR047
LB193
GQ895147
XEB111
XEB131
XEB150
XEB013
XEB059
XEB062
XEB184
GQ895144
HM036574
DB008

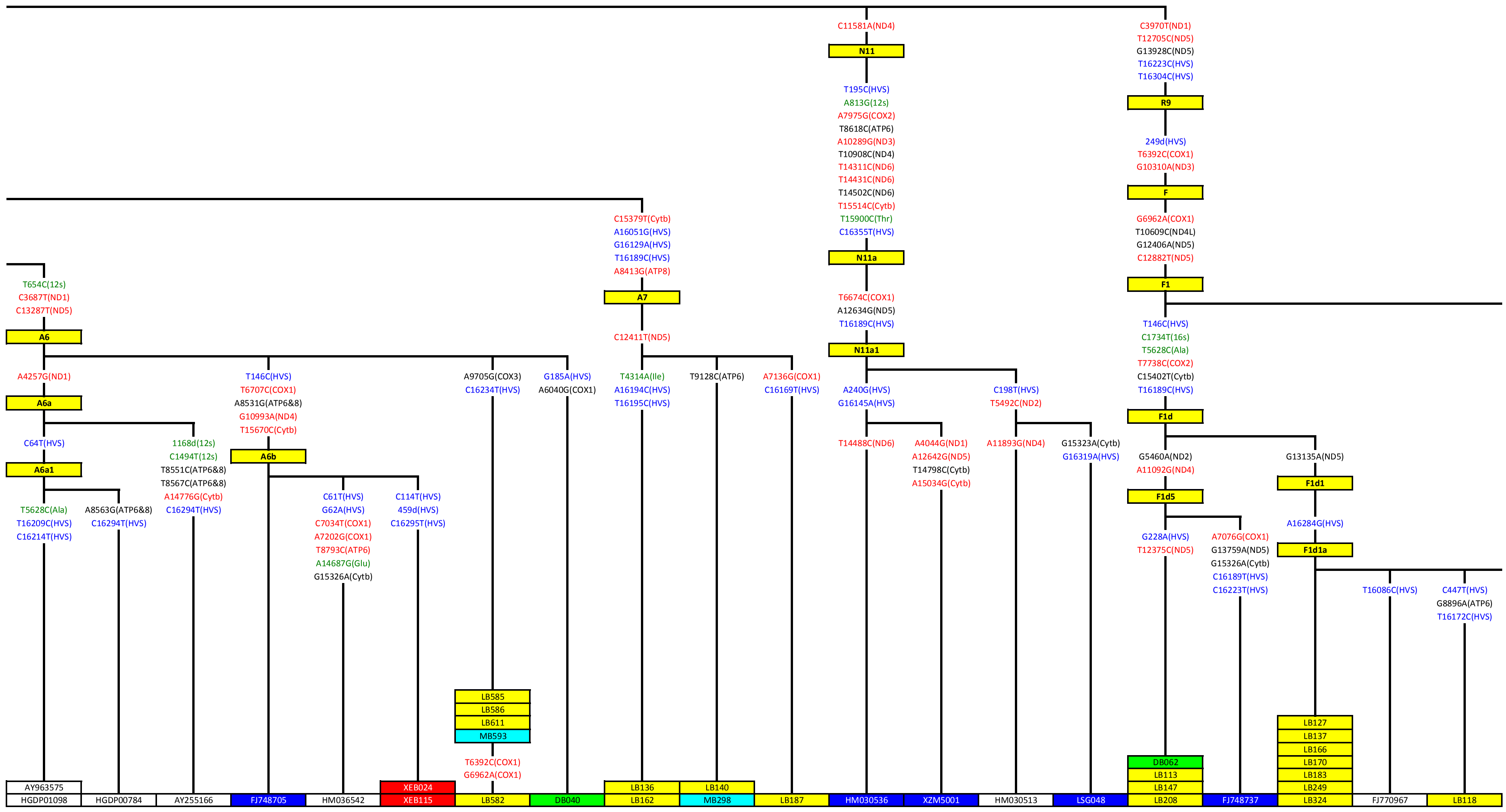

T654C(12s)
C3687T(ND1)
C13287T(ND5)
A6
A4257G(ND1)
A6a
C64T(HVS)
A6a1
T5628C(Ala)
T16209C(HVS)
C16214T(HVS)
A8563G(ATP6&8)
C16294T(HVS)
1168d(12s)
C1494T(12s)
T8551C(ATP6&8)
T8567C(ATP6&8)
A14776G(Cytb)
C16294T(HVS)
T146C(HVS)
T6707C(COX1)
A8531G(ATP6&8)
G10993A(ND4)
T15670C(Cytb)
A6b
C61T(HVS)
G62A(HVS)
C7034T(COX1)
A7202G(COX1)
T8793C(ATP6)
A14687G(Glu)
G15326A(Cytb)
C114T(HVS)
459d(HVS)
C16295T(HVS)
A9705G(COX3)
C16234T(HVS)
LB585
LB586
LB611
MB593
T6392C(COX1)
G6962A(COX1)
G185A(HVS)
A6040G(COX1)
C15379T(Cytb)
A16051G(HVS)
G16129A(HVS)
T16189C(HVS)
A8413G(ATP8)
A7
C12411T(ND5)
T4314A(Ile)
A16194C(HVS)
T16195C(HVS)
T9128C(ATP6)
A7136G(COX1)
C16169T(HVS)
C11581A(ND4)
N11
T195C(HVS)
A813G(12s)
A7975G(COX2)
T8618C(ATP6)
A10289G(ND3)
T10908C(ND4)
T14311C(ND6)
T14431C(ND6)
T14502C(ND6)
T15514C(Cytb)
T15900C(Thr)
C16355T(HVS)
N11a
T6674C(COX1)
A12634G(ND5)
T16189C(HVS)
N11a1
A240G(HVS)
G16145A(HVS)
T14488C(ND6)
A4044G(ND1)
A12642G(ND5)
T14798C(Cytb)
A15034G(Cytb)
C198T(HVS)
T5492C(ND2)
A11893G(ND4)
G15323A(Cytb)
G16319A(HVS)
C3970T(ND1)
T12705C(ND5)
G13928C(ND5)
T16223C(HVS)
T16304C(HVS)
R9
249d(HVS)
T6392C(COX1)
G10310A(ND3)
F
G6962A(COX1)
T10609C(ND4L)
G12406A(ND5)
C12882T(ND5)
F1
T146C(HVS)
C1734T(16s)
T5628C(Ala)
T7738C(COX2)
C15402T(Cytb)
T16189C(HVS)
F1d
G5460A(ND2)
A11092G(ND4)
F1d5
G228A(HVS)
T12375C(ND5)
A7076G(COX1)
G13759A(ND5)
G15326A(Cytb)
C16189T(HVS)
C16223T(HVS)
G13135A(ND5)
F1d1
A16284G(HVS)
F1d1a
T16086C(HVS)
C447T(HVS)
G8896A(ATP6)
T16172C(HVS)
LB127
LB137
LB166
LB170
LB183
LB249
DB062
LB113
LB147
AY963575
HGDP01098
HGDP00784
AY255166
FJ748705
HM036542
XEB024
XEB115
LB582
DB040
LB136
LB162
LB140
MB298
LB187
HM030536
XZM5001
HM030513
LSG048
LB208
FJ748737
LB324
FJ770967
LB118

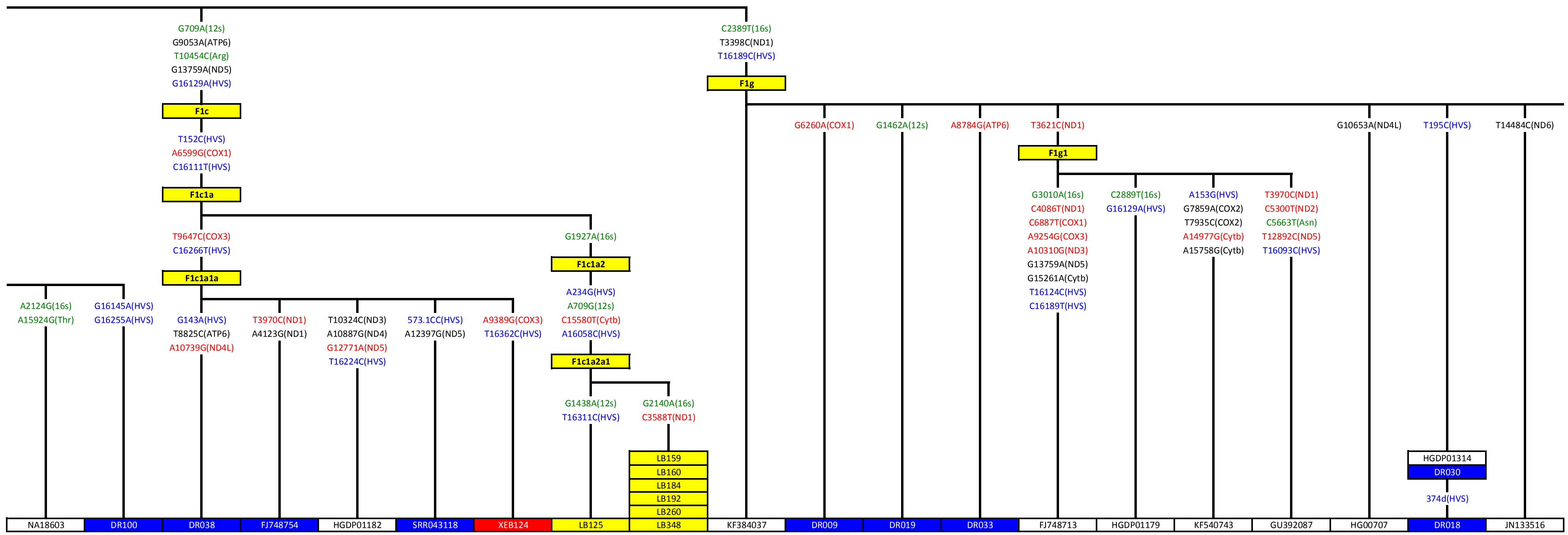
G709A(12s)
G9053A(ATP6)
T10454C(Arg)
G13759A(ND5)
G16129A(HVS)
F1c
T152C(HVS)
A6599G(COX1)
C16111T(HVS)
F1c1a
T9647C(COX3)
C16266T(HVS)
F1c1a1a
G1927A(16s)
F1c1a2
A234G(HVS)
A709G(12s)
C15580T(Cytb)
A16058C(HVS)
F1c1a2a1
G1438A(12s)
T16311C(HVS)
G2140A(16s)
C3588T(ND1)
A2124G(16s)
A15924G(Thr)
G16145A(HVS)
G16255A(HVS)
G143A(HVS)
T8825C(ATP6)
A10739G(ND4L)
T3970C(ND1)
A4123G(ND1)
T10324C(ND3)
A10887G(ND4)
G12771A(ND5)
T16224C(HVS)
573.1CC(HVS)
A12397G(ND5)
A9389G(COX3)
T16362C(HVS)
C2389T(16s)
T3398C(ND1)
T16189C(HVS)
F1g
G6260A(COX1)
G1462A(12s)
A8784G(ATP6)
T3621C(ND1)
F1g1
G3010A(16s)
C4086T(ND1)
C6887T(COX1)
A9254G(COX3)
A10310G(ND3)
G13759A(ND5)
G15261A(Cytb)
T16124C(HVS)
C16189T(HVS)
C2889T(16s)
G16129A(HVS)
A153G(HVS)
G7859A(COX2)
T7935C(COX2)
A14977G(Cytb)
A15758G(Cytb)
T3970C(ND1)
C5300T(ND2)
C5663T(Asn)
T12892C(ND5)
T16093C(HVS)
G10653A(ND4L)
T195C(HVS)
T14484C(ND6)
HGDP01314
DR030
374d(HVS)
LB159
LB160
LB184
LB192
LB260
NA18603
DR100
DR038
FJ748754
HGDP01182
SRR043118
XEB124
LB125
LB348
KF384037
DR009
DR019
DR033
FJ748713
HGDP01179
KF540743
GU392087
HG00707
DR018
JN133516

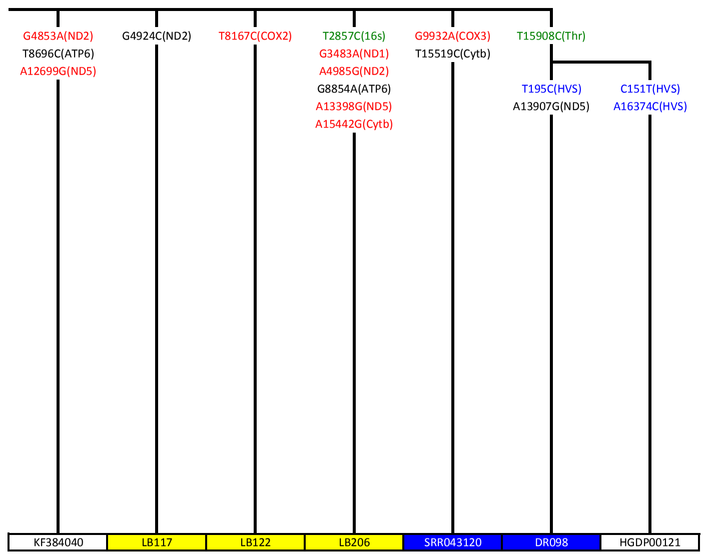
G4853A(ND2)
T8696C(ATP6)
A12699G(ND5)
G4924C(ND2)
T8167C(COX2)
T2857C(16s)
G3483A(ND1)
A4985G(ND2)
G8854A(ATP6)
A13398G(ND5)
A15442G(Cytb)
G9932A(COX3)
T15519C(Cytb)
T15908C(Thr)
T195C(HVS)
A13907G(ND5)
C151T(HVS)
A16374C(HVS)
KF384040
LB117
LB122
LB206
SRR043120
DR098
HGDP00121

## Figure S5 Models constructed for simulations

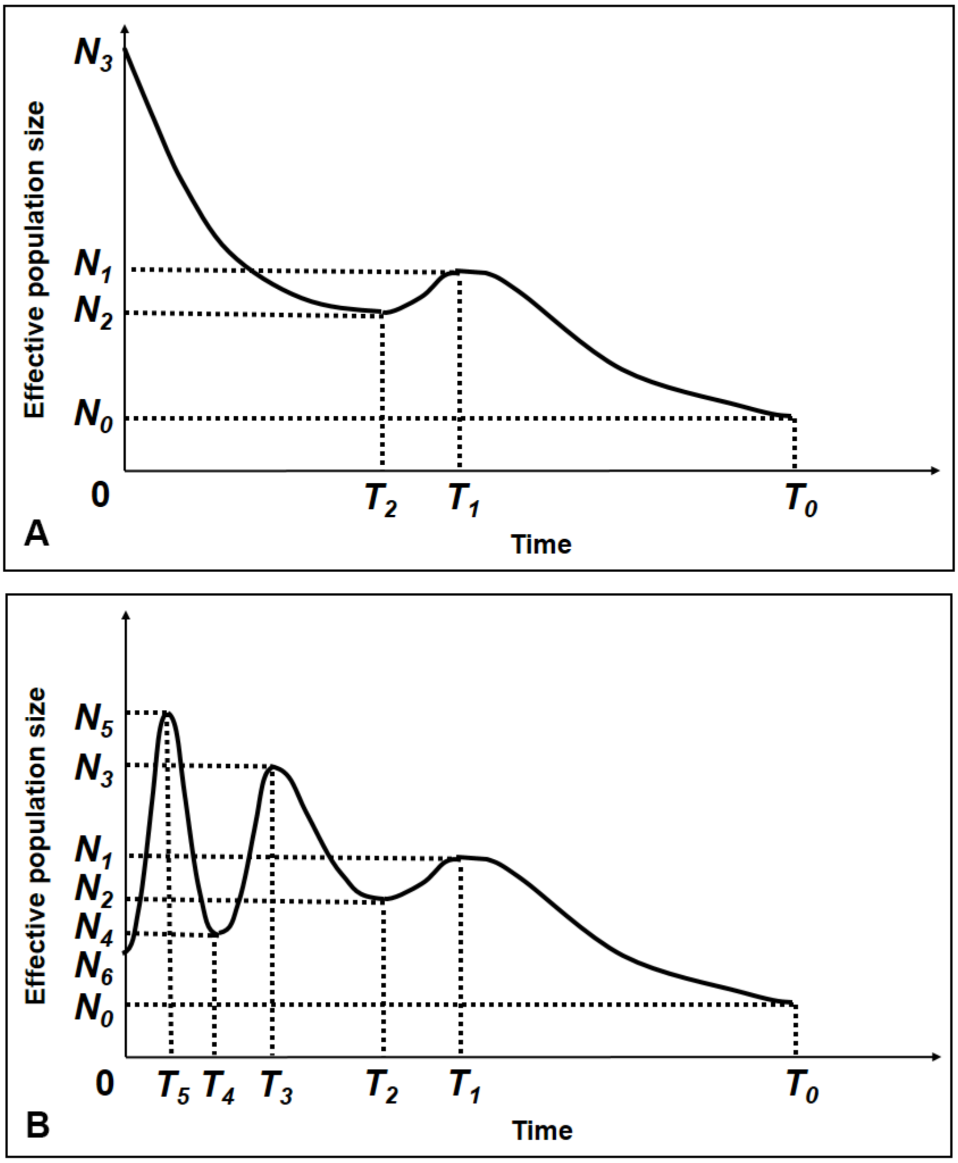


Note: According to the BSPs of the 367 East Asians from 1000 Genomes Projects and 432 Tibetan highlanders, we constructed demographic models for both populations. The ancestors of East Asian began to expand at $T_0$ (50 kya) with population size $N_0$ (1,000), then ended expansion at $T_1$ (25 kya) with population size $N_1$ (20,000). The population size of East Asian further decreased to $N_2$ (14,000) until $T_2$ (17.5 kya), then expanded to $N_3$. $N_3$ was set 400,000, 800,000 and 1,200,000 in East Asian Model 1, 2 and 3, respectively. The models for Tibetan highlanders were similar to those of East Asians until $T_2$. Then, highlanders expanded to $N_3$ (40,000) at $T_3$ (10 kya), decreased to

$N_4$ (12,000) at $T_4$ (5 kya), further expanded to $N_5$ (120,000) at $T_5$ (2.5 kya), and finally ended in the bottleneck with $N_6$. $N_6$ was set 4,000, 8,000 and 12,000 in Tibetan highlander Model 1, 2 and 3, respectively to check the different bottleneck effect. The population size changed exponentially. In total, 18 situations were simulated.

## Figure S6 Probabilistic distribution of founder clusters in Sherpas, Dengs, Lhobas and Monpas

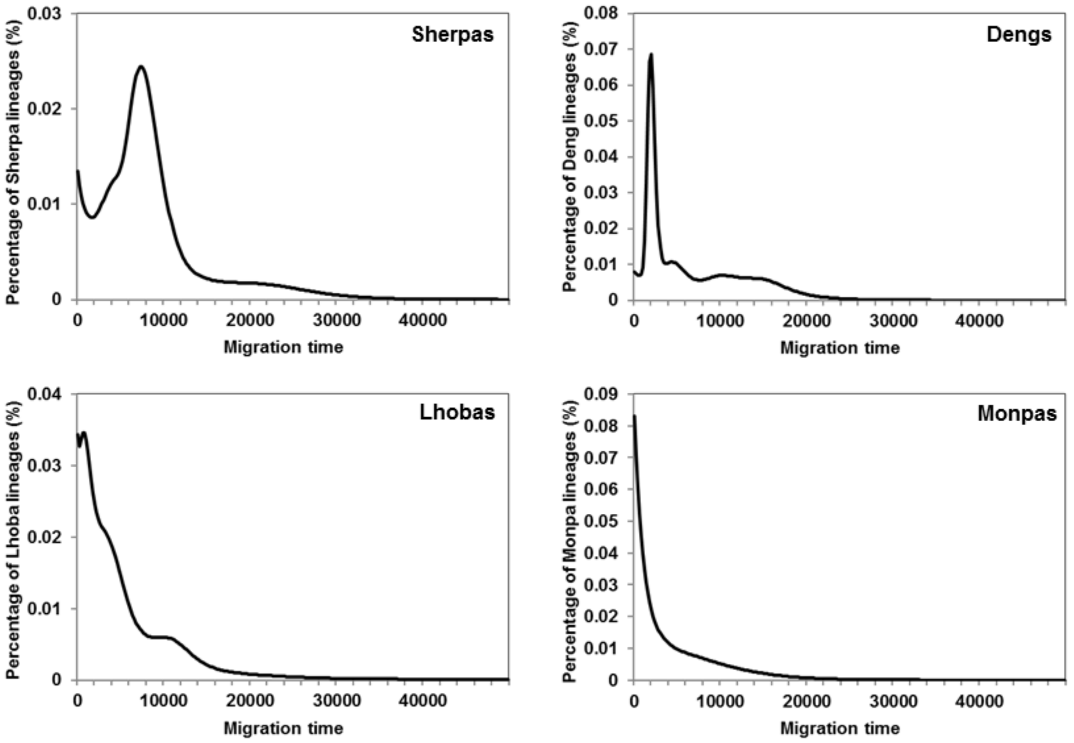


Note: Probabilistic distribution of founder clusters in 4 highlander populations across migration times scanned at 200-year intervals from 0 to 50 kya, using the $f_0$ criterion.

## Table S1. Sample information of 549 Tibetan highlanders

| ID | ethnic group | source |
|---|---|---|
| FJ383181 | Dirang Monpa | Chandrasekar et al. 2009 [7] |
| FJ383182 | Dirang Monpa | Chandrasekar et al. 2009 |
| FJ383294 | Dirang Monpa | Chandrasekar et al. 2009 |
| FJ383324 | Dirang Monpa | Chandrasekar et al. 2009 |
| FJ383325 | Dirang Monpa | Chandrasekar et al. 2009 |
| FJ383392 | Dirang Monpa | Chandrasekar et al. 2009 |
| FJ383428 | Dirang Monpa | Chandrasekar et al. 2009 |
| FJ383501 | Dirang Monpa | Chandrasekar et al. 2009 |
| FJ383502 | Dirang Monpa | Chandrasekar et al. 2009 |
| FJ383512 | Dirang Monpa | Chandrasekar et al. 2009 |
| FJ383620 | Dirang Monpa | Chandrasekar et al. 2009 |
| FJ383621 | Dirang Monpa | Chandrasekar et al. 2009 |
| FJ383622 | Dirang Monpa | Chandrasekar et al. 2009 |
| FJ383623 | Dirang Monpa | Chandrasekar et al. 2009 |
| FJ383624 | Dirang Monpa | Chandrasekar et al. 2009 |
| FJ383625 | Dirang Monpa | Chandrasekar et al. 2009 |
| FJ383626 | Dirang Monpa | Chandrasekar et al. 2009 |
| FJ383627 | Dirang Monpa | Chandrasekar et al. 2009 |
| FJ383628 | Dirang Monpa | Chandrasekar et al. 2009 |
| FJ383629 | Dirang Monpa | Chandrasekar et al. 2009 |
| FJ383723 | Dirang Monpa | Chandrasekar et al. 2009 |
| FJ383725 | Dirang Monpa | Chandrasekar et al. 2009 |
| FJ383726 | Dirang Monpa | Chandrasekar et al. 2009 |
| FJ383727 | Dirang Monpa | Chandrasekar et al. 2009 |
| FJ383728 | Dirang Monpa | Chandrasekar et al. 2009 |
| FJ383729 | Dirang Monpa | Chandrasekar et al. 2009 |
| FJ383730 | Dirang Monpa | Chandrasekar et al. 2009 |
| FJ383731 | Dirang Monpa | Chandrasekar et al. 2009 |
| FJ383732 | Dirang Monpa | Chandrasekar et al. 2009 |
| FJ383733 | Dirang Monpa | Chandrasekar et al. 2009 |
| FJ383734 | Dirang Monpa | Chandrasekar et al. 2009 |

| | | |
|---|---|---|
| FJ383769 | Dirang Monpa | Chandrasekar et al. 2009 |
| FJ748704 | Tibetan | Ji et al. 2012 [3] |
| FJ748705 | Tibetan | Ji et al. 2012 |
| FJ748716 | Tibetan | Ji et al. 2012 |
| FJ748717 | Tibetan | Ji et al. 2012 |
| FJ748718 | Tibetan | Ji et al. 2012 |
| FJ748726 | Tibetan | Ji et al. 2012 |
| FJ748727 | Tibetan | Ji et al. 2012 |
| FJ748728 | Tibetan | Ji et al. 2012 |
| FJ748729 | Tibetan | Ji et al. 2012 |
| FJ748730 | Tibetan | Ji et al. 2012 |
| FJ748731 | Tibetan | Ji et al. 2012 |
| FJ748732 | Tibetan | Ji et al. 2012 |
| FJ748733 | Tibetan | Ji et al. 2012 |
| FJ748734 | Tibetan | Ji et al. 2012 |
| FJ748735 | Tibetan | Ji et al. 2012 |
| FJ748736 | Tibetan | Ji et al. 2012 |
| FJ748737 | Tibetan | Ji et al. 2012 |
| FJ748738 | Tibetan | Ji et al. 2012 |
| FJ748739 | Tibetan | Ji et al. 2012 |
| FJ748740 | Tibetan | Ji et al. 2012 |
| FJ748741 | Tibetan | Ji et al. 2012 |
| FJ748742 | Tibetan | Ji et al. 2012 |
| FJ748743 | Tibetan | Ji et al. 2012 |
| FJ748744 | Tibetan | Ji et al. 2012 |
| FJ748745 | Tibetan | Ji et al. 2012 |
| FJ748746 | Tibetan | Ji et al. 2012 |
| FJ748747 | Tibetan | Ji et al. 2012 |
| FJ748748 | Tibetan | Ji et al. 2012 |
| FJ748749 | Tibetan | Ji et al. 2012 |
| FJ748750 | Tibetan | Ji et al. 2012 |
| FJ748751 | Tibetan | Ji et al. 2012 |
| FJ748752 | Tibetan | Ji et al. 2012 |
| FJ748753 | Tibetan | Ji et al. 2012 |
| FJ748754 | Tibetan | Ji et al. 2012 |

| | | |
|---|---|---|
| FJ748755 | Tibetan | Ji et al. 2012 |
| FJ748756 | Tibetan | Ji et al. 2012 |
| FJ748758 | Tibetan | Ji et al. 2012 |
| FJ748759 | Tibetan | Ji et al. 2012 |
| XEB002 | Sherpa | Kang et al. 2013 [6] |
| XEB003 | Sherpa | Kang et al. 2013 |
| XEB004 | Sherpa | Kang et al. 2013 |
| XEB005 | Sherpa | Kang et al. 2013 |
| XEB006 | Sherpa | Kang et al. 2013 |
| XEB008 | Sherpa | Kang et al. 2013 |
| XEB009 | Sherpa | Kang et al. 2013 |
| XEB012 | Sherpa | Kang et al. 2013 |
| XEB013 | Sherpa | Kang et al. 2013 |
| XEB014 | Sherpa | Kang et al. 2013 |
| XEB015 | Sherpa | Kang et al. 2013 |
| XEB017 | Sherpa | Kang et al. 2013 |
| XEB020 | Sherpa | Kang et al. 2013 |
| XEB023 | Sherpa | Kang et al. 2013 |
| XEB024 | Sherpa | Kang et al. 2013 |
| XEB026 | Sherpa | Kang et al. 2013 |
| XEB029 | Sherpa | Kang et al. 2013 |
| XEB031 | Sherpa | Kang et al. 2013 |
| XEB035 | Sherpa | Kang et al. 2013 |
| XEB036 | Sherpa | Kang et al. 2013 |
| XEB039 | Sherpa | Kang et al. 2013 |
| XEB042 | Sherpa | Kang et al. 2013 |
| XEB043 | Sherpa | Kang et al. 2013 |
| XEB047 | Sherpa | Kang et al. 2013 |
| XEB048 | Sherpa | Kang et al. 2013 |
| XEB049 | Sherpa | Kang et al. 2013 |
| XEB050 | Sherpa | Kang et al. 2013 |
| XEB051 | Sherpa | Kang et al. 2013 |
| XEB056 | Sherpa | Kang et al. 2013 |
| XEB057 | Sherpa | Kang et al. 2013 |
| XEB058 | Sherpa | Kang et al. 2013 |

| | | |
|---|---|---|
| XEB059 | Sherpa | Kang et al. 2013 |
| XEB062 | Sherpa | Kang et al. 2013 |
| XEB072 | Sherpa | Kang et al. 2013 |
| XEB073 | Sherpa | Kang et al. 2013 |
| XEB075 | Sherpa | Kang et al. 2013 |
| XEB076 | Sherpa | Kang et al. 2013 |
| XEB077 | Sherpa | Kang et al. 2013 |
| XEB098 | Sherpa | Kang et al. 2013 |
| XEB100 | Sherpa | Kang et al. 2013 |
| XEB101 | Sherpa | Kang et al. 2013 |
| XEB102 | Sherpa | Kang et al. 2013 |
| XEB103 | Sherpa | Kang et al. 2013 |
| XEB104 | Sherpa | Kang et al. 2013 |
| XEB108 | Sherpa | Kang et al. 2013 |
| XEB110 | Sherpa | Kang et al. 2013 |
| XEB111 | Sherpa | Kang et al. 2013 |
| XEB113 | Sherpa | Kang et al. 2013 |
| XEB114 | Sherpa | Kang et al. 2013 |
| XEB115 | Sherpa | Kang et al. 2013 |
| XEB116 | Sherpa | Kang et al. 2013 |
| XEB124 | Sherpa | Kang et al. 2013 |
| XEB125 | Sherpa | Kang et al. 2013 |
| XEB126 | Sherpa | Kang et al. 2013 |
| XEB129 | Sherpa | Kang et al. 2013 |
| XEB130 | Sherpa | Kang et al. 2013 |
| XEB131 | Sherpa | Kang et al. 2013 |
| XEB139 | Sherpa | Kang et al. 2013 |
| XEB145 | Sherpa | Kang et al. 2013 |
| XEB149 | Sherpa | Kang et al. 2013 |
| XEB150 | Sherpa | Kang et al. 2013 |
| XEB151 | Sherpa | Kang et al. 2013 |
| XEB152 | Sherpa | Kang et al. 2013 |
| XEB158 | Sherpa | Kang et al. 2013 |
| XEB160 | Sherpa | Kang et al. 2013 |
| XEB161 | Sherpa | Kang et al. 2013 |

| | | |
|---|---|---|
| XEB162 | Sherpa | Kang et al. 2013 |
| XEB163 | Sherpa | Kang et al. 2013 |
| XEB164 | Sherpa | Kang et al. 2013 |
| XEB166 | Sherpa | Kang et al. 2013 |
| XEB170 | Sherpa | Kang et al. 2013 |
| XEB171 | Sherpa | Kang et al. 2013 |
| XEB174 | Sherpa | Kang et al. 2013 |
| XEB180 | Sherpa | Kang et al. 2013 |
| XEB184 | Sherpa | Kang et al. 2013 |
| XEB185 | Sherpa | Kang et al. 2013 |
| DB001 | Deng | the current study |
| DB003 | Deng | the current study |
| DB004 | Deng | the current study |
| DB006 | Deng | the current study |
| DB007 | Deng | the current study |
| DB008 | Deng | the current study |
| DB009 | Deng | the current study |
| DB010 | Deng | the current study |
| DB011 | Deng | the current study |
| DB012 | Deng | the current study |
| DB013 | Deng | the current study |
| DB014 | Deng | the current study |
| DB015 | Deng | the current study |
| DB016 | Deng | the current study |
| DB017 | Deng | the current study |
| DB018 | Deng | the current study |
| DB019 | Deng | the current study |
| DB020 | Deng | the current study |
| DB021 | Deng | the current study |
| DB022 | Deng | the current study |
| DB023 | Deng | the current study |
| DB024 | Deng | the current study |
| DB025 | Deng | the current study |
| DB027 | Deng | the current study |
| DB030 | Deng | the current study |

| | | |
|---|---|---|
| DB031 | Deng | the current study |
| DB032 | Deng | the current study |
| DB033 | Deng | the current study |
| DB034 | Deng | the current study |
| DB035 | Deng | the current study |
| DB036 | Deng | the current study |
| DB037 | Deng | the current study |
| DB038 | Deng | the current study |
| DB039 | Deng | the current study |
| DB040 | Deng | the current study |
| DB041 | Deng | the current study |
| DB043 | Deng | the current study |
| DB044 | Deng | the current study |
| DB045 | Deng | the current study |
| DB046 | Deng | the current study |
| DB047 | Deng | the current study |
| DB048 | Deng | the current study |
| DB049 | Deng | the current study |
| DB050 | Deng | the current study |
| DB051 | Deng | the current study |
| DB052 | Deng | the current study |
| DB053 | Deng | the current study |
| DB054 | Deng | the current study |
| DB055 | Deng | the current study |
| DB056 | Deng | the current study |
| DB057 | Deng | the current study |
| DB058 | Deng | the current study |
| DB059 | Deng | the current study |
| DB060 | Deng | the current study |
| DB061 | Deng | the current study |
| DB062 | Deng | the current study |
| DB063 | Deng | the current study |
| DB064 | Deng | the current study |
| DB065 | Deng | the current study |
| DB066 | Deng | the current study |

| | | |
|---|---|---|
| DB067 | Deng | the current study |
| DB068 | Deng | the current study |
| DB069 | Deng | the current study |
| DB070 | Deng | the current study |
| DB071 | Deng | the current study |
| DB072 | Deng | the current study |
| DB073 | Deng | the current study |
| DB074 | Deng | the current study |
| DB075 | Deng | the current study |
| DB076 | Deng | the current study |
| DB077 | Deng | the current study |
| DB079 | Deng | the current study |
| DB080 | Deng | the current study |
| DB081 | Deng | the current study |
| DB083 | Deng | the current study |
| DB084 | Deng | the current study |
| DB086 | Deng | the current study |
| DB087 | Deng | the current study |
| DB088 | Deng | the current study |
| DB089 | Deng | the current study |
| DB092 | Deng | the current study |
| DB093 | Deng | the current study |
| DB094 | Deng | the current study |
| DB095 | Deng | the current study |
| DB096 | Deng | the current study |
| DB097 | Deng | the current study |
| DB098 | Deng | the current study |
| DB099 | Deng | the current study |
| DB100 | Deng | the current study |
| DB101 | Deng | the current study |
| DB102 | Deng | the current study |
| DR001 | Tingri,Tibetan | the current study |
| DR002 | Tingri,Tibetan | the current study |
| DR004 | Tingri,Tibetan | the current study |
| DR005 | Tingri,Tibetan | the current study |

| | | |
|---|---|---|
| DR006 | Tingri,Tibetan | the current study |
| DR007 | Tingri,Tibetan | the current study |
| DR008 | Tingri,Tibetan | the current study |
| DR009 | Tingri,Tibetan | the current study |
| DR010 | Tingri,Tibetan | the current study |
| DR012 | Tingri,Tibetan | the current study |
| DR013 | Tingri,Tibetan | the current study |
| DR014 | Tingri,Tibetan | the current study |
| DR015 | Tingri,Tibetan | the current study |
| DR016 | Tingri,Tibetan | the current study |
| DR017 | Tingri,Tibetan | the current study |
| DR018 | Tingri,Tibetan | the current study |
| DR019 | Tingri,Tibetan | the current study |
| DR020 | Tingri,Tibetan | the current study |
| DR021 | Tingri,Tibetan | the current study |
| DR022 | Tingri,Tibetan | the current study |
| DR023 | Tingri,Tibetan | the current study |
| DR024 | Tingri,Tibetan | the current study |
| DR025 | Tingri,Tibetan | the current study |
| DR026 | Tingri,Tibetan | the current study |
| DR027 | Tingri,Tibetan | the current study |
| DR028 | Tingri,Tibetan | the current study |
| DR029 | Tingri,Tibetan | the current study |
| DR030 | Tingri,Tibetan | the current study |
| DR031 | Tingri,Tibetan | the current study |
| DR032 | Tingri,Tibetan | the current study |
| DR033 | Tingri,Tibetan | the current study |
| DR034 | Tingri,Tibetan | the current study |
| DR036 | Tingri,Tibetan | the current study |
| DR037 | Tingri,Tibetan | the current study |
| DR038 | Tingri,Tibetan | the current study |
| DR039 | Tingri,Tibetan | the current study |
| DR040 | Tingri,Tibetan | the current study |
| DR041 | Tingri,Tibetan | the current study |
| DR042 | Tingri,Tibetan | the current study |

| | | |
|---|---|---|
| DR043 | Tingri,Tibetan | the current study |
| DR044 | Tingri,Tibetan | the current study |
| DR045 | Tingri,Tibetan | the current study |
| DR046 | Tingri,Tibetan | the current study |
| DR047 | Tingri,Tibetan | the current study |
| DR048 | Tingri,Tibetan | the current study |
| DR049 | Tingri,Tibetan | the current study |
| DR050 | Tingri,Tibetan | the current study |
| DR051 | Tingri,Tibetan | the current study |
| DR052 | Tingri,Tibetan | the current study |
| DR055 | Tingri,Tibetan | the current study |
| DR058 | Tingri,Tibetan | the current study |
| DR060 | Tingri,Tibetan | the current study |
| DR062 | Tingri,Tibetan | the current study |
| DR066 | Tingri,Tibetan | the current study |
| DR069 | Tingri,Tibetan | the current study |
| DR070 | Tingri,Tibetan | the current study |
| DR071 | Tingri,Tibetan | the current study |
| DR072 | Tingri,Tibetan | the current study |
| DR073 | Tingri,Tibetan | the current study |
| DR074 | Tingri,Tibetan | the current study |
| DR075 | Tingri,Tibetan | the current study |
| DR076 | Tingri,Tibetan | the current study |
| DR077 | Tingri,Tibetan | the current study |
| DR078 | Tingri,Tibetan | the current study |
| DR079 | Tingri,Tibetan | the current study |
| DR080 | Tingri,Tibetan | the current study |
| DR081 | Tingri,Tibetan | the current study |
| DR082 | Tingri,Tibetan | the current study |
| DR083 | Tingri,Tibetan | the current study |
| DR084 | Tingri,Tibetan | the current study |
| DR085 | Tingri,Tibetan | the current study |
| DR086 | Tingri,Tibetan | the current study |
| DR087 | Tingri,Tibetan | the current study |
| DR088 | Tingri,Tibetan | the current study |

| | | |
|---|---|---|
| DR090 | Tingri,Tibetan | the current study |
| DR092 | Tingri,Tibetan | the current study |
| DR094 | Tingri,Tibetan | the current study |
| DR098 | Tingri,Tibetan | the current study |
| DR100 | Tingri,Tibetan | the current study |
| DR101 | Tingri,Tibetan | the current study |
| DR102 | Tingri,Tibetan | the current study |
| DR103 | Tingri,Tibetan | the current study |
| DR105 | Tingri,Tibetan | the current study |
| DR106 | Tingri,Tibetan | the current study |
| DR109 | Tingri,Tibetan | the current study |
| DR110 | Tingri,Tibetan | the current study |
| LB109 | Lhoba | the current study |
| LB110 | Lhoba | the current study |
| LB111 | Lhoba | the current study |
| LB112 | Lhoba | the current study |
| LB113 | Lhoba | the current study |
| LB114 | Lhoba | the current study |
| LB115 | Lhoba | the current study |
| LB117 | Lhoba | the current study |
| LB118 | Lhoba | the current study |
| LB119 | Lhoba | the current study |
| LB120 | Lhoba | the current study |
| LB121 | Lhoba | the current study |
| LB122 | Lhoba | the current study |
| LB124 | Lhoba | the current study |
| LB125 | Lhoba | the current study |
| LB127 | Lhoba | the current study |
| LB136 | Lhoba | the current study |
| LB137 | Lhoba | the current study |
| LB138 | Lhoba | the current study |
| LB139 | Lhoba | the current study |
| LB140 | Lhoba | the current study |
| LB141 | Lhoba | the current study |
| LB142 | Lhoba | the current study |

| | | |
|---|---|---|
| LB143 | Lhoba | the current study |
| LB147 | Lhoba | the current study |
| LB154 | Lhoba | the current study |
| LB155 | Lhoba | the current study |
| LB156 | Lhoba | the current study |
| LB159 | Lhoba | the current study |
| LB160 | Lhoba | the current study |
| LB162 | Lhoba | the current study |
| LB163 | Lhoba | the current study |
| LB164 | Lhoba | the current study |
| LB166 | Lhoba | the current study |
| LB167 | Lhoba | the current study |
| LB168 | Lhoba | the current study |
| LB169 | Lhoba | the current study |
| LB170 | Lhoba | the current study |
| LB171 | Lhoba | the current study |
| LB175 | Lhoba | the current study |
| LB176 | Lhoba | the current study |
| LB177 | Lhoba | the current study |
| LB180 | Lhoba | the current study |
| LB181 | Lhoba | the current study |
| LB183 | Lhoba | the current study |
| LB184 | Lhoba | the current study |
| LB185 | Lhoba | the current study |
| LB186 | Lhoba | the current study |
| LB187 | Lhoba | the current study |
| LB188 | Lhoba | the current study |
| LB189 | Lhoba | the current study |
| LB190 | Lhoba | the current study |
| LB191 | Lhoba | the current study |
| LB192 | Lhoba | the current study |
| LB193 | Lhoba | the current study |
| LB195 | Lhoba | the current study |
| LB197 | Lhoba | the current study |
| LB198 | Lhoba | the current study |

| | | |
|---|---|---|
| LB199 | Lhoba | the current study |
| LB200 | Lhoba | the current study |
| LB202 | Lhoba | the current study |
| LB203 | Lhoba | the current study |
| LB204 | Lhoba | the current study |
| LB206 | Lhoba | the current study |
| LB207 | Lhoba | the current study |
| LB208 | Lhoba | the current study |
| LB221 | Lhoba | the current study |
| LB223 | Lhoba | the current study |
| LB228 | Lhoba | the current study |
| LB232 | Lhoba | the current study |
| LB244 | Lhoba | the current study |
| LB249 | Lhoba | the current study |
| LB250 | Lhoba | the current study |
| LB251 | Lhoba | the current study |
| LB258 | Lhoba | the current study |
| LB260 | Lhoba | the current study |
| LB282 | Lhoba | the current study |
| LB285 | Lhoba | the current study |
| LB288 | Lhoba | the current study |
| LB324 | Lhoba | the current study |
| LB327 | Lhoba | the current study |
| LB331 | Lhoba | the current study |
| LB344 | Lhoba | the current study |
| LB345 | Lhoba | the current study |
| LB346 | Lhoba | the current study |
| LB348 | Lhoba | the current study |
| LB577 | Lhoba | the current study |
| LB582 | Lhoba | the current study |
| LB585 | Lhoba | the current study |
| LB586 | Lhoba | the current study |
| LB611 | Lhoba | the current study |
| MB298 | Monpa | the current study |
| MB328 | Monpa | the current study |

| | | |
|---|---|---|
| MB570 | Monpa | the current study |
| MB571 | Monpa | the current study |
| MB583 | Monpa | the current study |
| MB592 | Monpa | the current study |
| MB593 | Monpa | the current study |
| MB594 | Monpa | the current study |
| MB595 | Monpa | the current study |
| MB597 | Monpa | the current study |
| MB598 | Monpa | the current study |
| MB600 | Monpa | the current study |
| MB601 | Monpa | the current study |
| MB602 | Monpa | the current study |
| MB606 | Monpa | the current study |
| MB608 | Monpa | the current study |
| MB609 | Monpa | the current study |
| HM030526 | Tibetan | Kong et al. 2010 [8] |
| HM030529 | Tibetan | Kong et al. 2010 |
| HM030533 | Tibetan | Kong et al. 2010 |
| HM030534 | Tibetan | Kong et al. 2010 |
| HM030536 | Tibetan | Kong et al. 2010 |
| HM030537 | Tibetan | Kong et al. 2010 |
| HM030538 | Tibetan | Kong et al. 2010 |
| HM346894 | Tibetan | Peng et al. 2011 [9] |
| HM346897 | Tibetan | Peng et al. 2011 |
| HM346898 | Tibetan | Peng et al. 2011 |
| HM346902 | Lhoba | Peng et al. 2011 |
| HM346916 | Tibetan | Peng et al. 2011 |
| HM346917 | Tibetan | Peng et al. 2011 |
| HM346918 | Tibetan | Peng et al. 2011 |
| HM346919 | Tibetan | Peng et al. 2011 |
| HM346920 | Tibetan | Peng et al. 2011 |
| HM346921 | Tibetan | Peng et al. 2011 |
| HM346922 | Tibetan | Peng et al. 2011 |
| HM346923 | Tibetan | Peng et al. 2011 |
| HM346924 | Monpa | Peng et al. 2011 |

| | | |
|---|---|---|
| HM346925 | Monpa | Peng et al. 2011 |
| HM346926 | Tibetan | Peng et al. 2011 |
| HM346927 | Tibetan | Peng et al. 2011 |
| HM346928 | Tibetan | Peng et al. 2011 |
| HM346930 | Tibetan | Peng et al. 2011 |
| HM346931 | Tibetan | Peng et al. 2011 |
| HM346936 | Tibetan | Peng et al. 2011 |
| GQ895140 | Tibetan | Qin et al. 2010 [10] |
| GQ895141 | Tibetan | Qin et al. 2010 |
| GQ895142 | Tibetan | Qin et al. 2010 |
| GQ895143 | Tibetan | Qin et al. 2010 |
| GQ895144 | Tibetan | Qin et al. 2010 |
| GQ895145 | Tibetan | Qin et al. 2010 |
| GQ895146 | Tibetan | Qin et al. 2010 |
| GQ895147 | Tibetan | Qin et al. 2010 |
| GQ895148 | Tibetan | Qin et al. 2010 |
| GQ895149 | Tibetan | Qin et al. 2010 |
| GQ895150 | Tibetan | Qin et al. 2010 |
| GQ895151 | Tibetan | Qin et al. 2010 |
| GQ895152 | Tibetan | Qin et al. 2010 |
| GQ895153 | Tibetan | Qin et al. 2010 |
| GQ895154 | Tibetan | Qin et al. 2010 |
| GQ895155 | Tibetan | Qin et al. 2010 |
| GQ895156 | Tibetan | Qin et al. 2010 |
| GQ895157 | Tibetan | Qin et al. 2010 |
| GQ895158 | Tibetan | Qin et al. 2010 |
| GQ895159 | Tibetan | Qin et al. 2010 |
| GQ895160 | Tibetan | Qin et al. 2010 |
| GQ895161 | Tibetan | Qin et al. 2010 |
| GQ895162 | Tibetan | Qin et al. 2010 |
| GQ895163 | Tibetan | Qin et al. 2010 |
| GQ895164 | Tibetan | Qin et al. 2010 |
| GQ895165 | Tibetan | Qin et al. 2010 |
| GQ895166 | Tibetan | Qin et al. 2010 |
| GQ895167 | Tibetan | Qin et al. 2010 |

| | | |
|---|---|---|
| GQ895168 | Tibetan | Qin et al. 2010 |
| GQ895169 | Tibetan | Qin et al. 2010 |
| GQ895170 | Tibetan | Qin et al. 2010 |
| SRR043118 | Tibetan | Yi et al. 2011 [11] |
| SRR043119 | Tibetan | Yi et al. 2011 |
| SRR043120 | Tibetan | Yi et al. 2011 |
| SRR043121 | Tibetan | Yi et al. 2011 |
| SRR043122 | Tibetan | Yi et al. 2011 |
| SRR043124 | Tibetan | Yi et al. 2011 |
| SRR043126 | Tibetan | Yi et al. 2011 |
| SRR043128 | Tibetan | Yi et al. 2011 |
| SRR043129 | Tibetan | Yi et al. 2011 |
| SRR043130 | Tibetan | Yi et al. 2011 |
| SRR043131 | Tibetan | Yi et al. 2011 |
| SRR043132 | Tibetan | Yi et al. 2011 |
| SRR043133 | Tibetan | Yi et al. 2011 |
| SRR043135 | Tibetan | Yi et al. 2011 |
| SRR043136 | Tibetan | Yi et al. 2011 |
| SRR043137 | Tibetan | Yi et al. 2011 |
| SRR043138 | Tibetan | Yi et al. 2011 |
| SRR043140 | Tibetan | Yi et al. 2011 |
| SRR043141 | Tibetan | Yi et al. 2011 |
| SRR043142 | Tibetan | Yi et al. 2011 |
| SRR043143 | Tibetan | Yi et al. 2011 |
| SRR043144 | Tibetan | Yi et al. 2011 |
| SRR043145 | Tibetan | Yi et al. 2011 |
| SRR043146 | Tibetan | Yi et al. 2011 |
| SRR043147 | Tibetan | Yi et al. 2011 |
| SRR043148 | Tibetan | Yi et al. 2011 |
| SRR043149 | Tibetan | Yi et al. 2011 |
| SRR043150 | Tibetan | Yi et al. 2011 |
| SRR043151 | Tibetan | Yi et al. 2011 |
| SRR043152 | Tibetan | Yi et al. 2011 |
| SRR043153 | Tibetan | Yi et al. 2011 |
| SRR043154 | Tibetan | Yi et al. 2011 |

| | | |
|---|---|---|
| SRR043156 | Tibetan | Yi et al. 2011 |
| FJ544230 | Tibetan | Zhao et al. 2009 [12] |
| FJ544231 | Tibetan | Zhao et al. 2009 |
| FJ544232 | Tibetan | Zhao et al. 2009 |
| FJ544233 | Tibetan | Zhao et al. 2009 |
| FJ544235 | Tibetan | Zhao et al. 2009 |
| FJ544236 | Tibetan | Zhao et al. 2009 |
| FJ544237 | Tibetan | Zhao et al. 2009 |
| FJ544238 | Tibetan | Zhao et al. 2009 |
| FJ544239 | Tibetan | Zhao et al. 2009 |
| FJ544240 | Tibetan | Zhao et al. 2009 |
| FJ544241 | Tibetan | Zhao et al. 2009 |
| FJ968772 | Tibetan | Zhao et al. 2009 |
| FJ968773 | Tibetan | Zhao et al. 2009 |
| FJ968774 | Tibetan | Zhao et al. 2009 |
| GU014565 | Tibetan | Zhao et al. 2009 |
| GU014566 | Tibetan | Zhao et al. 2009 |
| GU014567 | Tibetan | Zhao et al. 2009 |
| GU014568 | Tibetan | Zhao et al. 2009 |
| GU014569 | Tibetan | Zhao et al. 2009 |
| ADG171 | Tibetan | The current study |
| ADG225 | Tibetan | The current study |
| BMG118 | Tibetan | The current study |
| CDG068 | Tibetan | The current study |
| CDG089 | Tibetan | The current study |
| CDG103 | Tibetan | The current study |
| LSG048 | Tibetan | The current study |
| XZM5001 | Tibetan | The current study |

## Table S2. 33 Tibetan mtDNA haplotypes compared to rSRS from fastq files of Yi et al.

| | |
|---|---|
| SRR043118 | C146T C195T A247G 249delA C305N (312-313)missing 315insC 573insC 573insC G709A A769G A825T A1018G A2758G C2885T T3594C C3970T G4104A T4312C T6392C A6599G G6962A G7146A T7256C A7521G A8096N T8468C T8655C G8701A G9053A (9482-9495)missing C9540T T9647C G10310A G10398A T10454C T10609C T10664C A10688G C10810T C10873T C10915T A11914G A12397G G12406A T12705C C12882T G13105A G13276A T13506C T13650C G13759A G13928C C16111T T16187C C16189T T16223C G16230A C16266T T16278C T16304C C16311T T16519C |
| SRR043119 | C146T C147N C152T C195T C198T A200G A215G A247G C269N (305-326)missing T489C A769G A825T A1018G T1095C (1244-1249)missing (1713-1719)missing T2193N A2214N (2225-2229)missing A2521N (2747-2767)missing C2885T (3164-3235)missing T3594C G4104A T4312C C5601N (5635-5646)missing (5942-5979)missing C5993N C6531T G6825N G7146A T7256C A7521G G7642N A8108G (8179-8185)missing T8468C T8655C T9950C (10026-10042)missing (10303-10306)missing C10400T T10664C A10688G C10810T C10915T A11914G G11969A A13074G G13105A G13276A T13506C T13650C C14340T T14783C G15043A G15301A (15904-15929)missing A16129G T16187C C16189T G16230A T16278C C16294T C16311T |
| SRR043120 | C146T C152T C195T A247G 249delA (312-313)missing 315insC (518-519)missing (748-750)missing A769G A825T A1018G (2230-2246)missing T2267N C2389T C2689N A2758G C2885T T3398C T3594C C3970T C4014N G4104A T4312C (4416-4438)missing T5580N C5713N T6392C G6962A T7028N G7146A T7256C A7521G T8468C T8655C G8701A C9540T G9932A G10310A G10398A T10609C |

| | |
|---|---|
| | T10664C A10688G C10810T C10873T C10915T A11914G G12406A T12705C C12882T G13105A G13276A T13506C T13650C G13928C T15519C A16129G A16183N T16187C T16223C G16230A T16278C T16304C C16311T T16519C |
| SRR043121 | C146T C152T C195T A247G (312-313)missing 315insC T489C G529N A769G A825T A1018G (2506-2525)missing A2758G C2885T G3010A A3537N T3594C G4104A T4135C T4312C C4883T C5178A G5262A (5741-5764)missing C6059N (7022-7024)missing G7146A T7256C A7521G T7581C T7783C C8414T T8468C T8655C G9053A C9348N C10400T T10539N T10664C A10688G C10810T C10915T G11696A A11914G T12130C A12358G G13105A G13276A T13506C T13650C C14668T T14783C (14928-14948)missing A14954N G15043A C15295T G15301A T16086C A16129G T16187C C16189T G16230A T16278C C16311T T16362C |
| SRR043122 | C146T C152T C195T A247G T489C G709A A769G A825T A1018G A2758G C2885T 3172insC T3594C G4104A C4140T T4312C (6027-6032)missing A6035N A6040N T6844N G7146A A7250G T7256C A7521G T8468C T8655C T8793C G8856A C10400T G10646A (10655-10661)missing T10664C A10688G C10810T C10915T A11914G C12549T G13105A A13152G G13276A T13506C T13650C T14502C T14783C C15040T G15043A T15071C A15218G G15301A C15913T A16066G A16129G T16187C C16189T G16230A T16278C |
| SRR043124 | C146T C152T C195T A247G T310C G316N T489C T711C A769G A825T A1018G A1041G C1182N A2758G C2885T T3394C T3594C A3601N G4104A T4312C G4491A G5744N A5747N 5899insC T7028N T7142C G7146A T7256C A7521G G7697A T8468C T8655C A9242G C10400T T10664C A10688G C10810T C10915T A11914G C12774A G13105A G13276A T13506C T13650C T14308C A14417G T14783C G15043A G15301A A16129G T16187C C16189T G16230A C16234T |

| | |
|---|---|
| | T16278C C16311T A16316G T16362C |
| SRR043126 | C146T C152T C195T (241-258)missing 315insC T489C A769G A825T A1018G C1395N C1715T 2232insA 2232insA A2758G C2885T T3552A C3576T T3594C G4104A T4312C A4715G A4884G A4958G (5582-5583)missing (5726-5763)missing G6026A G7146A C7196A T7256C A7521G T7999C T8468C A8508G G8584A T8655C A9545G C10400T T10664C A10688G C10810T C10915T G11969A A12672G G13105A A13263G G13276A T13506C T13650C T14318C T14783C G15043A T15204C G15301A A15487T T15968C T16093C T16187C C16189T G16230A T16278C T16298C C16311T C16327T T16519C |
| SRR043128 | C152T C195T A200G A247G 315insC T489C A769G A825T A1018G C1734T C2557N A2758G C2885T G3010A T3594C G4104A T4312C T4639C C4883T C5178A A7055G G7146A T7256C A7521G C8414T T8468C T8655C C10400T T10664C A10688G C10810T C10915T G11696A A11914G G13105A G13276A T13506C T13650C C14668T T14783C G15043A G15301A A16129G T16187C C16189T G16230A T16278C C16311T T16362C |
| SRR043129 | C146T C152T C195T A200G A247G T489C A769G A825T A1018G C1734T A2758G C2885T G3010A T3594C G4104A T4312C T4639C C4883T C5178A A7055G G7146A T7256C A7521G C8414T T8468C T8655C C10400T T10664C A10688G C10810T C10915T G11696A A11914G G13105A G13276A T13506C T13650C C14668T T14783C G15043A G15301A A16129G T16187C C16189T G16230A T16278C C16311T T16362C |
| SRR043130 | C146T C195T A235G A247G T252C (292-310)missing A663G A769G A825T T1005C A1018G A1736G A2758G C2885T T3594C G4104A T4248C T4312C A4824G 5899insC G6755A G7146A T7256C A7521G T8468C T8655C G8701A C8794T T8843C C9540T C9650T G10398A T10664C A10688G C10810T C10873T C10915T A11350G A11914G |

| | |
|---|---|
| | G13105A G13276A T13506C T13650C A16129G T16187C C16189T G16230A T16278C C16290T A16293C C16311T G16319A |
| SRR043131 | C146T C152T C195T C198N (246-315)missing T489C G709A A769G A825T C828N A1018G C1734N G2082N C2540N C2555N C2557N C2708N A2758G C2885T G3438A T3594C (3903-3922)missing G4104A T4312C A4833G T5108C (5505-5508)missing G5513N C5601T (5735-5759)missing G7146A T7256C A7521G G7600A C7840N (8141-8149)missing A8179N T8468C T8655C A9377N G9575A C9793N C10400T T10664C A10688G (10721-10722)missing C10810T C10915T A11719N A11914G C12281N C12861N (12874-12879)missing G13105A G13276A T13506C A13563G T13650C T14200C G14569A T14783C G14900N G15043A G15301A C15349N T16187C C16189T G16230A C16311T T16362C |
| SRR043132 | C146T C152T A247G 315insC T489C G709A A769G A825T A1018G A2758G C2885T T3594C G4104A T4312C C4796T A4833G T5108C G7146A A7245G T7256C A7521G T8468C T8655C G9123A C9599T C10400T T10664C A10688G C10810T C10915T A11914G G13105A G13276A G13477A T13506C T13650C T14215C G14569A A14605G T14783C G15043A G15301A G15927A A16129G T16187C C16189T G16230A G16274A T16278C T16362C |
| SRR043133 | C146T C195T T204C G207A A235G A247G G263A (303-305)missing (312-313)missing 315insC A663G A769G A825T A1018G A1736G T2352C A2758G C2885T T3594C G4104A T4248C T4312C A4824G G7146A T7256C A7521G A8459G T8468C T8655C G8701A C8794T C9540T G10398A T10664C A10688G C10810T C10873T C10915T A11084G A11914G G13105A G13276A T13506C T13650C C14067T G14569A G14831A A16129G T16187C C16189T G16230A G16274A T16278C C16290T C16311T G16319A T16362C |
| SRR043135 | C146T C152T C195T A247G 315insC T489C G499A C571G A769G |

| | |
|---|---|
| | A825T A1018G A2758G C2885T G3010A T3336C T3594C T3644C G4104A T4312C G4580A C4883T T5048C C5178A A6663G G7146A T7256C A7521G C8414T T8468C T8655C C9424T C10192T C10400T T10664C A10688G C10810T C10915T A11914G G13105A G13276A T13506C T13650C T13879C C14668T T14783C G15043A G15301A A16129G T16187C C16189T G16230A T16278C C16311T T16362C T16519C |
| SRR043136 | C146T C152T C195T (225-241)missing A247G T310C 315insC T489C A769G A825T A1018G T2118C C2502N A2758G C2885T G3010A T3594C G4104A T4312C C4883T C5178A G5262A G5769N (5799-5822)missing G5881N G7146A T7256C A7521G T7581C T7783C T7913N C8414T T8468C T8655C C10400T T10664C A10688G C10810T C10915T G11696A A11914G T12130C A12358G G13105A G13276A T13506C T13650C C14668T T14783C G15043A C15295T G15301A T16086C A16129G T16187C C16189T G16230A T16278C C16311T T16362C A16399G T16519C |
| SRR043137 | C64T T65A C146T C152T A237G A247G 315insC T489C C501T A769G A825T A1018G T1187C A2758G C2885T G3010A T3594C T3644C G4104A T4312C C4883T C5178A A5729N (5742-5747)missing G7146A T7256C A7521G C8414T T8468C T8655C C10400T C10527T T10664C A10688G C10810T C10915T A11884G A11914G G13105A G13276A T13506C T13650C C14668T T14783C G15043A G15301A C15625T G15884C T16093C A16129G T16187C C16189T G16230A T16278C C16294T C16311T T16362C |
| SRR043138 | (61-64)missing T65A C146T C152T A237G A247G C312N 315insC T489C C501T A769G A825T A1018G T1187C A2758G C2885T G3010A T3542N (3557-3559)missing T3594C T3644C G4104A T4312C C4883T C5178A T5709N (5713-5769)missing G7146A T7256C A7521G A8025N C8414T T8468C T8655C C10142N C10400T C10527T |

| | |
|---|---|
| | T10664C A10688G C10810T C10915T A11884G A11914G G13105A G13276A T13506C T13650C C14668T T14783C G15043A G15301A G15884C T16093C A16129G T16187C C16189T G16230A T16278C C16294T C16311T T16362C |
| SRR043140 | C146T C152T C195T A247G 315insC T489C T711C A769G A825T A1018G A1041G A2758G C2885T T3394C T3594C G4104A T4312C G4491A A5894C 5899insC T7142C G7146A T7256C A7521G G7697A T8468C T8655C A9242G C10400T T10664C A10688G C10810T C10915T A11914G G13105A G13276A T13506C T13650C T14308C A14417G T14783C G15043A G15301A A16129G T16187C C16189T G16230A C16234T T16278C C16311T A16316G T16362C |
| SRR043141 | C146T C150T A153G C195T A247G 315insC T489C G709A A769G A825T A1018G A1041G (2105-2122)missing A2216N A2758G C2885T T3394C T3594C G4104A T4312C G4491A T5029C G7146A T7256C A7521G T8468C T8655C (10331-10335)missing C10400T T10664C A10688G C10810T T10824N A10890N C10915T A11914G C12362T G13105A G13276A T13506C T13650C T14102N T14146N T14308C T14783C G15043A G15301A A15671G A16129G A16158G T16187C C16189T G16230A C16234T T16278C C16311T T16362C T16519C |
| SRR043142 | C146T C195T A247G G275N C285N (292-316)missing T489N (568-588)missing G709A A769G A825T A1018G C1436N A1482N G1485N T1998N T2010N G2094N C2508N C2511N (2521-2560)missing T2563N A2694N C2703N (2711-2765)missing C2872N G2878N C2885T G3337N T3594C G4104A T4312C A4833G T5108C C5601T (5732-5756)missing G5864N C6535N G7013N G7146A T7256C A7521G G7600A T8468C T8655C A9377G C9449N G9477N G9575A C10400T G10644T (10649-10683)missing A10688G C10810T C10915T T11410N A11914G C12852N (12858-12868)missing G13105A G13276A C13383T T13506C A13563G G13643N T13650C G14569A |

| | |
|---|---|
| | T14783C G14861A G15043A G15301A A15562G A16129G T16187C C16189T A16227G G16230A C16234T A16309G C16311T T16362C |
| SRR043143 | C146T C152T A193G C195T A247G 315insC G709A A769G A825T A1018G A2758G C2885T T3594C G4104A T4312C T5465C A5498G G7146A T7256C A7521G 8281delC 8282delC 8283delC 8284delC 8285delC 8286delT 8287delC 8288delT 8289delA T8468C T8655C G8701A G9123A C9540T G10398A T10664C A10688G C10810T C10873T C10915T A11914G T12705C G13105A G13276A T13506C T13650C A13834G C14751T T15262C A16129G T16187C C16189N T16217C T16223C G16230A T16278C A16299G C16311T T16519C |
| SRR043144 | C146T C151T C152N C195T A200G A235G A247G (312-313)missing 315insC A663G A735G A769G A825T A1018G G1598A A1736G A2758G C2885T T3594C G4104A T4248C T4312C A4824G G7146A T7256C A7521G G7805A T8468C T8655C G8701A C8794T C9540T G10398A T10664C A10688G C10810T C10873T C10915T G13105A G13276A T13506C T13650C A16129G T16187C C16189T G16230A T16278C C16290T C16311T G16319A T16362C |
| SRR043145 | C146T C152T C195T A247G C306N 315insC T489C T711C A769G A825T A1018G A1041G A2758G C2885T T3394C T3594C G4104A T4312C G4491A 5899insC T7142C G7146A T7256C A7521G G7697A T8468C T8655C A9242G C10400T T10664C A10688G C10810T C10915T A11914G G13105A G13276A T13506C T13650C T14308C A14417G T14783C G15043A G15301A A16129G C16169T C16184T T16187C C16189T G16230A C16234T T16278C C16311T A16316G T16362C |
| SRR043146 | C146T C152T C195T C198T A247G T489C T711C A769G A825T A1018G A1041G A2758G C2885T T3338C T3394C G3438A T3594C C3992T G4104A T4312C G4491A (5885-5902)missing T7142C G7146A T7256C A7521G G7697A T8468C T8655C T9098C A9242G A9632G |

| | |
|---|---|
| | G9921A C10400T T10664C A10688G C10810T C10915T A11914G C12112T G13105A G13276A T13506C T13650C T14308C A14417G T14783C G15043A G15301A A16129G T16187C C16189T G16230A C16234T T16278C C16311T A16316G T16362C |
| SRR043147 | C146T C150T (151-152)missing A153G C195T A247G 315insC T489C A769G A825T A1018G A1041G A2758G C2885T T3394C T3594C G4104A T4312C G4491A G7146A T7256C A7521G G7754A T8468C T8655C C10400T T10664C A10688G C10810T C10915T A11914G C12362T G13105A G13276A T13506C T13650C T14308C T14783C G15043A G15119A G15301A A15671G A16129G A16158G T16187C C16189T G16230A C16234T T16278C C16311T T16362C T16519C |
| SRR043148 | C146T C152T C195T A247G T489C T711C A769G A825T A1018G A1041G A2758G C2885T T3394C T3594C G4104A T4312C G4491A A5894C 5899insC T7142C G7146A T7256C A7521G G7697A T8468C T8655C A9242G C10400T T10664C A10688G C10810T C10915T A11914G G13105A G13276A T13506C T13650C T14308C A14417G T14783C G15043A G15301A A16129G T16187C C16189T G16230A C16234T T16278C C16311T A16316G T16362C |
| SRR043149 | C146T C195T A247G 315insC T489C G709A A769G A825T A1018G A2758G C2885T T3594C G4104A T4312C A4833G T5108C C5601T G7146A T7256C A7521G G7600A T8468C T8655C A9377G G9575A C10400T T10664C A10688G C10810T C10915T A11914G G13105A G13276A C13383T T13506C A13563G T13650C G14569A T14783C G14861A G15043A G15301A A15562G A16129G T16187C C16189T A16227G G16230A C16234T A16309G C16311T T16362C |
| SRR043150 | A247G 315insC T489C T710C A769G A825T A1018G T1193C C1342T A2758G C2885T T3594C G4104A T4312C A5582C T6253C G7146A T7256C A7521G G8269A T8468C T8655C C10400T T10664C A10688G C10810T C10915T C11810T A11914G T12732C G13105A |

| | |
|---|---|
| | G13276A T13356C T13506C T13650C T14783C G15043A G15301A A16129G C16176T T16187C C16189T G16230A C16260T C16270T T16278C C16311T T16362C T16381C T16519C |
| SRR043151 | C146T C152T C195T C198T A247G (291-309)missing 315insC T489C T711C A769G A825T A1018G A1041G A2758G T2778N C2885T T3338C T3394C G3438A T3594C C3992T G4104A T4312C G4491A A5894N T7142C G7146A T7256C A7521G G7697A T8468C T8655C T9098C A9242G A9632G G9921A C10400T T10664C A10688G C10810T C10915T A11914G C12112T G13105A G13276A T13506C T13650C T14308C A14417G T14783C G15043A G15301A A16129G T16187C C16189T G16230A C16234T T16278C C16311T A16316G T16362C |
| SRR043152 | C146T C152T C195T A247G 315insC T489C T593C G709A A769G A825T A1018G G1719A A2758G C2885T T3594C G4104A T4312C T4353C A4833G T5108C G5460A G7146A T7256C A7521G T8200C T8468C T8655C G9966A C10400T T10664C A10688G C10810T C10915T C11008T A11914G A12361G C12663T G13105A G13276A T13506C T13650C A14079G T14287C G14569A T14783C G15043A G15301A G15323A G15497A A16129G T16187C C16189T G16230A T16278C C16311T T16362C T16519C |
| SRR043153 | C146T C152T A247G T489C G709A A769G A825T A1018G A2758G C2885T T3594C G4104A T4312C A4833G T5078C T5108C C5601T G7146A T7256C A7521G C7533T G7600A T8468C T8655C A9377G G9575A C10400T T10664C G10680A A10688G C10810T C10915T A11914G G13105A G13276A T13506C A13563G T13650C T14200C G14569A T14783C G15043A G15301A A16129G T16172C T16187C C16189N A16227G G16230A C16311T T16362C |
| SRR043154 | C146T C152N C195T A234N A247G T489C G709A A769G A825T A1018G A2758G C2885T A3447N T3594C G4104A T4312C A4833G |

| | |
|---|---|
| | G4853A T5108C C5601T G7146A T7256C A7521G A8107N T8468C T8655C T8877C C10400T T10664C A10688G C10810T C10915T A11914G T12375C G13105A G13194N G13276A T13506C A13563G T13650C G14569A T14783C C14902N G15043A G15301A A16129G T16187C C16189T G16230A A16269G T16278C C16287N C16311T T16362C |
| SRR043156 | G103A C146T C152T C195T A247G C305N C312N 315insC (518-520)missing G709A A769G A825T 960insC A1018G G1598A A2758G C2885T T3594C G4104A T4312C T4418C T4947C C6101T G7146A T7256C T7429N (7437-7445)missing G7453N A7521G (7575-7582)missing (8260-8309)missing T8468C G8584A T8655C G8701A A8784G C8829T C9458N C9540T T9950C (10163-10173)missing T10664C A10688G C10810T C10873T C10915T A11914G A12361G T12705C G13105A G13276A T13506C T13650C G14384A G15077A C15223T C15508T A15662G A15851G G15927A A16129G T16140C A16182N T16187C T16223C G16230A T16243C T16278C C16311T |

**Table S3. Haplogroup frequencies in 7 populations from the Tibetan Plateau**

| Haplogroup | Dengs (n=91) | | Lhobas (n=91) | | Tingri Tibetans (n=86) | | Monpas (n=17) | | Sherpas (n=76) | | Tibetan1 (n=38) | | Tibetan2 (n=33) | | Total (n=432) | |
|---|---|---|---|---|---|---|---|---|---|---|---|---|---|---|---|---|
| | n | % | n | % | n | % | n | % | n | % | n | % | n | % | n | % |
| A | 2 | 2.19 | 15 | 16.48 | 11 | 12.79 | 3 | 17.64 | 20 | 26.31 | 5 | 13.15 | 3 | 9.09 | 59 | 13.65 |
| B4'5 | 1 | 1.09 | 1 | 1.09 | 2 | 2.32 | 0 | 0.00 | 0 | 0.00 | 2 | 5.26 | 2 | 6.06 | 8 | 1.85 |
| R11 | 0 | 0.00 | 0 | 0.00 | 1 | 1.16 | 0 | 0.00 | 0 | 0.00 | 0 | 0.00 | 0 | 0.00 | 1 | 0.23 |
| F | 4 | 4.39 | 24 | 26.37 | 9 | 10.46 | 0 | 0.00 | 1 | 1.31 | 4 | 10.52 | 2 | 6.06 | 44 | 10.18 |
| Y | 0 | 0.00 | 0 | 0.00 | 1 | 1.16 | 0 | 0.00 | 0 | 0.00 | 0 | 0.00 | 0 | 0.00 | 1 | 0.23 |
| R22 | 0 | 0.00 | 1 | 1.09 | 0 | 0.00 | 0 | 0.00 | 0 | 0.00 | 0 | 0.00 | 0 | 0.00 | 1 | 0.23 |
| H | 0 | 0.00 | 0 | 0.00 | 0 | 0.00 | 0 | 0.00 | 1 | 1.31 | 0 | 0.00 | 0 | 0.00 | 1 | 0.23 |
| U | 0 | 0.00 | 0 | 0.00 | 3 | 3.48 | 0 | 0.00 | 4 | 5.26 | 1 | 2.63 | 0 | 0.00 | 8 | 1.85 |
| W | 0 | 0.00 | 0 | 0.00 | 0 | 0.00 | 0 | 0.00 | 1 | 1.31 | 0 | 0.00 | 0 | 0.00 | 1 | 0.23 |
| M9 | 36 | 39.56 | 15 | 16.48 | 31 | 36.04 | 7 | 41.17 | 14 | 18.42 | 6 | 15.78 | 8 | 24.24 | 117 | 27.08 |
| D4 | 14 | 15.38 | 10 | 10.98 | 4 | 4.65 | 1 | 5.88 | 5 | 6.57 | 4 | 10.52 | 7 | 21.21 | 45 | 10.41 |
| D5'6 | 8 | 8.79 | 11 | 12.08 | 6 | 6.97 | 4 | 23.52 | 3 | 3.94 | 2 | 5.26 | 0 | 0.00 | 34 | 7.87 |
| C4 | 2 | 2.19 | 3 | 3.29 | 1 | 1.16 | 0 | 0.00 | 14 | 18.42 | 2 | 5.26 | 1 | 3.03 | 23 | 5.32 |
| Z | 9 | 9.89 | 0 | 0.00 | 1 | 1.16 | 0 | 0.00 | 0 | 0.00 | 1 | 2.63 | 0 | 0.00 | 11 | 2.54 |
| M12'G | 4 | 4.39 | 5 | 5.49 | 5 | 5.81 | 2 | 11.76 | 1 | 1.31 | 5 | 13.15 | 7 | 21.21 | 29 | 6.71 |
| M10 | 0 | 0.00 | 0 | 0.00 | 2 | 2.32 | 0 | 0.00 | 0 | 0.00 | 0 | 0.00 | 1 | 3.03 | 3 | 0.69 |
| M11 | 1 | 1.09 | 2 | 2.19 | 0 | 0.00 | 0 | 0.00 | 1 | 1.31 | 1 | 2.63 | 1 | 3.03 | 6 | 1.38 |
| M13 | 0 | 0.00 | 1 | 1.09 | 5 | 5.81 | 0 | 0.00 | 2 | 2.63 | 2 | 5.26 | 0 | 0.00 | 10 | 2.31 |
| M3 | 0 | 0.00 | 0 | 0.00 | 0 | 0.00 | 0 | 0.00 | 3 | 3.94 | 0 | 0.00 | 0 | 0.00 | 3 | 0.69 |
| M33 | 8 | 8.79 | 0 | 0.00 | 0 | 0.00 | 0 | 0.00 | 0 | 0.00 | 0 | 0.00 | 0 | 0.00 | 8 | 1.85 |
| M4"67 | 0 | 0.00 | 0 | 0.00 | 0 | 0.00 | 0 | 0.00 | 3 | 3.94 | 1 | 2.63 | 0 | 0.00 | 4 | 0.92 |
| M49 | 1 | 1.09 | 0 | 0.00 | 0 | 0.00 | 0 | 0.00 | 0 | 0.00 | 0 | 0.00 | 0 | 0.00 | 1 | 0.23 |
| M5 | 0 | 0.00 | 0 | 0.00 | 2 | 2.32 | 0 | 0.00 | 2 | 2.63 | 0 | 0.00 | 0 | 0.00 | 4 | 0.92 |
| M60 | 0 | 0.00 | 3 | 3.29 | 0 | 0.00 | 0 | 0.00 | 0 | 0.00 | 0 | 0.00 | 0 | 0.00 | 3 | 0.69 |
| M61 | 0 | 0.00 | 0 | 0.00 | 0 | 0.00 | 0 | 0.00 | 0 | 0.00 | 0 | 0.00 | 1 | 3.03 | 1 | 0.23 |
| M62 | 1 | 1.09 | 0 | 0.00 | 2 | 2.32 | 0 | 0.00 | 0 | 0.00 | 1 | 2.63 | 0 | 0.00 | 4 | 0.92 |
| M70 | 0 | 0.00 | 0 | 0.00 | 0 | 0.00 | 0 | 0.00 | 1 | 1.31 | 1 | 2.63 | 0 | 0.00 | 2 | 0.46 |

## Table S4 Genetic diversities of 7 populations of the Tibetan Plateau

| Population | *n* | $\pi\times10^{-3}$ | $\theta\times10^{-3}$ | TajimaD | SigD | FuLiD* | SigD | FuLiF* | SigF |
|---|---|---|---|---|---|---|---|---|---|
| Tingri Tibetans | 86 | 1.492 | 4.152 | -2.1954 | ** | -4.0454 | ** | -3.9102 | ** |
| Dengs | 91 | 1.113 | 3.191 | -2.1693 | ** | -3.2881 | ** | -3.3851 | ** |
| Lhobas | 91 | 1.517 | 3.119 | -1.7397 | * | -2.0067 | * | -2.2718 | * |
| Monpas | 17 | 1.157 | 1.399 | -0.7287 | n.s. | -0.5282 | n.s. | -0.6793 | n.s. |
| Sherpas | 76 | 1.649 | 3.382 | -1.7726 | * | -3.2364 | * | -3.1571 | ** |
| Tibetan1 | 38 | 1.599 | 3.962 | -2.2379 | ** | -3.3435 | ** | -3.5132 | ** |
| Tibetan2 | 33 | 1.546 | 3.157 | -1.9453 | * | -2.6409 | * | -2.8506 | * |

# Table S5 Coalescence time of 22 highlander-specific lineages

| Haplogroup | *n* | ρ-based Method (kya) | ML Method (kya) |
|---|---|---|---|
| A11a | 24 | 8.45±2.23 | 7.54±2.78 |
| A15c1a | 14 | 1.66±0.88 | 1.33±1.13 |
| M9a1a2 | 23 | 7.89±2.65 | 8.51±9.70 |
| C4a3b1 | 11 | 0.70±0.40 | 0.82±0.61 |
| C7a1a2 | 5 | 2.06±1.03 | 3.20±2.16 |
| M9a1b1d3 | 32 | 1.93±0.92 | 1.23±0.72 |
| F1c1a1a | 5 | 6.28±1.79 | 7.43±2.26 |
| M49a1a1 | 11 | 0.23±0.23 | 0.31±0.31 |
| G3b1a | 3 | 7.00±2.99 | 6.36±2.99 |
| M13a2 | 13 | 14.66±4.47 | 10.68±3.10 |
| M62 | 22 | 26.41±4.46 | 24.24±5.09 |
| M9a1a1c1b | 88 | 10.95±3.60 | 9.48±4.35 |
| M9a1b1c | 16 | 7.39±2.13 | 6.79±1.85 |
| D4j1b3 | 4 | 6.55±3.17 | 7.11±4.25 |
| D5a2c2 | 8 | 3.57±2.28 | 4.46±11.55 |
| A7 | 5 | 4.16±3.04 | 5.83±2.47 |
| M7b1a1j | 4 | 5.22±2.12 | 4.25±3.83 |
| D5a3a2 | 4 | 13.37±1.93 | 12.99±4.98 |
| M33b1a1 | 8 | 1.29±0.64 | 1.54±1.13 |
| G3b3a | 3 | 5.22±4.60 | 6.36±4.67 |
| N11a1 | 4 | 10.61±4.88 | 13.43±4.98 |
| Z7 | 6 | 6.10±2.44 | 8.18±2.89 |

**Table S6 Comparison on the NS/S between the internal lineages of Tibetan highlanders (1≤ρ≤4) and the contemporary haplogroups of all the 36,914 sequences in all protein-coding genes and four complexes respectively**

| | Tibetan highlanders | | | All sequences | | | | |
|---|---|---|---|---|---|---|---|---|
| Complex | N | S | N/S | N | S | N/S | P[a] | Sig[b] |
| Complex I | 21 | 33 | 0.64 | 1536 | 3698 | 0.42 | 0.134 | |
| Complex III | 2 | 8 | 0.25 | 438 | 663 | 0.66 | 0.331 | |
| Complex IV | 9 | 16 | 0.56 | 512 | 1626 | 0.31 | 0.162 | |
| Complex V | 11 | 5 | 2.20 | 547 | 492 | 1.11 | 0.219 | |
| all | 43 | 62 | 0.69 | 3033 | 6479 | 0.47 | 0.058 | # |

Note: [a] P value calculated by Fisher Exact Test, [b] # denotes0.050< p<0.100.

# Reference


1 Zheng, H. X. *et al.* Major Population Expansion of East Asians Began before Neolithic Time: Evidence of mtDNA Genomes. *PLoS One* **6**, e25835 (2011).

2 Zheng, H.-X., Yan, S., Qin, Z.-D. & Jin, L. MtDNA analysis of global populations support that major population expansions began before Neolithic Time. *Sci. Rep.* **2** (2012).

3 Ji, F. *et al.* Mitochondrial DNA variant associated with Leber hereditary optic neuropathy and high-altitude Tibetans. *Proc. Natl. Acad. Sci. USA* **109**, 7391-7396 (2012).

4 Derenko, M. *et al.* Phylogeographic analysis of mitochondrial DNA in northern Asian Populations. *Am. J. Hum. Genet.* **81**, 1025-1041 (2007).

5 Ingman, M. & Gyllensten, U. Rate variation between mitochondrial domains and adaptive evolution in humans. *Hum. Mol. Genet.* **16**, 2281-2287 (2007).

6 Kang, L. *et al.* mtDNA lineage expansions in Sherpa population suggest adaptive evolution in Tibetan highlands. *Mol. Biol. Evol.* **30**, 2579-2587 (2013).

7 Chandrasekar, A. *et al.* Updating Phylogeny of Mitochondrial DNA Macrohaplogroup M in India: Dispersal of Modern Human in South Asian Corridor. *PLoS One* **4**, e7447 (2009).

8 Kong, Q. P. *et al.* Large-scale mtDNA screening reveals a surprising matrilineal complexity in East Asia and its implications to the peopling of the region. *Mol. Biol. Evol.* (2010).

9 Peng, M. S. *et al.* Inland post-glacial dispersal in East Asia revealed by mitochondrial haplogroup M9a'b. *BMC Biol.* **9**, 2 (2011).

10 Qin, Z. D. *et al.* A Mitochondrial Revelation of Early Human Migrations to the Tibetan Plateau Before and After the Last Glacial Maximum. *Am. J. Phys. Anthropol.* **143**, 555-569 (2010).

11 Yi, X. *et al.* Sequencing of 50 human exomes reveals adaptation to high altitude. *Science* **329**, 75-78 (2010).

12 Zhao, M. *et al.* Mitochondrial genome evidence reveals successful Late Paleolithic settlement on the Tibetan Plateau. *Proc. Natl. Acad. Sci. USA* **106**, 21230-21235 (2009).
